# Mansouramycins E–G, Cytotoxic Isoquinolinequinones from Marine Streptomycetes

**DOI:** 10.3390/md19120715

**Published:** 2021-12-20

**Authors:** Mohamed Shaaban, Khaled A. Shaaban, Gerhard Kelter, Heinz Herbert Fiebig, Hartmut Laatsch

**Affiliations:** 1 Institute of Organic and Biomolecular Chemistry, University of Göttingen, Tammannstrasse 2, D-37077 Göttingen, Germany or ms.attia@nrc.sci.eg (M.S.); khaled_shaaban@uky.edu (K.A.S.); 2 National Research Centre, Chemistry of Natural Compounds Department, Pharmaceutical and Drug Industries Research Institute, El-Behoos St. 33, Giza 12622, Egypt; 3 Oncotest GmbH, Charles River Discovery Germany, Am Flughafen 14, D-79108 Freiburg, Germany; Gerhard.Kelter@crl.com; 4 Oncotest GmbH, Biotec GmbH, Am Flughafen 14, D-79108 Freiburg, Germany; fiebig@4hf.eu

**Keywords:** mansouramycins, isoquinolinequinones, marine-derived *Streptomyces* sp., cytotoxicity

## Abstract

Chemical investigation of the ethyl acetate extract from the marine-derived *Streptomyces* sp. isolate B1848 resulted in three new isoquinolinequinone derivatives, the mansouramycins E–G (**1a**–**3a**), in addition to the previously reported mansouramycins A (**5**) and D (**6**). Their structures were elucidated by computer-assisted interpretation of 1D and 2D NMR spectra, high-resolution mass spectrometry, and by comparison with related compounds. Cytotoxicity profiling of the mansouramycins in a panel of up to 36 tumor cell lines indicated a significant cytotoxicity and good tumor selectivity for mansouramycin F (**2a**), while the activity profile of E (**1a**) was less attractive.

## 1. Introduction

The first natural isoquinolinequinone isolated from bacteria were reported by Fukum et al. in 1977 [1] and then by Kubo et al. in 1988 [2]. A few others were isolated from porifera, including cribrostatins (produced by the blue marine sponge *Cribrochalina* sp.) [3], renierones (from *Reniera*, *Petrosia*, and *Haliclona* spp.) [4,5], and caulibugulones (found in the marine bryozoon *Caulibugula inermis)* [6]. These isoquinolinequinones showed a potent antimicrobial activity against Gram positive bacteria and yeast (*Candida albicans*) and a pronounced cytotoxicity against L1210 and other cell lines with IC_50_ values as low as 30 ngmL^−1^ [7,8]. In 1998, we isolated mansouramycin A (**5**) as a trace component from the marine derived *Streptomyces* sp. B3497 [9]. Mansouramycin A (**5**) and the synthetic analogue 3-methyl-7-(methylamino)-5,8-isoquinolinedione (**4a**) were re-isolated from the marine-derived *Streptomyces* sp. isolate Mei37, together with three new mansouramycins B–D (**1a**–**3a**) [10]. These compounds showed a pronounced selectivity for non-small cell lung cancer, breast cancer, melanoma, and prostate cancer cells. Recently, mansouramycin A (**5**) was also obtained from the marine-derived *Streptomyces albus* J1074 and found to be a potent inhibitor of the methicillin-resistant *Staphylococcus aureus* ATCC 43300 with an MIC of 8 μgmL^−1^. *S. albus* J1074 produced additionally the novel isoindoloquinone albumycin [11].

**Figure 1:**
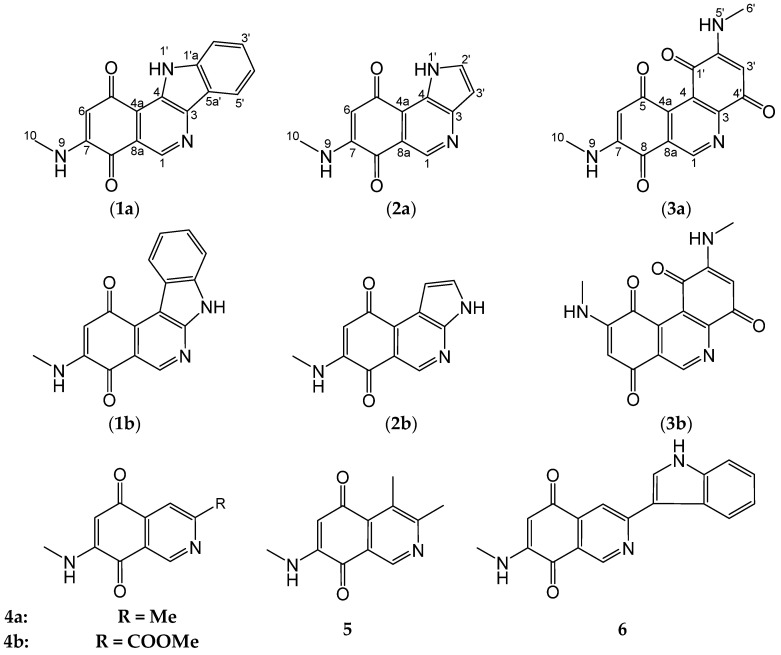
Chemical structures of isoquinolinequinones **1–6** produced by *Streptomyces* sp. B1848, and alternative structures **1b–3b**.

While the marine-derived *Streptomyces* sp. isolate B1848 was previously noted as a producer of 6-hydroxy-isatine and several other known compounds [12,13,14], further fermentations led now to the isolation and characterization of three unusual mansouramycins E–G (**1a**–**3a**) along with mansouramycins A (**5**), D (**6**) (Figure 1) and 13 known metabolites [12,13,14]. The chemical structures of **1a**–**3a** were elucidated by NMR (1D, 2D) and HRMS, by comparison with related compounds and by computer-assisted methods. The cytotoxic activity of the isolated isoquinolinequinones was determined.

## 2. Results and Discussion

With a malt extract medium with 50% synthetic seawater (M_2_^+^ medium), the marine-derived *Streptomyces* sp. isolate B1848 produced only traces of mansouramycins A (**5**) and D (**6**), along with the zizaene derivative albaflavenol [9], 6-hydroxy-isatine [13,14], 2′-deoxythymidine, 2′-deoxuridine, 2′-deoxyadenonsine, anthranilic acid, tyrosol, indolyl-3-acetic acid, phenyl acetamide, indolyl-3-carboxylic acid, *N*^ß^-acetyltryptamine, *N*-acetyltyramine, and *p*-hydroxybenzoic acid [12]. Better yields of the mansouramycins and further red pigments were obtained now on a meat extract medium in a fermentation with a 50 L shaker culture. After extraction and chromatographic separation, the strain B1748 afforded under these conditions the mansouramycins A (**5**) and D (**6**) and three new congeners, the mansouramycins E–G (**1a**–**3a**) as dark red solids. The isoquinolinequinones gave brown-red zones on TLC, with UV absorptions in solution similar as of *peri*-hydroxyquinones. Their reversible color change with sodium dithionite from orange to nearly colorless confirmed quinones; *peri*-hydroxyquinones were excluded, however, by the missing bathochromic shift with sodium hydroxide. Unlike the orange-red phenoxazinone chromophore of actinomycins and related pigments, which are becoming red with concentrated sulfuric acid, the isoquinolinequinones turned yellow. Further physicochemical properties of compounds **1a**−**3a** are summarized in Table 1.

### 2.1. Structure Elucidation

Compound **1a** was obtained as red powder of moderate polarity. The molecular formula was determined as C_16_H_11_N_3_O_2_ by EI-HRMS, indicating 13 double bond equivalents (DBE). The color change to yellow with concentrated sulfuric acid and the characteristic UV curve with a flat absorption at λ_max_ 509 nm as for **5**, **6** pointed to an isoquinolinequinone moiety as well [10] (Table 1). The ^13^C NMR spectrum (Table 2) showed six aromatic/olefinic methines and one methyl signal. Furthermore, signals of nine non-protonated carbon atoms were observed, of which two at *δ* 182.8 and 181.4 pointed to carbonyl groups of a quinone. In the proton NMR spectrum (Table 2), the CH singlets at *δ* 9.01 (H-1) and 5.71 (H-6), in addition to a broadened NH signal at *δ* 7.83 and a methyl doublet at *δ* 2.85 of the CH_3_NH fragment were typical for mansouramycins.

**Figure 2:**
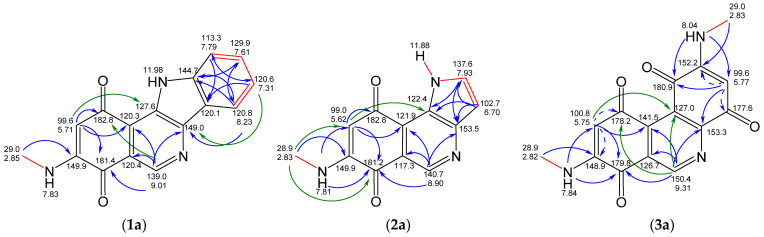
2D NMR correlations of mansouramycins E–G (**1a**–**3a**). Blue arrows = ^2^*J*, ^3^*J* HMBC correlations; green arrows = ^4^*J* HMBC correlations, red bonds = COSY correlations.

The proton H-6 showed HMBC correlations (Figure 2) with C-4 (^4^*J*), 4a, 5, 7, and C-8; correlations of the N-methyl signal with C-7, and of H-1 with C-3, 4a and 8 resulted in a 3,4-disubstitued 7-methylamino-isoquinolinequinone skeleton as in **4a**–**6**; unfortunately, NH HMBC correlations were not visible for **1a**.

A 1,2-disubstituted benzene ring was deduced from the typical signal pattern of four *o*,*m*-coupled protons at *δ* 8.23 (d), 7.79 (d), 7.61 (td), and 7.31 (td) ppm and from the expected HMBC correlations (Figure 2). A further broadened NH signal was seen at *δ* 11.98, which formed with the remaining atoms an aniline residue. With respect to the two open valencies in both fragments, the isoquinoline and the aniline unit can be merged only in two ways under formation of structures **1a** or **1b**. The more in-depth analysis of the NMR data by means of the structure elucidation program COCON [15] confirmed isomers **1a** and **1b** as allowed structures, but delivered >7600 additional alternatives! Most of them were highly strained (cyclobutenes, non-linear allenes, or bridged aromatic systems) and therefore excluded.

Amongst 24 plausible indoloquinoline- and indoloisoquinoline-quinones, only 6 (**1a**, **1b**, **S1c**, **S1e**, **S1f**, **S1h**) * were found by COCON and therefore only these are in agreement with the COSY and HMBC correlations. For mansouramycin E, the isomer **1a** showed the best agreement of experimental NMR data with shifts calculated by SPARTAN’20 [16] using ab initio methods on a high level of theory. This structure was therefore assumed for mansouramycin E (see Supplementary Materials). Further applications of this technique have been described previously [17]. * Formula numbers with a leading bold letter “**S**” are refering to structures in Supplementary Materials.

For the dark red mansouramycin F (**2a**) the molecular formula C_12_H_9_N_3_O_2_ (ESI-HRMS) was determined, which entails 10 DBE. The ^1^H and ^13^C NMR shifts (Table 2), as well as the HMBC couplings, confirmed again an *N*-methyl-isoquinolinequinone substructure as in all other mansouramycins (Figure 1). According to the chemical shifts and 2D correlations, the unassigned residual atoms C_2_H_3_N were belonging to an annulated pyrrole ring, which was confirmed by the 1H triplet at *δ*_H_ 7.93 and the dd signal at 6.70 with the expected small coupling constants (~5 Hz). The pyrrole ring can be fused with the isoquinolinequinone core in three different ways, yielding structures **2a**, **2b**, **S2m**, and the respective isomers with the N-methyl group at C-6 instead at C-7 (see Supplementary Materials). With COCON using atom types, 19 isomers were found. Four of them were quinones (**2a**, **2b**, **S2c**, **S2d**). The other structures were azepin-2-ones or highly strained bridged systems. Isomers of type **S2m** were excluded by COCON as well, and also *o*-quinones were not predicted for mansouramycin F.

H-3′ in **2b** should show a ^3^*J* correlation with C-4a, which is missing in the experimental spectrum and therefore better fitting on **2a**. In **S2c**/**S2d**, the quinonoid proton H-7 (*δ* ~5.6) should show a ^3^*J* correlation with C-8a at *δ* ~147. However, this was also not observed, so that only structure **2a** was left. For further confirmation, we compared the experimental with calculated shifts of all possible pyrrolo-quinoline- and pyrroloisoquinoline-5,8-quinones. The results (Table S2) pointed again clearly to structure **2a** for mansouramycin F. This conclusion was further confirmed by comparison with similarly fused pyrrolo-pyridine skeletons [18,19].

Compound **3a** was obtained as a red solid as well, which displayed isoquinoline-quinone-like UV/vis and other physicochemical properties. The molecular formula of **3a** was established as C_15_H_11_N_3_O_4_ by ESI-HRMS and ^1^H and ^13^C NMR analysis, entailing 12 DBE. The ^1^H and ^13^C NMR spectra confirmed a further isoquinoline-quinone, which showed, however, remarkable differences compared with **1a** and **2a**. Instead of one *N*-methyl residue (7-NHCH_3_) and one quinonoid proton (6-H), as in the other mansouramycins, the ^1^H NMR spectrum showed each two of these signals. In addition, the ^13^C NMR spectrum displayed four carbonyl groups (*δ*_C_ 178.2, 179.8, 177.6, and 180.9) instead of two carbonyls as in **1a** and **2a** (Table 2). Interpretation of the HMBC spectrum of **3a** (Figure 2) revealed an isoquinoline-quinone and an *N*-methylaminobenzoquinone substructure, which can be connected in two different ways only, resulting in **3a** or **3b**, respectively (Figure 1 and Figure 2). The alternative **3b** was excluded, however, based on the significant ^3^*J* HMBC correlations of H-6 (*δ*_H_ 5.75) and NH-9 (*δ*_H_ 7.84) with CO-8 (*δ*_C_ 179.8) and not with CO-5 (*δ*_C_ 178.2); the position of the second *N*-methyl group at C-3′ was determined in a similar way. All the remaining HMBC and COSY correlations (Figure 2) were in full agreements with structure **3a**, a novel azaphenanthrene diquinone, which we named mansouramycin G (see also Supplementary Materials).

### 2.2. Biological Activities

Isolated compounds were evaluated in cytotoxicity assays against the same 36 cancer cell lines as published before [10]. Consistent with results previously reported herein for other members of the group, cytotoxicity profiling of the new mansouramycin **2a** revealed good anti-tumor activity in vitro with a mean IC_50_ value of 7.92 μM (1.797 µgmL^−1^). Furthermore, **2a** showed good tumor selectivity across the panel of 36 cell lines. Mansouramycin E (**1a**) was less active and selective [mean IC_50_ = 23.10 μM (6.398 µgmL^−1^)]. Previously reported mansouramycins C (**4b**) and A (**5**) exhibited mean IC_50_ values of 0.089 μM (0.022 µgmL^−1^) and 13.44 μM (2.902 µgmL^−1^), respectively (Table 3). Mansouramycin G (**3a**) was not tested, due to a lack of material. In the agar diffusion test, crude extracts of *S.* isolate B1848 exhibited high bioactivity against *Mucor miehei* (Tü 284) and *Candida albicans*, and moderate activity against *Escherichia coli* and the alga *Chlorella vulgaris*. The samples of **1**–**6** were nearly consumed in the cytotoxicity assays and therefore not tested for their antimicrobial activity.

## 3. Materials and Methods

### 3.1. General Procedures

NMR spectra were measured on Varian Unity 300 and Varian Inova 600 spectrometers. The spectra were referenced to the signals of partially deuterated solvents (*δ*_Chl_ 7.270, 77.000; *δ*_DMSO_ 2.500, 39.510). Electron spray ionization mass spectrometry (ESI HRMS): Finnigan LCQ ion trap mass spectrometer coupled with a Flux Instruments (Basel, Switzerland) quaternary pump Rheos 4000 and a HP 1100 HPLC (nucleosil column EC 125/2, 100-5, C 18) with autosampler (Jasco 851-AS, Jasco Inc., Easton, MD, USA) and a Diode Array Detector (Finnigan Surveyor LC System). High resolution mass spectra (HRMS) were recorded by ESI MS on an Apex IV 7 Tesla Fourier-Transform Ion Cyclotron Resonance Mass Spectrometer (Bruker Daltonics, Billerica, MA, USA). EI mass spectra (70 eV) were recorded on a Finnigan MAT 95 spectrometer (Thermo Electron Corp., Bremen, Germany) with perfluorokerosene as reference substance for EI HRMS. IR spectra were recorded on a Perkin-Elmer 1600 Series FT-IR spectrometer from KBr pellets. UV/vis spectra were recorded on a Perkin-Elmer Lambda 15 UV/vis spectrometer. Flash chromatography was carried out on silica gel (230–400 mesh). *R*_f_-values were measured on Polygram SIL G/UV_254_ (Macherey-Nagel & Co., Düren, Germany). Size exclusion chromatography was carried out on Sephadex LH-20 (Lipophilic Sephadex; Amersham Biosciences, Ltd., purchased from Sigma-Aldrich Chemie, Steinheim, Germany).

### 3.2. Isolation and Taxonomy of the Producing Strain

The marine *Streptomyces* sp. strain B1848 was isolated and deposited in the Actinomycetes culture collection of the Alfred-Wegner Institute for Polar- und Marine Research, Am Handelshafen, Bremen, Germany. The taxonomy of the strain has been described previously [12].

### 3.3. Fermentation and Working Up

The *S.* sp. isolate B1848 was previously cultivated on M_2_^+^ medium with 50% seawater in a 25 L jar fermenter (72 h at 28 °C) [12,13]. Optimization of the culture conditions has been performed now using six different media [14] at two pH values (6.5, 7.8), temperatures (28, 35 °C), and shaking rates (110, 95 rpm) for four days. TLC analysis and antimicrobial screenings indicated that medium C (meat extract medium: 10 g glucose, 2 g peptone, 1 yeast, 1 g meat extract, pH 7.8) gave the best yield of mansouramycins.

A 50-L jar fermenter with C-medium was inoculated with strain B1848 and stirred for 4 days at 28 °C with 120 rpm. The resulting pale yellow culture broth was mixed with diatomaceous earth (Celite, ca. 1.8 kg), and filtered-off under pressure. The mycelial cake was extracted with ethyl acetate (3×), and then with acetone (2×). The acetone extract was concentrated under reduced pressure, and the aqueous residue was extracted once more with ethyl acetate. The combined organic phases were concentrated in vacuo, yielding 4.8 g of reddish-orange residue. None of the compounds of interest were detected in the aqueous phases, and therefore they were discarded.

### 3.4. Isolation and Purification

The mycelial cake extract (4.8 g) was applied to flash silica gel column chromatography (3 × 60 cm) using a CH_2_Cl_2_-CH_3_OH gradient. After monitoring by TLC (CHCl_3_/5; 10% MeOH), four fractions were obtained. Purification of fractions II-IV, using PTLC and Sephadex LH 20, led to isolation of five dark red compounds: mansouramycin A (**5**; 3.0 mg), D (**6**, 8.0 mg), E (**1a**; 4.1 mg), F (**2a**; 6.0 mg), and mansouramycin G (**3a**; 4.2 mg); for the physico-chemical properties and NMR spectral data of mansouramycins E–G (**1**–**3**), see Table 1 and Table 2, respectively.

Mansouramycin C (3-Carbomethoxy-7-methylaminoisoquinoline-5,8-dione; **4b**): During this investigation, we realized two errors in the previously reported ^13^C NMR data of mansouramycin D [10]: (CDCl_3_, 150 MHz): *δ* 180.6 (C_q_-8), 179.8 (C_q_-5), 164.3 (C_q_-9), 153.3 (C_q_-3), 148.8 (C_q_-7), 148.0 (CH-1), 140.5 (C_q_-4a), 126.0 (C_q_-8a), 120.7 (CH-4), 101.5 (CH-6), 53.4 (9-OCH_3_), 29.3 (NCH_3_).

Mansouramycin F (7-Methylamino-3*H*-pyrrolo [2,3-c]-isoquinoline-6,9-dione, **2a**): ^1^H NMR (CDCl_3_, 300 MHz): δ 10.25 (s br, 1H, NH-1′), 9.12 (s, 1H, 1-H), 7.79 (t, ^3^*J* = 2.6 Hz, 1H, 2′-H), 6.81 (t, ^3^*J* = 2.9 Hz, 1H, 3′-H), 6.25 (s br, 1H, 9-NH), 5.69 (s, 1H, 6-H), 2.98 (d, *J* = 5.1 Hz, 3H, CH_3_-10).

Data of mansouramycins E–G (**1a**–**3a**) are listed in Table 1, Table 2 and Table 3, and spectra are depicted in the Supplementary Information. The working up and isolation of mansouramycins E–G (**1a**–**3a**) was carried out in August 2004. Spectral measurements, structural interpretation, and biological activity testing of **1a**–**3a** were achieved in the beginning of 2005.

### 3.5. Cytotoxicity Assays

A modified propidium iodide assay was used to examine the antiproliferative activity of the compounds against human tumor cell lines. Cell lines tested were derived from patient tumors engrafted as a subcutaneously growing tumor in NMRI nu/nu mice or obtained from American Type Culture Collection, Rockville, MD, USA, National Cancer Institute, Bethesda, MD, USA, or Deutsche Sammlung von Mikroorganismen und Zellkulturen, Braunschweig, Germany, and details of the test procedure have been described previously [20,21,22]. For the results, see Table 3.

### 3.6. DFT-Calculations

The calculation of NMR shifts was performed in a sequence of six calculation steps implemented in SPARTAN’20 [16]: (1) for all molecules of interest, the least energy conformers were determined using the “systematic approach” of the Merck Molecular Force Field program (MMFF). Up to 500 MMFF conformers within 40 kJ/mol above the global minimum were kept; in step (2), geometries were further optimized with a Hartry-Fock calculation (HF/3-21G); up to 200 conformers with <40 kJ/mol above the global minimum the energies were kept and (3) optimized (energies) with the DFT functional ωB97X-D and the 6-31G* basis set; (4) for up to 100 conformers within a window of 15 kJ/mol, the geometries were calculated now with the same functional and basis set; up to 50 conformers with <10 kJ/mol were kept for step (5); for the remaining conformers, energies and Boltzmann factors (300 K) were calculated with ωB97X-V/6-311+G(2df,2p) [6-311G*]; (6) for up to 30 resulting conformers with <10 kJ/mol the NMR data were calculated with ωB97X-D/6-31G* using the geometries from step (4). The conformer shifts were averaged with the Boltzmann factors from step five.

## 4. Conclusions

Isoquinoline-quinones from marine invertebrates and associated streptomycetes attracted scientific attention due to their strong anticancer activities [1,2,3,4,5,6]. From marine-derived *Streptomyces* spp., we isolated recently five isoquinoline-quinone derivatives, the mansouramycins A-D and 3-methyl-7-(methylamino)-5,8-isoquinolinedione (**4a**), which showed significant cytotoxicity in a panel of up to 36 tumor cell lines, with pronounced selectivity for non-small cell lung cancer, breast cancer, melanoma, and prostate cancer cells [10]. After a culture optimization, we succeeded now to isolate three further mansouramycins E–G (**1a–3a**) from the same marine *Streptomyces* sp. strain B1848, used optimized culture conditions. Their structures were elucidated by computer-assisted interpretation of 1D and 2D NMR spectra, high resolution mass spectrometry, by comparison with *ab initio*-calculated NMR data and by comparison with related compounds. Cytotoxicity profiling of the mansouramycins in a panel of up to 36 tumor cell lines indicated only a moderate cytotoxicity and tumor selectivity for the new quinones E (**1a**) and F (**2a**). The novel azaphenanthrene-diquinone mansouramycin G (**3a**) was not tested due to insufficient material.

## Figures and Tables

**Table 1:** Physico-chemical properties of mansouramycins E–G (**1a**–**3a**).

Analytical Methods	Mansouramycin E (1a)	Mansouramycin F (2a)	Mansouramycin G (3a)
Appearance	Red powder	Dark red solid	Red solid
R*_f_* ^a^	0.76 (CH_2_Cl_2_/7% MeOH)	0.50 (CH_2_Cl_2_/7% MeOH)	0.23 (CH_2_Cl_2_/7% MeOH).
Anisaldehyde/H_2_SO_4_ reagent	yellow	yellow	yellow
Staining with NaOH	no color change	no color change	no color change
Molecular Formula	C_16_H_11_N_3_O_2_	C_12_H_9_N_3_O_2_	C_15_H_11_N_3_O_4_
UV/vis λ_max_ (log ε)	(MeOH): 244 (4.17), 264 (4.20), 287 sh (4.17), 314 sh (3.71), 377 (3.94), 448 sh (3.28), 509 sh (3.17); (MeOH + 1n NaOH): 243 (4.16), 263 (4.21), 287 sh (4.17), 313 sh (3.77) 378 (3.94), 449 sh (3.47), 508 sh (3.17); (MeOH + 1n HCl) 245 sh (4.04), 267 (4.14), 284 sh (4.02) 314 sh (3.31) 387 (3.91), 510 (3.17) nm	(MeOH): 234 (3.66), 288 (3.26), 373 (3.22), 481 sh (2.38); (MeOH+ 1n HCl): 237 (3.53), 313 (3.38), 378 (3.12), 485 sh (2.38) nm; (MeOH+1n NaOH): 233 (3.64), 289 (3.28), 375 (3.19), 485 sh (2.38) nm	(MeOH): 244 (4.13), 299 sh (3.65), 382 (3.42), 435 nm (3.47); (MeOH + 1n HCl): 243 (4.05), 303 (3.65), 377 (3.46), 436 (3.47); (MeOH + 1n NaOH): 245 (4.07), 302 sh (3.57), 384 (3.35), 437 (3.35) nm
IR (KBr) ν_max_ (KBr)	3434, 2925, 2855, 1672, 1625, 1598, 1510, 1491, 1412, 1384, 1354, 1311, 1268, 1208, 1050 cm^−1^	3419, 2926, 2856, 1669, 1595, 1543, 1515, 1489, 1420, 1384, 1336, 1264, 1097, 1028, 764, cm^−1^	3426, 2925, 2855, 1616, 1559, 1544, 1458, 1412, 1384, 1325, 1261, 1028 cm^−1^
CI-MS: *m/z* (%)		245.0 ([M+NH_4_]^+^, 5), 228.0 ([M+H]^+^, 100)	
(+)-ESI-MS: *m/z* (%)	278 ([M+H]^+^)		320.2 ([M+Na]^+^, 31), 617.0 ([2M+Na]^+^, 100)
EI-MS: *m/z* (%)	277 [M]^+^ (84), 256 (8), 249 (12), 236 (15), 220 (11), 195 (8), 192 (13), 179 (9), 166 (24), 138 (13), 102 (8), 97 (15), 82 (28), 73 (36), 69 (42), 57 (72), 43 (76), 44 (100)	227 ([M]^+.^, 100), 199 ([M-CO]^+.^, 8), 186 (16), 145 (9), 116 (8), 59 (12), 43 (8)	
(+)-ESI-HRMS: *m/z*		228.07663 [M+H]^+^	298.08203 [M+H]^+^
Calcd.	277.0846 for C_16_H_11_N_3_O_2_	228.07667 for C_12_H_10_N_3_O_2_ [M+H]^+^	298.08223 for C_15_H_12_N_3_O_4_ [M+H]^+^
EI HRMS: *m/z*	277.0848		

^a^ Silica gel G/UV_254_; **1b, 2b, 3b** (CH_2_Cl_2_/7% MeOH); sh = shoulder.

**Table 2:** ^13^C (150 MHz) and ^1^H NMR spectroscopic data of compounds **1a**–**3a** in DMSO-*d*_6_ (*δ* in ppm, *J* in [Hz]).

Position	Mansouramycin E (1a)		Mansouramycin F (2a)		Mansouramycin G (3a)	
	*δ*_C_, Type	*δ*_H_ (Mult, *J* in [Hz]) ^(a)^	*δ*_C_, Type	*δ*_H_ (Mult, *J* in [Hz]) ^(b)^	*δ*_C_, type	*δ*_H_ (Mult, *J* in [Hz]) ^(a)^
1	139.0, CH	9.01 (s)	140.7, CH	8.90 (s)	150.4, CH	9.31 (s)
3	149.9, C		153.5, C		153.4, C	
4	127.6, C		122.4, C		127.0, C	
4a	120.3, C		121.9, C		141.5, C	
5	182.8, C		182.8, C		178.2, C	
6	99.6, CH	5.71 (s)	99.0, CH	5.62 (s)	100.8, CH	5.75 (s)
7	149.9 ^(c)^, C		149.9, C		148.9, C	
8	181.4, C		181.2, C		179.8, C	
8a	120.4, C		117.3, C		126.7, C	
9		7.83 (brs)		7.81 (brq, 5.2)		7.84 (brq, 5.1)
10	29.0, CH_3_	2.85 (d, 4.9)	28.9, CH_3_	2.83 (d, 5.2)	28.9, CH_3_	2.82 (d, 5.1)
1′		11.98 (brs)		11.88 (brs)	177.6, C	
1′a	144.7 ^(c)^, C					
2′	113.3, CH	7.79 (d, 7.9)	137.6, CH	7.93 (t, 3.05)	99.6, CH	5.77 (s)
3′	129.9, CH	7.61 (td, 7.9, 1.3)	102.7, CH	6.70 (dd, 3.05, 1.83)	152.2, C	
4′	120.6, CH	7.31 (td, 8.1, 1.4)			180.9, C	
5′	120.8, CH	8.23 (d, 8.1)				
5′a	120.1, C					
5′						8.04 (brq, 4.9)
6′					29.0, CH_3_	2.83 (brd, 4.9)

^(a)^ 300 MHz; ^(b)^ 600 MHz. See Supplementary Materials for NMR spectra. ^(c)^ Small signals; the assignment was confirmed by their HMBC correlations.

**Table 3:** In vitro cytotoxic activities of mansouramycins A (**5**), C (**4b**), E (**1a**), and F (**2a**).

Compound	Potency		Tumor Selectivity			
	Mean IC_50_ μM (μgmL^−1^)	Mean IC_70_ μM (μgmL^−1^)	Selectivity */Total	% Selectivity	Rating **	Internal Code
Mansouramycin A (**5**)	13.44 (2.902)	26.26 (5.671)	4/36	11%	++	MNSG078
Mansouramycin C (**4b**)	0.089 (0.022)	0.167 (0.041)	10/36	28%	+++	MNSG091
Mansouramycin E (**1a**)	23.10 (6.398)	33.95 (9.405)	0/18	0%	-	MNSG089
Mansouramycin F (**2a**)	7.92 (1.797)	15.19 ((3.449)	7/36	19%	++	MNSG090

* individual IC_70_ < 1/3 mean IC_70_; e.g., if mean IC_70_ = 2.1 μM the threshold for above average sensitivity was IC < 0.7 μM, ** - (% selective = < 4%); + (4% > %selective >= 10%); ++ (10% > %selective >= 20%); +++ (% selective > 20%).

## Data Availability

Further data are available on request from the corresponding author.

## Supplementary Materials

The following are available online at https://www.mdpi.com/article/10.3390/md19120715/s1, NMR spectra and other supplementary data.
