# Supplementary material for: Mansouramycins E–G, Cytotoxic Isoquinolinequinones from Marine Streptomycetes
Source: Mar Drugs. 2021 Dec 20;19(12):715. doi: 10.3390/md19120715 (PMC8707544; doi:10.3390/md19120715)
Supplement: Supplementary file 1 [file marinedrugs-19-00715-s001.zip › marinedrugs-1481785-supplementary.pdf]

Mansouramycins E-G, Cytotoxic Isoquinolinequinones from Marine Streptomycetes

Mohamed Shaaban[†,‡,§], Khaled A. Shaaban[†,§], Gerhard Kelter[⊥], Heinz Herbert Fiebig[⊥], and Hartmut Laatsch[†,*]

[†]University of Göttingen, Institute of Organic and Biomolecular Chemistry, Tammannstrasse 2, D-37077 Göttingen, Germany; mshaaba@gmail.com, khaled_shaaban@uky.edu, hlaatsc@gwdg.de
[‡] Chemistry of Natural Compounds Department, Pharmaceutical and Drug Industries Research Institute, National Research Centre, El-Behoos St. 33, Dokki-Cairo 12622, Egypt; mshaa-ba@gmail.com
[⊥]Oncotest GmbH, Am Flughafen 12-14, D-79108 Freiburg, Germany; Gerhard.Kelter@crl.com, fiebig@4hf.eu

*Correspondence: hlaatsc@gwdg.de; Tel.: + (551) 393211. Fax: +(551) 399660[§]

Table of Contents:

Structure Elucidation by means of COCON

Table S1: Experimental and SPARTAN-calculated ^{13}C NMR shifts of mansouramycin E (1a) and of further five isomers suggested by COCON on basis of 2D NMR data; shift values were sorted in ascending order........ 7

Table S2: Experimental and SPARTAN-calculated ^{13}C NMR shifts of mansouramycin F (**2a**) and 13 further isomers. Four of them (green formula numbers) were predicted by COCON; the best agreement with the experimental 2D NMR data of mansouramycin F was found for **2a**. 9

Table S3: Experimental and SPARTAN-calculated ^{13}C NMR shifts of mansouramycin G (**3a**) and of further five isomers suggested by COCON on basis of 2D NMR data; shift values were sorted in ascending order........ 11

Spectra

Figure S1: Chemical structures of isoquinolinequinones **1-6** produced by *Streptomyces* sp. B1848, and alternative structures **1b-3b**. 4

Figure S2: Potential quinonoid structures for mansouramycin E. The green formulas were predicted by COCON calculations (numbers in brackets behind the bold formula numbers are the ID numbers in the three different COCON result sets). The black isomers depicted as "not predicted" (e.g. **1d** , **1g**) were not fitting on the 2D NMR correlations and therefore not found in the COCON calculations.... 7

Figure S3: Isomeric structures suggested by COCON for mansouramycin F (green formulas). The other *p*-quinones were obtained by systematic variation of the COCON structures. Numbers near the atoms are ^{13}C NMR shifts obtained by DFT calculations (see also Table S2). 9

Figure S4: Alternative structures of mansouramycin G, calculated with COCON from 2D NMR data: red and blue arrows are pointing to crucial correlations; ^{4}J HMBC correlations are indicated by dashed black arrows. 10

Figure S5: (+)-ESI-MS spectrum of Mansouramycin E (**1a**) 12
Figure S6: EI-MS and HREI-MS spectra of Mansouramycin E (**1a**). 13
Figure S7: UV spectra of Mansouramycin E (**1a**) in methanol. 14
Figure S8: UV spectra of Mansouramycin E (**1a**) in methanol, neutral. 15
Figure S9: IR (KBr) spectra of Mansouramycin E (**1a**). 16
Figure S10: ^{1}H NMR (DMSO-d_6, 300 MHz) spectrum of Mansouramycin E (**1a**). 17
Figure S11: ^{13}C NMR (DMSO-d_6, 150 MHz) spectrum of Mansouramycin E (**1a**). 18
Figure S12: ^{1}H,^{1}H-COSY (DMSO-d_6, 600 MHz) spectrum of Mansouramycin E (**1a**). 19
Figure S13: HSQC (DMSO-d_6, 600 MHz) spectrum of Mansouramycin E (**1a**) 20
Figure S14: HMBC (DMSO-d_6, 600 MHz) spectrum of Mansouramycin E (**1a**). 21
Figure S15: HMBC (DMSO-d_6, 600 MHz) spectrum of Mansouramycin E (**1a**). 22
Figure S16: HMBC (DMSO-d_6, 600 MHz) spectrum of Mansouramycin E (**1a**). 23
Figure S17: EI-MS spectrum of Mansouramycin F (**2a**). 24
Figure S18: (+)-CI-MS spectrum of Mansouramycin F (**2a**). 25
Figure S19: (+)-HRESI-MS spectrum of Mansouramycin F (**2a**). 26
Figure S20: UV spectra of Mansouramycin F (**2a**) in methanol. 27
Figure S21: IR (KBr) of Mansouramycin F (**2a**). 28
Figure S22: ^{1}H NMR ($CDCl_3$, 300 MHz) spectrum of Mansouramycin F (**2a**). 29
Figure S23: ^{1}H NMR (DMSO-d_6, 300 MHz) spectrum of Mansouramycin F (**2a**). 30
Figure S24: ^{1}H NMR (DMSO-d_6, 600 MHz) spectrum of Mansouramycin F (**2a**). 31
Figure S25: ^{13}C NMR (DMSO-d_6, 150 MHz) spectrum of Mansouramycin F (**2a**). 32
Figure S26: ^{1}H,^{1}H-COSY (DMSO-d_6, 600 MHz) spectrum of Mansouramycin F (**2a**). 33
Figure S27: HSQC (DMSO-d_6, 600 MHz) spectrum of Mansouramycin F (**2a**). 34
Figure S28: HMBC (DMSO-d_6, 600 MHz) spectrum of Mansouramycin F (**2a**). 35
Figure S29: HMBC (DMSO-d_6, 600 MHz) spectrum of Mansouramycin F (**2a**). 36
Figure S30: HMBC (DMSO-d_6, 600 MHz) spectrum of Mansouramycin F (**2a**). 37
Figure S31: HMBC (DMSO-d_6, 600 MHz) spectrum of Mansouramycin F (**2a**). 38
Figure S32: (+)-ESI-MS spectrum of Mansouramycin G (**3a**). 39
Figure S33: (+)-HRESI-MS spectrum of Mansouramycin G (**3a**). 40
Figure S34: (+)-HRESI-MS spectrum of Mansouramycin G (**3a**). 41
Figure S35: UV spectra of Mansouramycin G (**3a**) in methanol. 42
Figure S36: IR (KBr) spectra of Mansouramycin G (**3a**). 43
Figure S37: ^{1}H NMR (DMSO-d_6, 300 MHz) spectrum of Mansouramycin G (**3a**). 44

Figure S38: ^{1}H NMR (CD_3OD, 300 MHz) spectrum of Mansouramycin G (**3a**)... 45
Figure S39: ^{1}H NMR ($CDCl_3$, 300 MHz) spectrum of Mansouramycin G (**3a**)... 46
Figure S40: ^{13}C NMR (DMSO-d_6, 150 MHz) spectrum of Mansouramycin G (**3a**)...................................... 47
Figure S41: ^{1}H,^{1}H-COSY (DMSO-d_6, 600 MHz) spectrum of Mansouramycin G (**3a**). 48
Figure S42: HSQC (DMSO-d_6, 600 MHz) spectrum of Mansouramycin G (**3a**) 49
Figure S43: HSQC (DMSO-d_6, 600 MHz) spectrum of Mansouramycin G (**3a**). 50
Figure S44: HMBC (DMSO-d_6, 600 MHz) spectrum of Mansouramycin G (**3a**). 51
Figure S45: HMBC (DMSO-d_6, 600 MHz) spectrum of Mansouramycin G (**3a**). 52
Figure S46: HMBC (DMSO-d_6, 600 MHz) spectrum of Mansouramycin G (**3a**). 53
Figure S47: HMBC (DMSO-d_6, 600 MHz) spectrum of Mansouramycin G (**3a**). 54

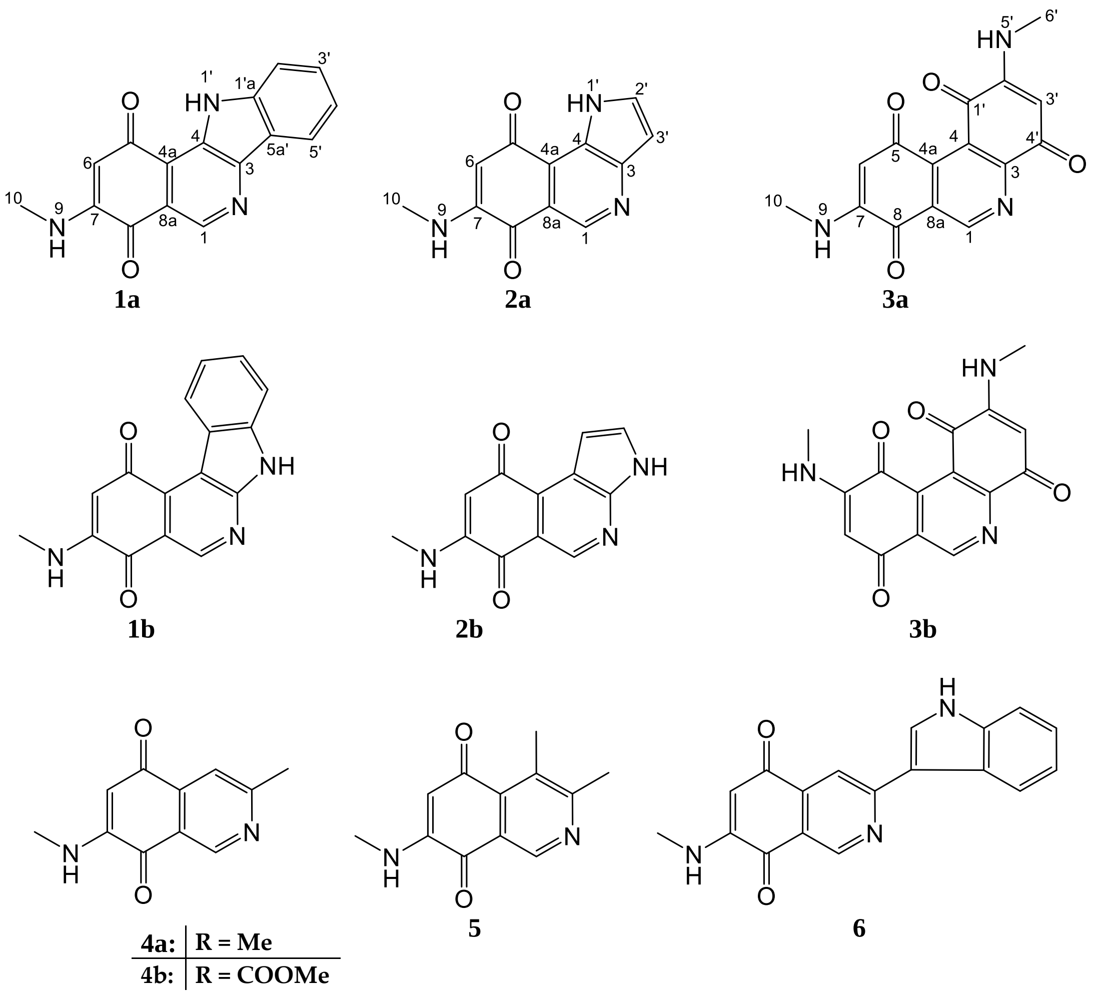

Figure S1: Chemical structures of isoquinolinequinones **1-6** produced by *Streptomyces* sp. B1848, and alternative structures **1b-3b**.

Structure elucidation by means of COCON

On the basis of ^{13}C NMR shifts and 2D NMR correlations (COSY and HMBC in our case), the computer program COCON [1] calculates all possible planar structures fitting on this data set. While a classical interpretation results usually in a very few alternatives or even a single structure, COCON delivers often an incredible high number of alternatives! For mansouramycin E, in first trial more than 204.000 (!) different constitutions with up to 8121 different shift assignments each were found. In total, > 740.000 candidates were calculated, where most of them, however, were highly strained (e.g. bridged aromatic systems, cyclobutenes, cyclobutadienes, non-linear allenes), or excluded because of less plausible functional groups (peroxides, nitroso groups, hydrazine derivatives).

One way to eliminate useless structures in COCON is to assign atom types (hybridisation and number of attached hydrogens), which can be derived mostly from NMR shifts and integrals. For mansouramycin E, this reduced the number of hits to still 24093 candidates. When all identical structures with permutating

assignments of similar shifts were removed, then 7603 constitutions were left; some of the quinonoid structures are depicted in Figure S1. A further way to reduce the number of results is to define bonds between clearly assigned atoms. It is plausible e.g., that the two carbon atoms in mansouramycin E at δ ~180 ppm are connected with oxygen forming carbonyl groups. It is also derived for sure from COSY and HMBC correlations that an *o*-disubstituted benzene ring (or even an indole unit) is present. With these restrictions, only the two structures **1a** and **1c** remained; the first one is having the N-methyl group at C-7 as in mansouramycin A, whose structure was proven by synthesis. To confirm this selection, the ^{13}C NMR shifts were calculated for all *o*- and *p*-quinonoid isomers with a quinoline or isoquinoline skeleton (Figure S1) by means of *ab initio* methods[2] and compared with the experimental data (Table S1):

Mansouramycin E

For mansouramycin E, structure **1a** gave the best agreement between experimental and calculated ^{13}C NMR shifts; an *o*-quinone should show shift differences of ~ 10 ppm between the carbonyl groups and was less plausible.

It should be stated that the COCON results derived from HMBC and COSY correlations did not contain structures **1i**, **1j**, **1q**, or any other formula with the ring nitrogen atoms in a formal *cis*-orientation.

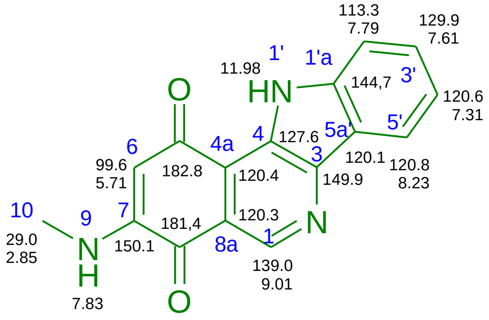

1a with experimental shifts

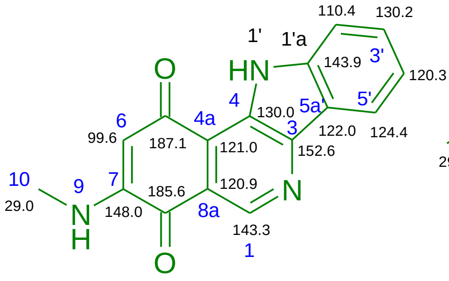

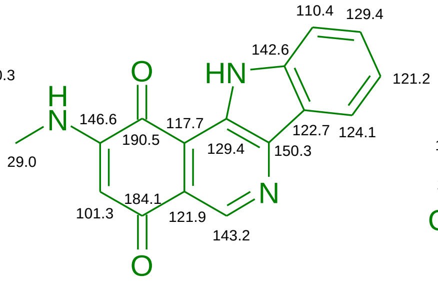

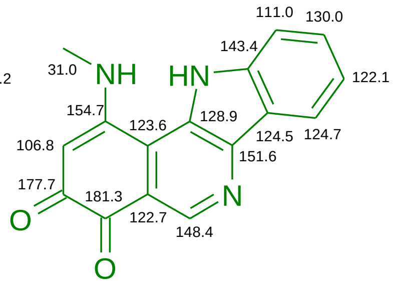

1a (7, 22, 653) **1b** (9, 26, 704) **1c** (17, 41, 1164)

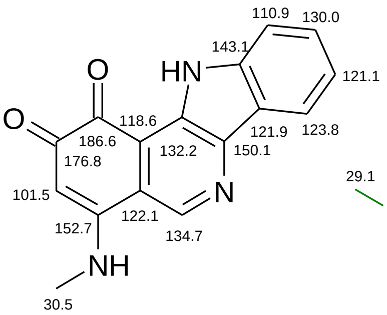

1d (not predicted)

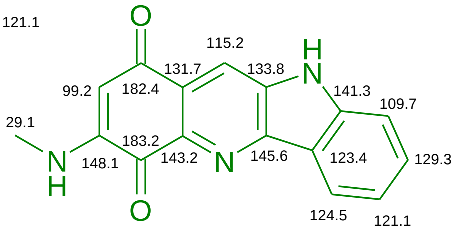

1e (84, 144, 3032)

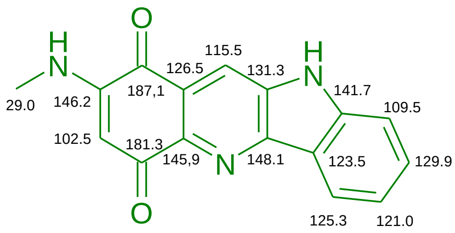

1f (77, 131, 2852)

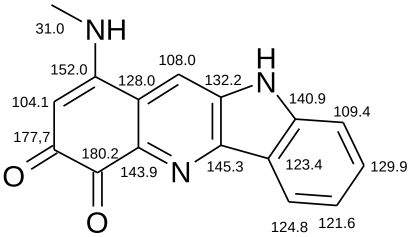

1g (not predicted)

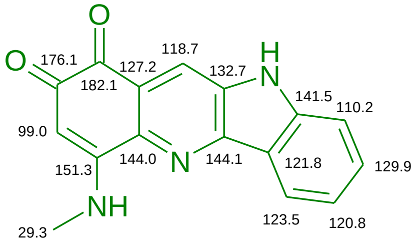

1h (56,103, 2779)

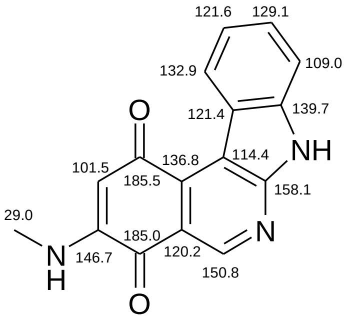

1i (not predicted)

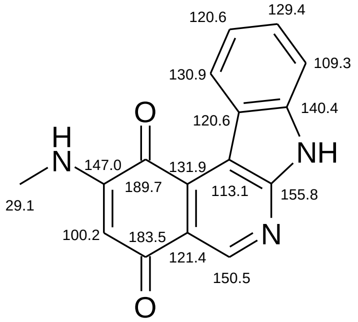

1j (not predicted)

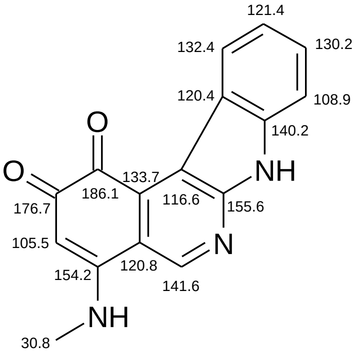

1k (not predicted)

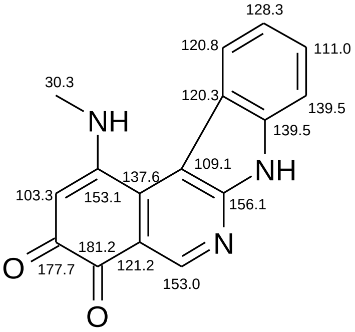

1L (not predicted)

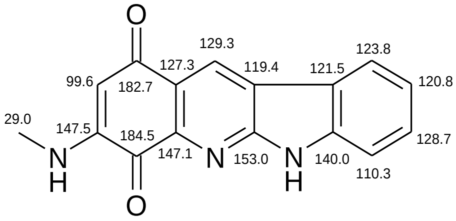

1m (not predicted)

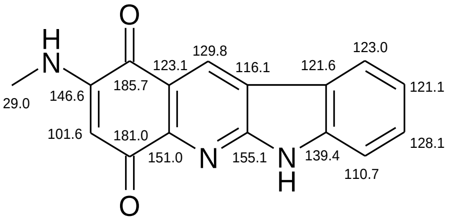

1n (not predicted)

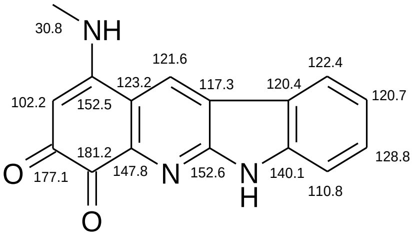

1o (not predicted)

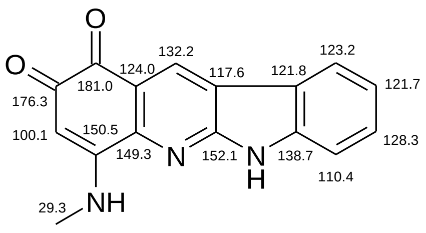

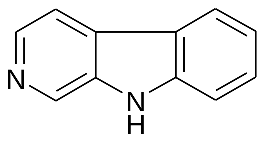

1p (not predicted) **1q** (not a predicted substruct.)

Figure S2: Potential quinonoid structures for mansouramycin E. The green formulas were predicted by COCON calculations (numbers in brackets behind the bold formula numbers are the ID numbers in the three different COCON result sets). The black isomers depicted as "not predicted" (e.g. **1d** , **1g**) were not fitting on the 2D NMR correlations and therefore not found in the COCON calculations.

Table S1: Experimental and SPARTAN-calculated ^{13}C NMR shifts of mansouramycin E (**1a**) and of further five isomers suggested by COCON on basis of 2D NMR data; shift values were sorted in ascending order.

	exp	1a	1b	1c	1e	1f	1h
10	29.0	29.0	29.0	31.0	29.1	29.0	29.3
6	99.6	99.6	101.3	106.8	99.2	102.5	99.0
2'	113.3	110.4	110.4	111.0	109.7	109.5	110.2
5'a	120.1	120.3	117.7	122.1	115.2	115.5	118.7
4a	120.4	120.9	121.2	122.7	121.1	121.1	120.8
8a	120.3	121.0	121.9	123.6	123.4	123.5	121.8
4'	120.6	122.0	122.7	124.5	124.5	125.3	123.5
5'	120.8	124.4	124.1	124.7	129.3	126.5	127.2
4	127.6	130.0	129.4	128.9	131.7	129.9	129.9
3'	129.9	130.2	129.4	130.0	133.8	131.3	132.7
1	139.0	143.3	142.6	143.4	141.3	141.7	141.5
1'a	144.7	143.9	143.2	148.4	143.2	145.9	144.0
3	149.9	148.0	146.6	151.6	145.6	146.2	144.1
7	150.1	152.6	150.3	154.7	148.1	148.1	151.3
8	181.4	185.6	184.1	177.7	182.4	181.3	176.1
5	182.8	187.1	190.5	181.3	183.2	187.1	182.1
$\Sigma = \lvert\delta_{exp}-\delta_{calcd}\rvert$		**30.0**	**36.1**	**47.9**	**44.7**	**43.3**	**37.9**

Mansouramycin F

The structure of mansouramycin F was determined in the same way as for **1a** by means of COCON. Interpretation of the 2D NMR data (including NH/C HMBC correlations in this case) with fixed carbonyl groups but *without* using atom types gave 240 alternatives. *With* atom types, the calculation predicted only 19 alternatives, among them the four *p*-quinones **2a-2d** (green structures in Table S2) The other 15 compounds were *cyclo*-heptatrienones or highly strained bridged ring systems; *o*-quinones were not found by COCON, also not in the calculation without atom types.

To assign the correct structure, experimental and calculated ^{13}C NMR shifts of the predicted quinones were compared as for **1a**. For a further confirmation, ten additional *p*-quinones were included into the data set (Figure S2), but did not change the final result (Table S2) that **2a** is the correct structure of mansouramycin F.

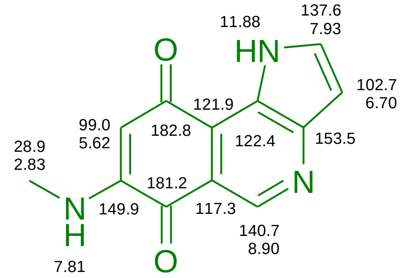

2a with experimental shifts

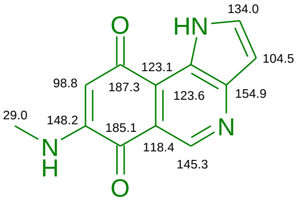

2a

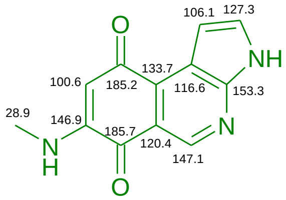

2b

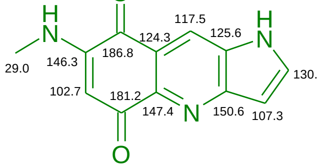

2c

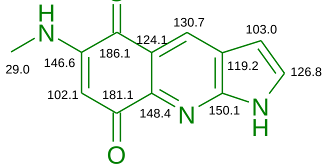

2d

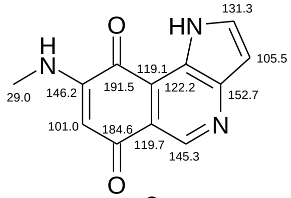

2e

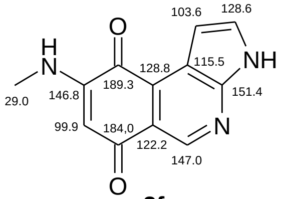

2f

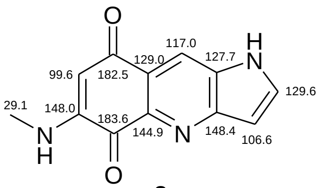

2g

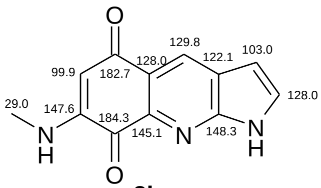

2h

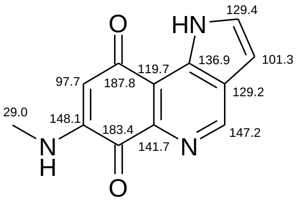

2i

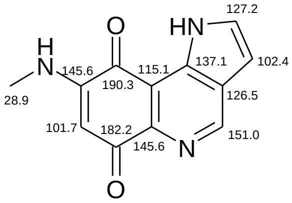

2j

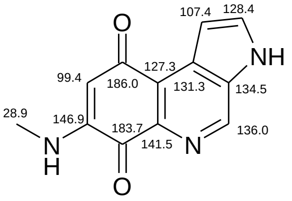

2k

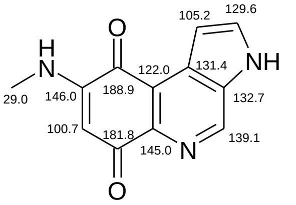

2L

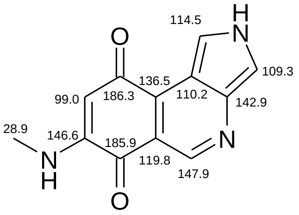

2m

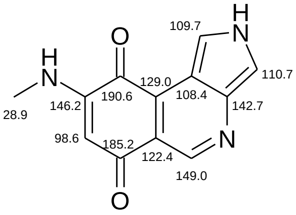

2n

Figure S3: Isomeric structures suggested by COCON for mansouramycin F (green formulas). The other *p*-quinones were obtained by systematic variation of the COCON structures. Numbers near the atoms are ^{13}C NMR shifts obtained by DFT calculations (see also Table S2).

Table S2: Experimental and SPARTAN-calculated ^{13}C NMR shifts of mansouramycin F (**2a**) and 13 further isomers. Four of them (green formula numbers) were predicted by COCON; the best agreement with the experimental 2D NMR data of mansouramycin F was found for **2a**.

	exp	2a	2b	2c	2d	2e	2f	2g	2h	2i	2j	2k	2l	2m	2n
Me	28.9	29.0	28.9	29.0	29.0	29.0	29.0	29.1	29.0	29.0	28.9	28.9	29.0	28.9	28.9
6	99.0	98.8	100.6	102.7	102.1	101.0	99.9	99.6	99.9	97.7	101.7	99.4	100.7	99.0	98.6
3'	102.7	104.5	106.1	107.3	103.0	105.5	103.6	106.6	103.0	101.3	102.4	107.4	105.2	109.3	108.4
8a	117.3	118.4	116.6	117.5	119.2	119.1	115.5	117.0	122.1	119.7	115.1	127.3	122.0	110.2	109.7
4a	121.9	123.1	120.4	124.3	124.1	119.7	122.2	127.7	128.0	129.2	126.5	128.4	129.6	114.5	110.7
4	122.4	123.6	127.3	125.6	126.8	122.2	128.6	129.0	128.0	129.4	127.2	131.3	131.4	119.8	122.4
2'	137.6	134.0	133.7	130.7	130.7	131.3	128.8	129.6	129.8	136.9	137.1	134.5	132.7	136.5	129.0
1	140.7	145.3	146.9	146.3	146.6	145.3	146.8	144.9	145.1	141.7	145.6	136.0	139.1	142.9	142.7
7	149.9	148.2	147.1	147.4	148.4	146.2	147.0	148.0	147.6	147.2	145.6	141.5	145.0	146.6	146.2
3	153.5	154.9	153.3	150.6	150.1	152.7	151.4	148.4	148.3	148.1	151.0	146.9	146.0	147.9	149.0
8	181.2	185.1	185.2	181.2	181.1	184.6	184.0	182.5	182.7	183.4	182.2	183.7	181.8	185.9	185.2
5	182.8	187.3	185.7	186.8	186.1	191.5	189.3	183.6	184.3	187.8	190.3	186.0	188.9	186.3	190.6
$\Sigma = \|\delta_{exp}-\delta_{calcd}\|$		**25.3**	32.1	36.1	33.1	36.6	39.4	38.7	40.5	36.5	35.3	59.0	51.3	44.1	55.5

The black structures **2e-2n** in Figure S3 did not agree with the experimental 2D NMR correlations of mansouramycin F: In the experimental HMBC spectrum, both the NH proton of the aminomethyl group (δ 7.81) and the low-field methine of the pyridine ring (δ 8.90) correlated with the same carbonyl group (δ 181.2). Such a correlation would be expected for **2a-2d**, but not for the other isomers (with exception of **2m**, which is omitted, however, because of the wrong pyrrole methine shifts). Additionally, the deviation between experimental and calculated shifts is rather high: **2e-2n** and were therefore excluded from further discussions.

The shift value predicted for the pyridine CH in **2c** (δ 117.5) is definitely out of the confidence limit of the DFT calculations, which excluded this isomer as well. The methine signal of the pyridine ring at δ 8.90 did not show an HMBC correlation with C-3' and *vice versa*, so that also **2d** was sorted out, and only **2a** and **2b** were remaining. In **2b**, H-3' and H-6 should both couple with C-4a, which is, however, not seen. Amongst all compounds in Table S2, the experimental ^{13}C NMR shifts are fitting best on the values calculated for **2a** (Figure S3). We assumed therefore this structure for mansouramycin F.

Mansouramycin G

Analysis of the mansouramycin F data was performed in the same way as described before. Predicted by COCON calculations with given atom types were 825 constitutions, among them 72 quinonoid isomers. For a fine-tuning and to speed-up the calculations, two vinyl-aminomethyl (CH=C-NHMe) fragments were defined by fixed atom connections. Additionally, the weak HMBC cross signals between NH-9 (δ 7.84) and CO-5 (δ 178.2) and correspondingly NH-5' (δ

8.04) and CO-1' (δ 177.6) were supposed to be due to ^4J correlations. This reduced the number of hits from 825 to only 15 isomers, namely 6 isoquinolinquinones (Figure S4), two isoindolequinone derivatives, and eight unsaturated tetrones without structural relations with the mansouramycins.

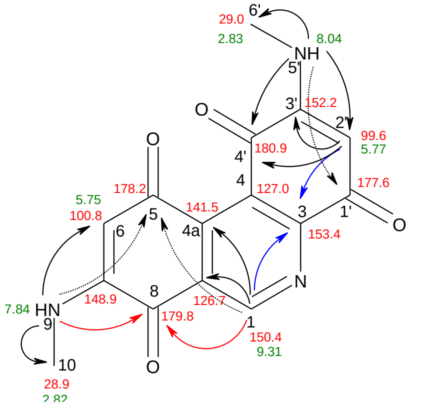

3a with experimental shifts

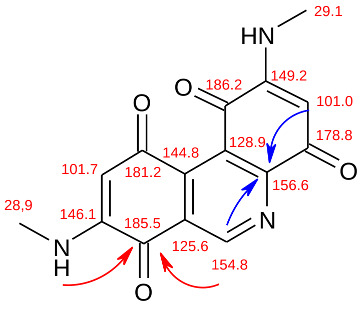

3a (G20)

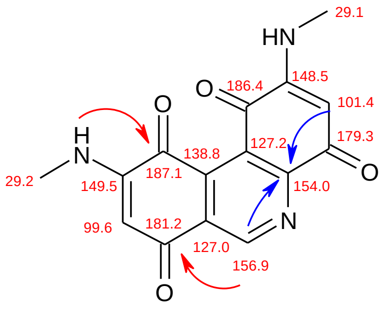

3b (G21)

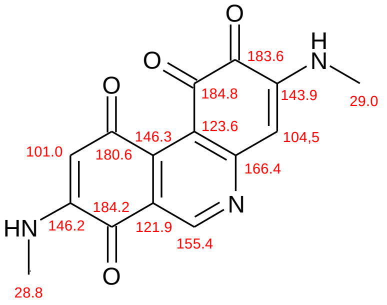

3c (G28)

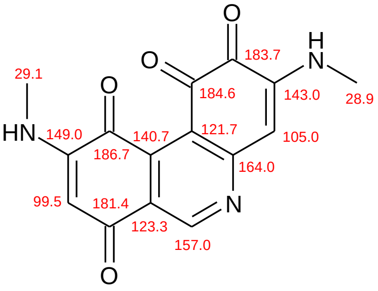

3d (G27)

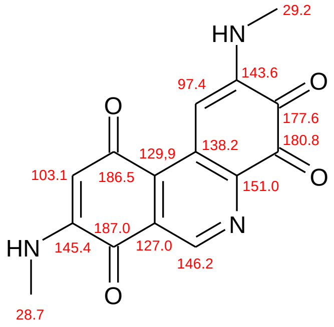

3e (G30)

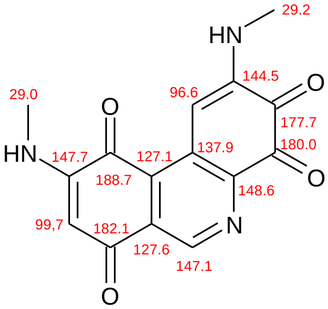

3f (G29)

Figure S4: Alternative structures of mansouramycin G, calculated with COCON from 2D NMR data: red and blue arrows are pointing to crucial correlations; ^4J HMBC correlations are indicated by dashed black arrows.

NH-9 in mansouramycin G showed strong HMBC signals with C-6, C-8, C-10 and coupled weakly (^4J) with C-5. CH-1 coupled strongly with the carbonyl C-8 and with C-3, which is also

seen by H-2'. It follows that a similar coupling pattern of C-8 should happen only with the aminomethyl group at C-7, i.e. only in the structures **3a**, **3c**, and **3e**. Comparison of their calculated ^{13}C NMR shifts with the experimental data gave the best correlation for **3a**. We are assuming therefore that mansouramycin G is having structure **3a**.

Table S3: Experimental and SPARTAN-calculated ^{13}C NMR shifts of mansouramycin G (**3a**) and of further five isomers suggested by COCON on basis of 2D NMR data; shift values were sorted in ascending order.

	exp	3a	3c	3e	3b	3d	3f
10	28.9	28.9	28.8	28.7	29.1	28.9	29.0
6	29.0	29.1	29.0	29.2	29.2	29.1	29.2
2'	99.6	101.0	101.0	97.4	99.6	99.5	96.6
5'a	100.8	101.7	104.5	103.1	101.4	105.0	99.7
4a	126.7	125.6	121.9	127.0	127.0	121.7	127.1
8a	127.0	128.9	123.6	129.9	127.2	123.3	127.6
4'	141.5	144.8	143.9	138.2	138.8	140.7	137.9
5'	148.9	146.1	146.2	143.6	148.5	143.0	144.5
4	150.4	149.2	146.3	145.4	149.5	149.0	147.1
3'	152.2	154.8	155.4	146.2	154.0	157.0	147.7
1	153.4	156.6	166.4	151.0	156.9	164.0	148.6
1'a	177.6	178.8	180.6	177.6	179.3	181.4	177.7
3	178.2	181.2	183.6	180.8	181.2	183.7	180.0
7	179.8	185.5	184.2	186.5	186.4	184.6	182.1
8	180.8	186.2	184.8	187.0	187.1	186.7	188.7
$\Sigma = \lvert\delta_{exp}-\delta_{calcd}\rvert$		**33.8**	**55.6**	**45.6**	**28.4**	**56.6**	**38.1**

---

[1] **Lindel, T.; Junker J.; Koeck, M. 2D-NMR-guided constitutional analysis of organic compounds employing the computer program COCON.** *Eur. J. Org. Chem.* 1999**, 573–577.**

[2] SPARTAN'20, Wavefunction, Inc.: Irvine, CA, USA, **2020**.SPARTAN

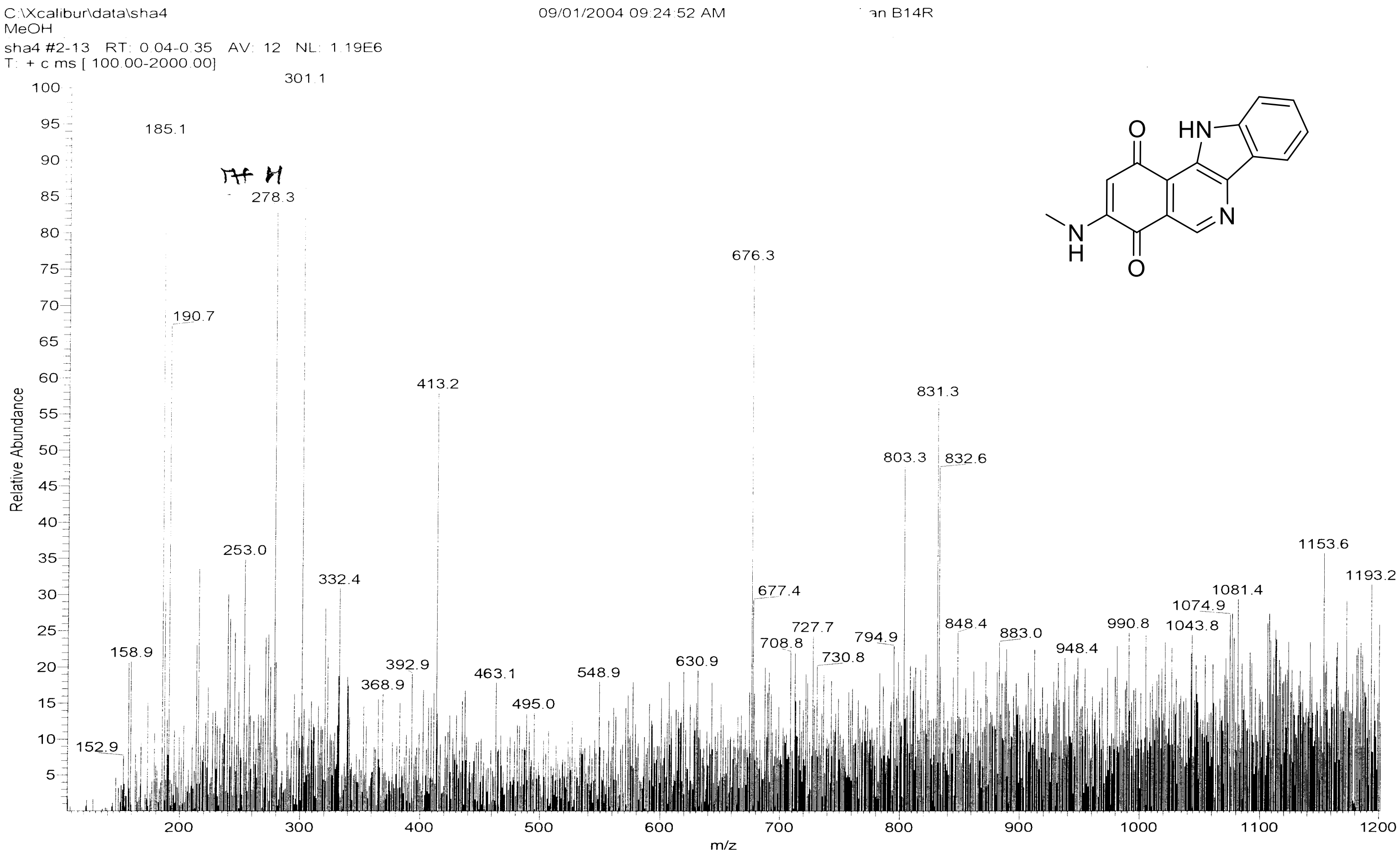

Figure S5: (+)-ESI-MS spectrum of Mansouramycin E (**1a**)

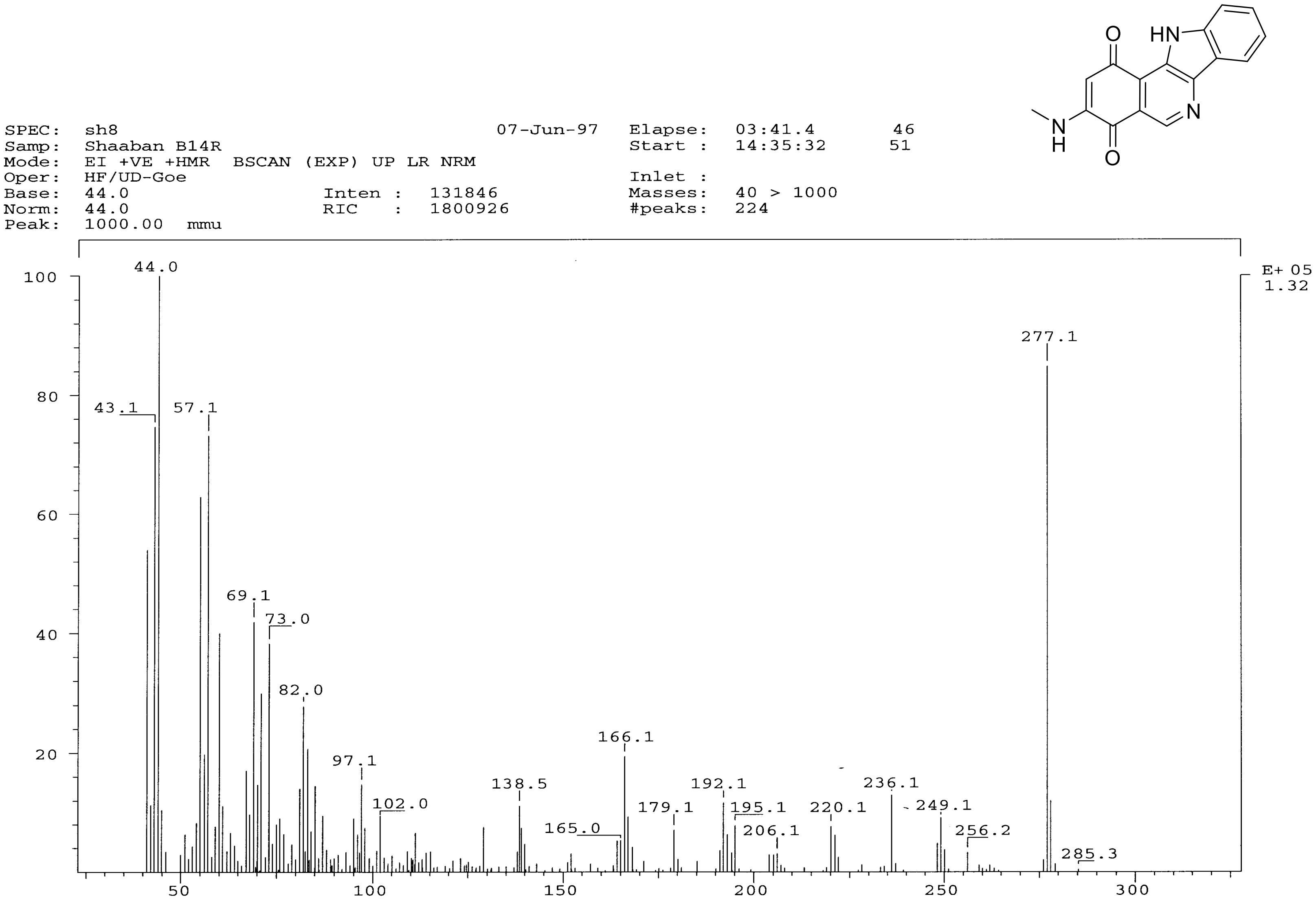

Figure S6: EI-MS and HREI-MS spectra of Mansouramycin E (**1a**).

11.11.04 16:17:01

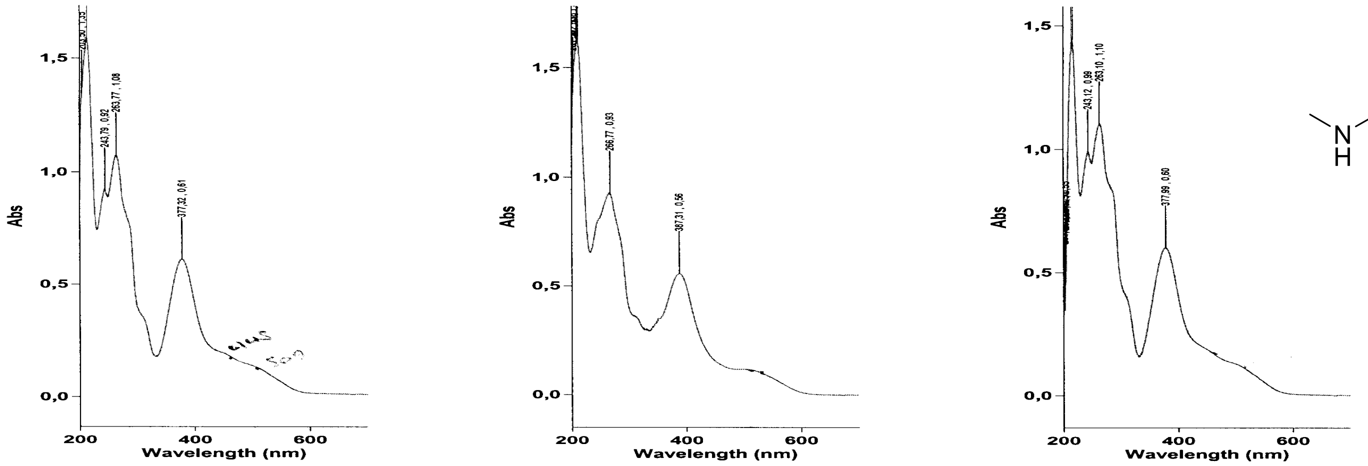

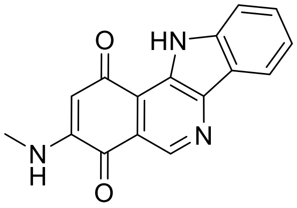

Scan Analysis Report

Report Time : Do 11 Nov 03:36:25 PM 2004
Batch: C:\UV-Daten\Laatsch\B14R.bsw
Software version: 02.00(25)
Operator:

Instrument Parameters

Instrument	Cary 300
Instrument Version	9,00
Start (nm)	700,00
Stop (nm)	200,00
X Mode	Nanometers
Y Mode	Abs
UV-Vis Scan Rate (nm/min)	199,800
UV-Vis Data Interval (nm)	0,333
UV-Vis Ave. Time (sec)	0,100
UV-Vis SBW (nm)	2,0
Beam Mode	Double
Signal-to-noise Mode	Off
UV Source	On
Vis Source	On
Source Changeover (nm)	350,00
Baseline Correction	Off
Cycle Mode	Off
Accessory	Cell changer
Comments	B14R<br>c=0.189 mg/10.0 mL Methanol

Sample Name: neutral

Wavelength (nm)	Abs
377,32	0,6102
263,77	1,0771
243,79	0,9216
212,49	1,5934
203,50	1,3490

Sample Name: acidic

Collection Time

Peak Table
Peak Style
Peak Threshold
Range

Wavelength (nm)	Abs
387,31	0,5572
266,77	0,9273
210,16	1,6281
208,49	1,6356
207,83	1,6340
202,50	1,4448
201,50	1,3823

Sample Name: basic

Collection Time

Peak Table
Peak Style
Peak Threshold
Range

Wavelength (nm)	Abs
377,99	0,6002
263,10	1,1028
243,12	0,9906
215,82	1,4476
213,49	1,4301
203,50	0,5453
202,83	0,5024
201,50	0,4431
200,50	0,4551

Figure S7: UV spectra of Mansouramycin E (**1a**) in methanol.

11.11.04 16:18:56

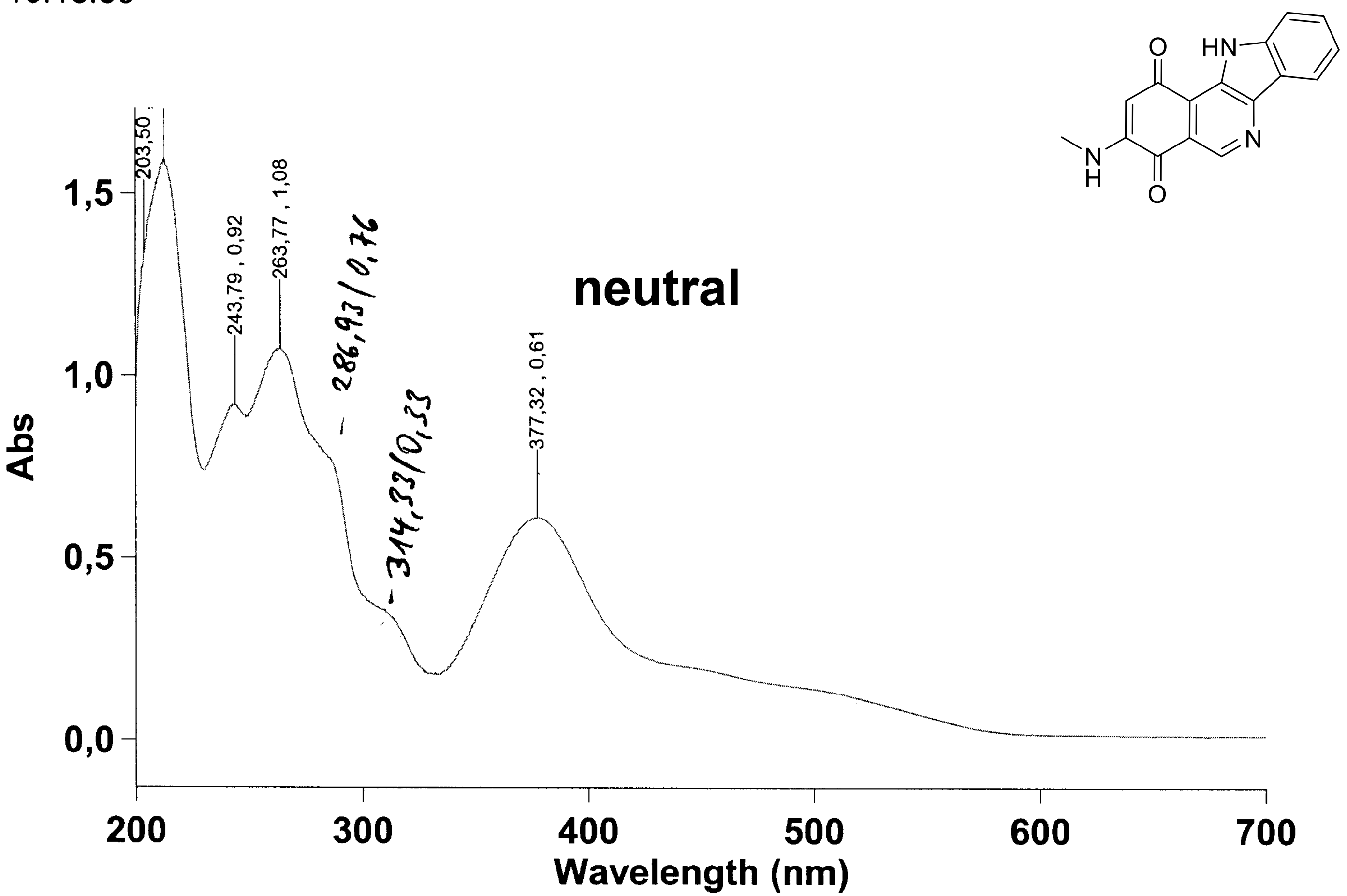

Figure S8: UV spectra of Mansouramycin E (**1a**) in methanol, neutral.

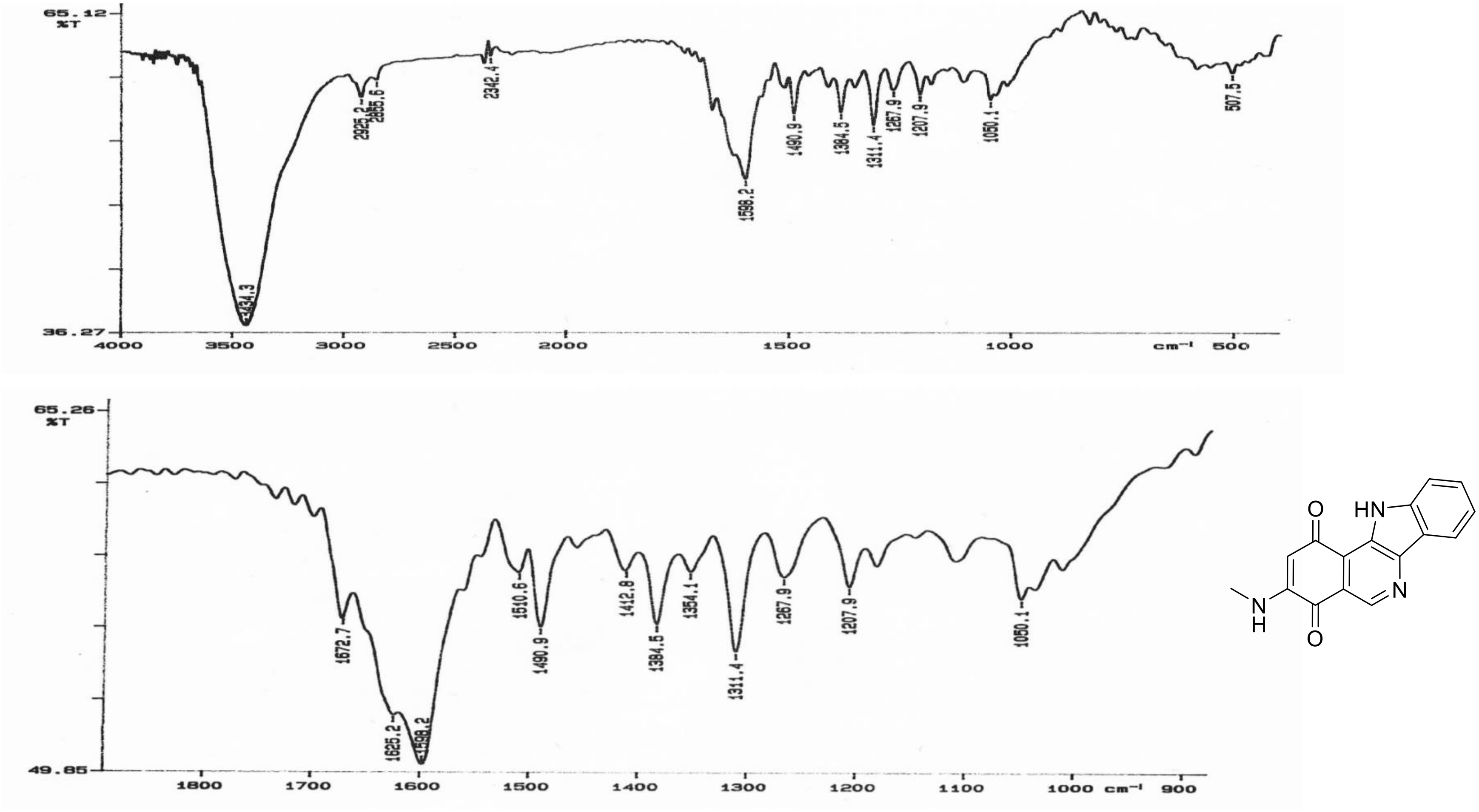

Figure S9: IR (KBr) spectrum of Mansouramycin E (**1a**).

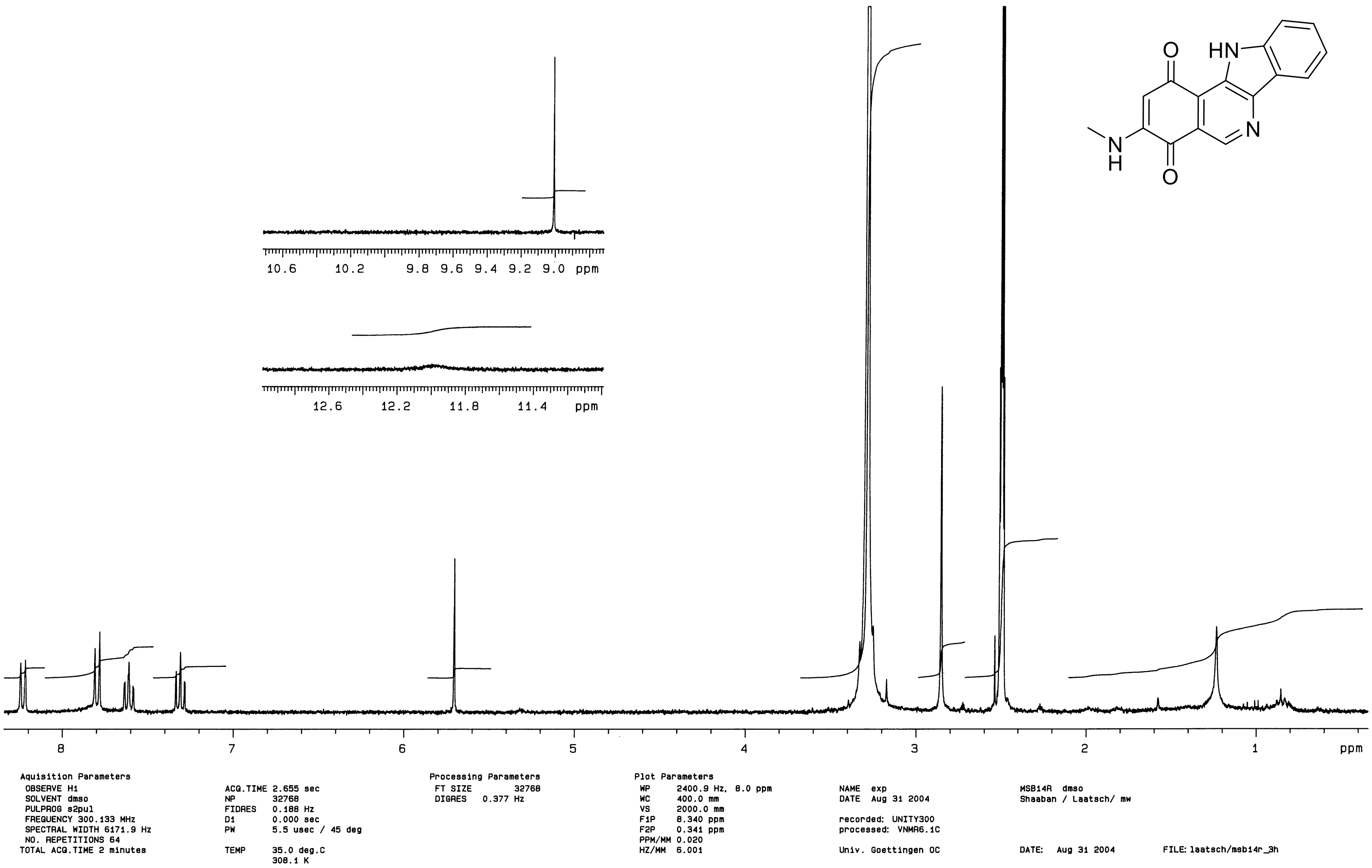

Figure S10: ^{1}H NMR (DMSO-d_6, 300 MHz) spectrum of Mansouramycin E (**1a**).

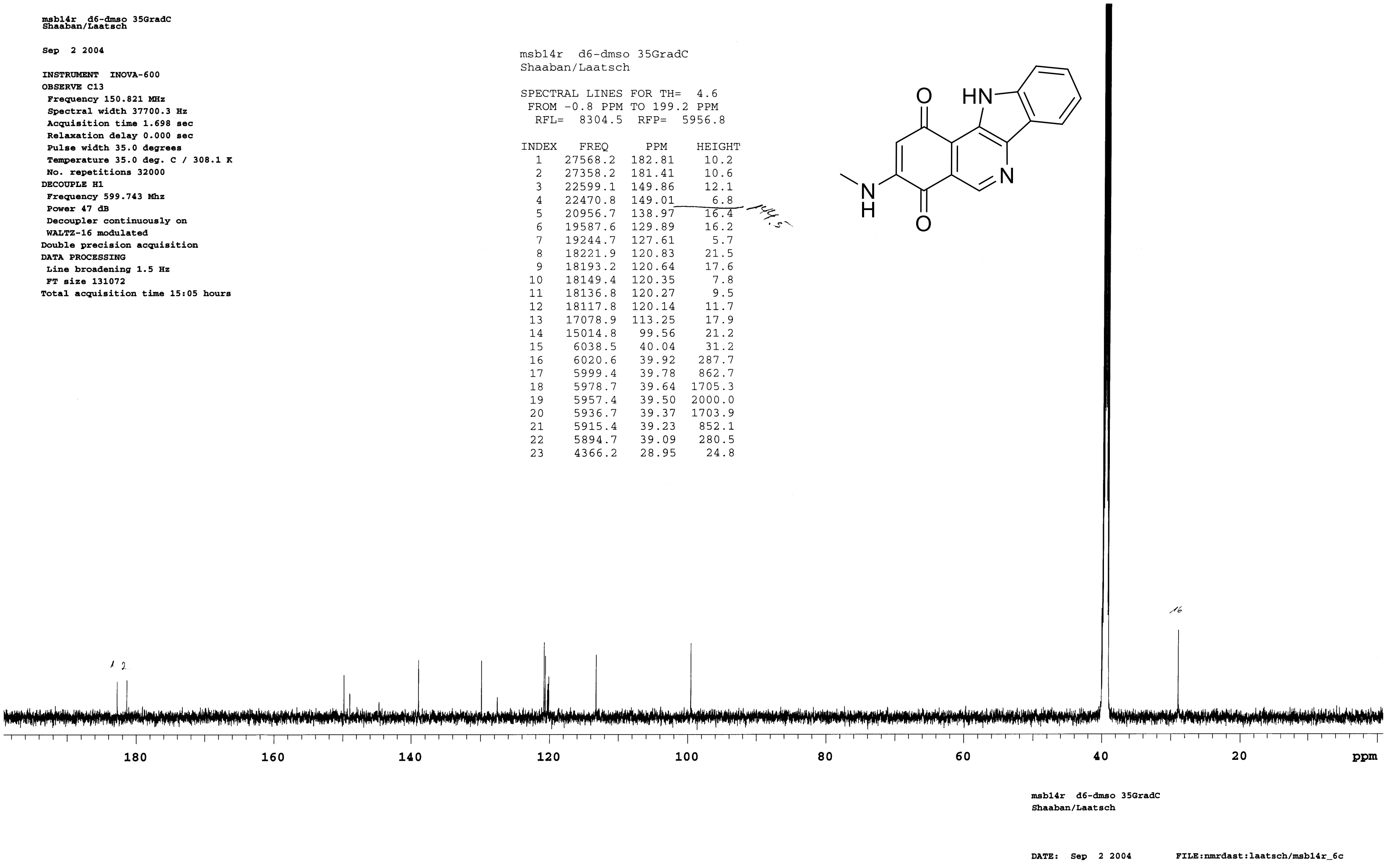

Figure S11: ^{13}C NMR (DMSO-d_6, 150 MHz) spectrum of Mansouramycin E (**1a**).

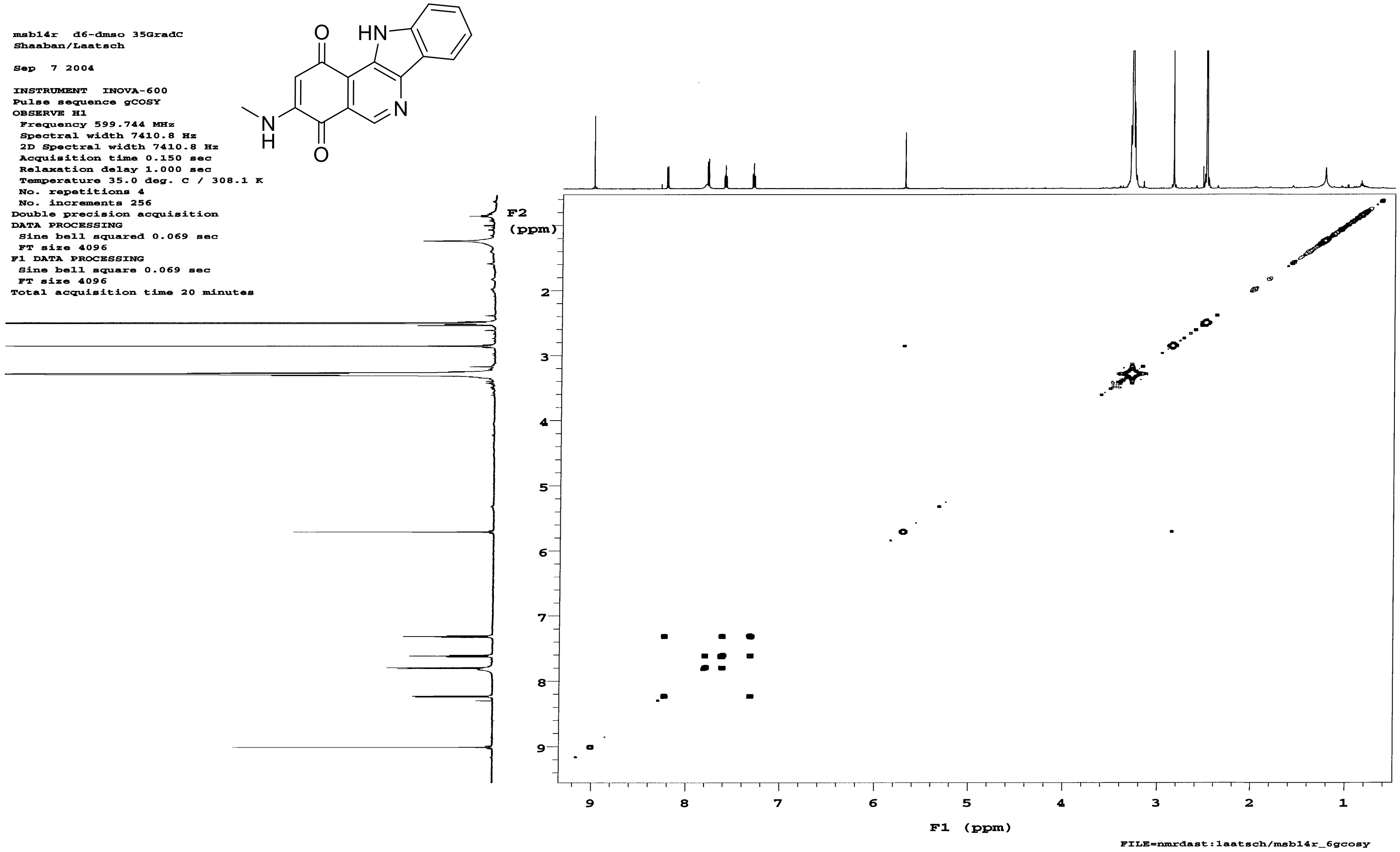

Figure S12: $^{1}H,^{1}H$-COSY (DMSO-d_6, 600 MHz) spectrum of Mansouramycin E (**1a**).

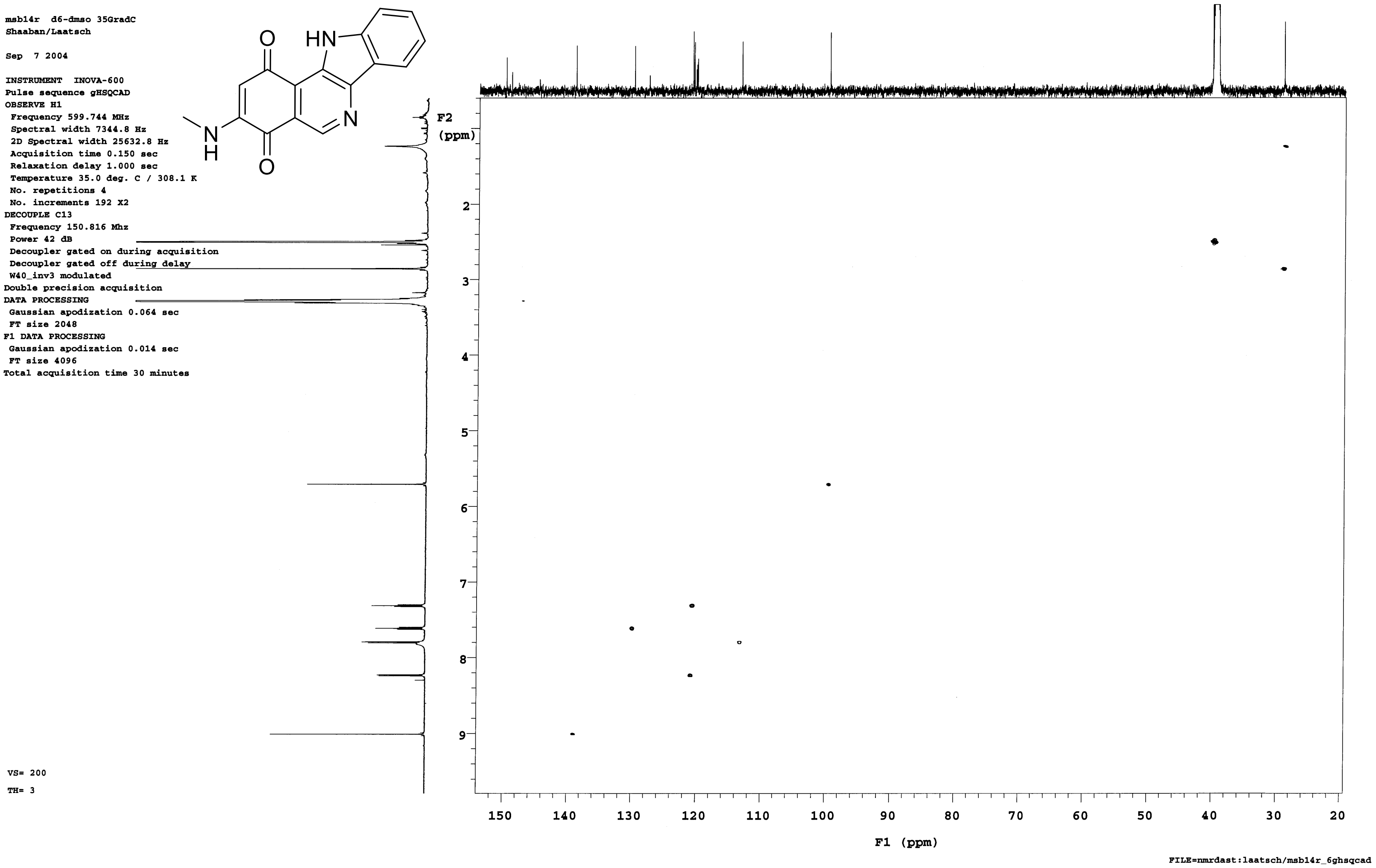

Figure S13: HSQC (DMSO-d_6, 600 MHz) spectrum of Mansouramycin E (**1a**)

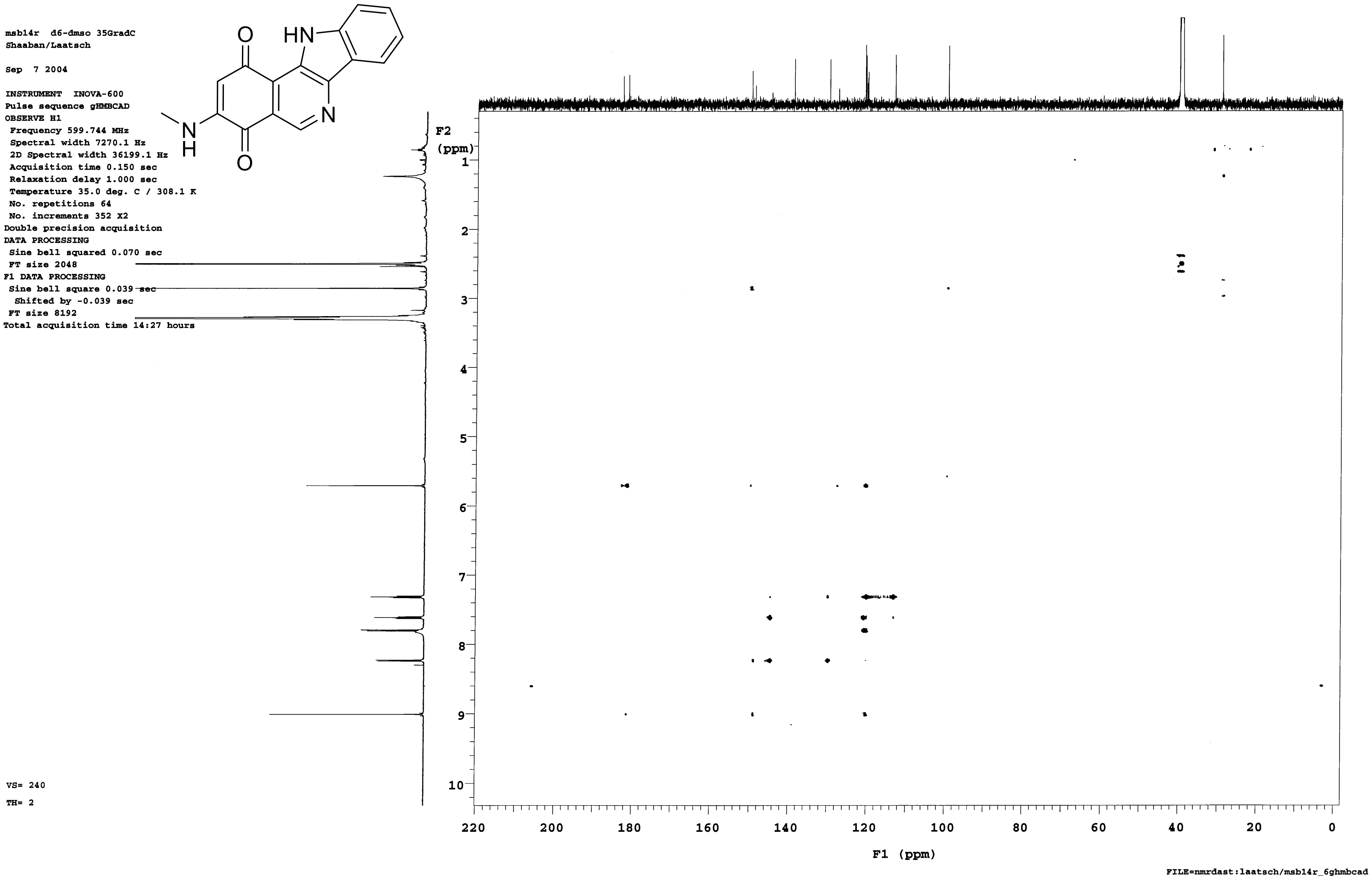

Figure S14: HMBC (DMSO-d_6, 600 MHz) spectrum of Mansouramycin E (**1a**).

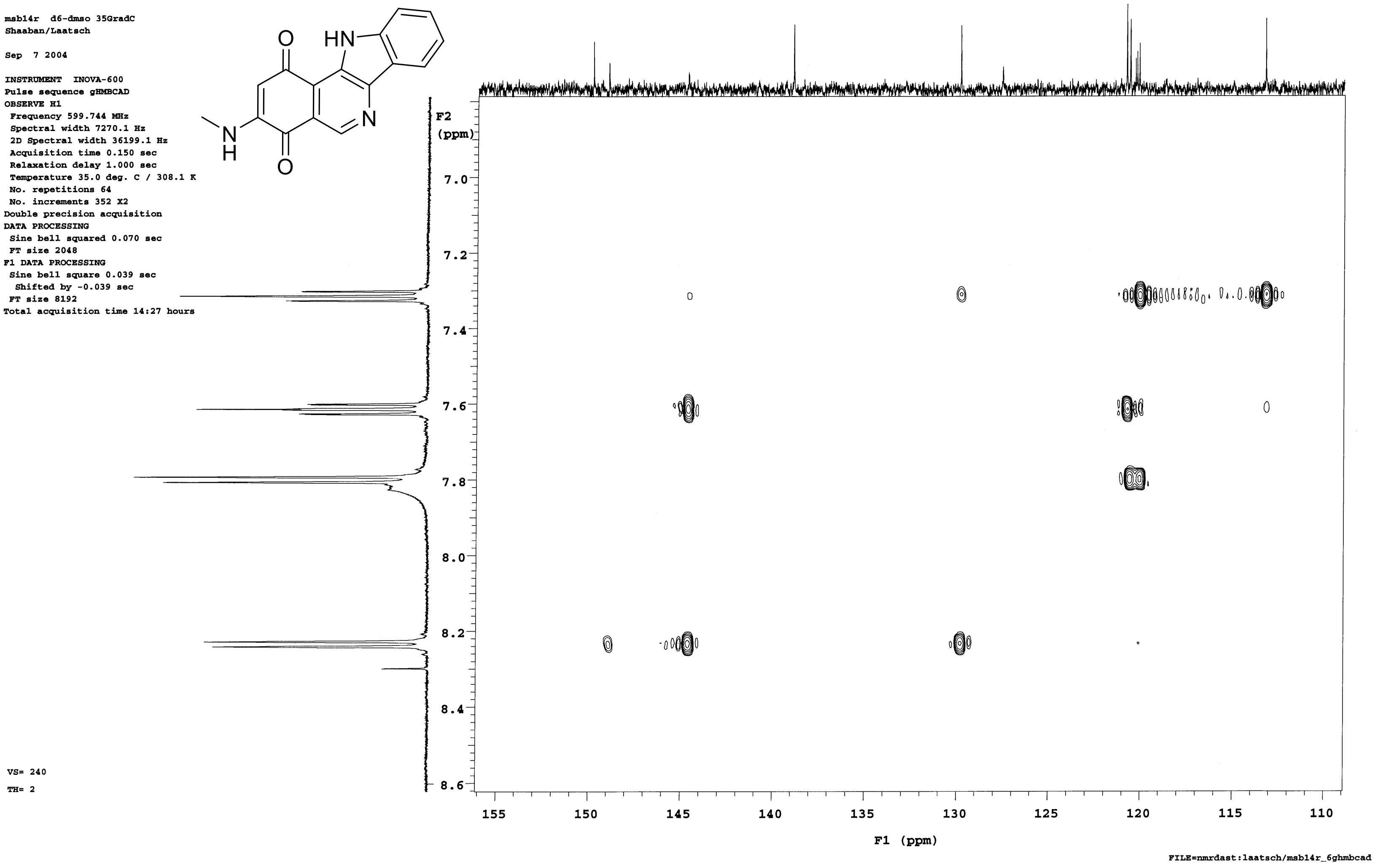

Figure S15: HMBC (DMSO-d_6, 600 MHz) spectrum of Mansouramycin E (**1a**).

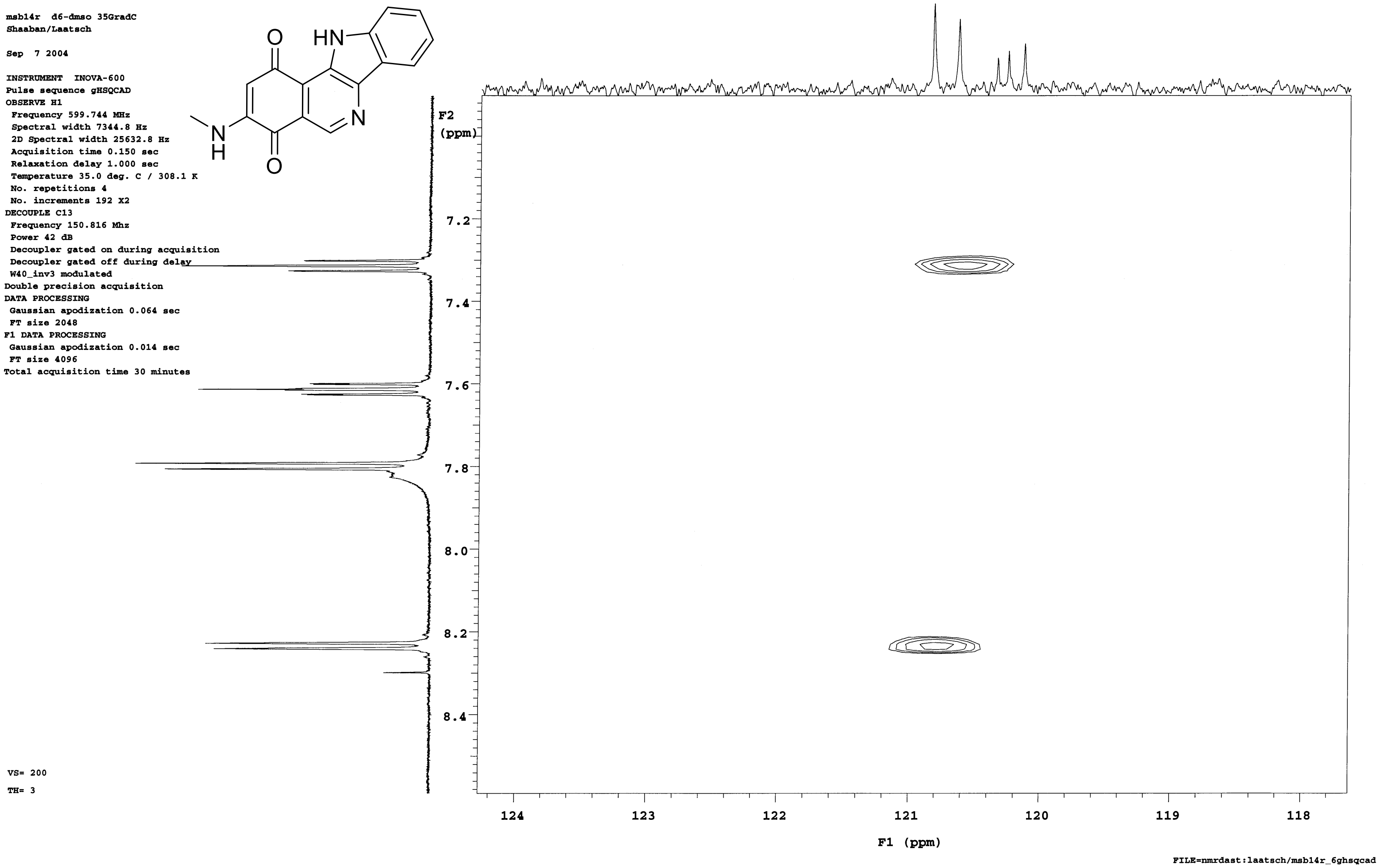

Figure S16: HMBC (DMSO-d_6, 600 MHz) spectrum of Mansouramycin E (**1a**).

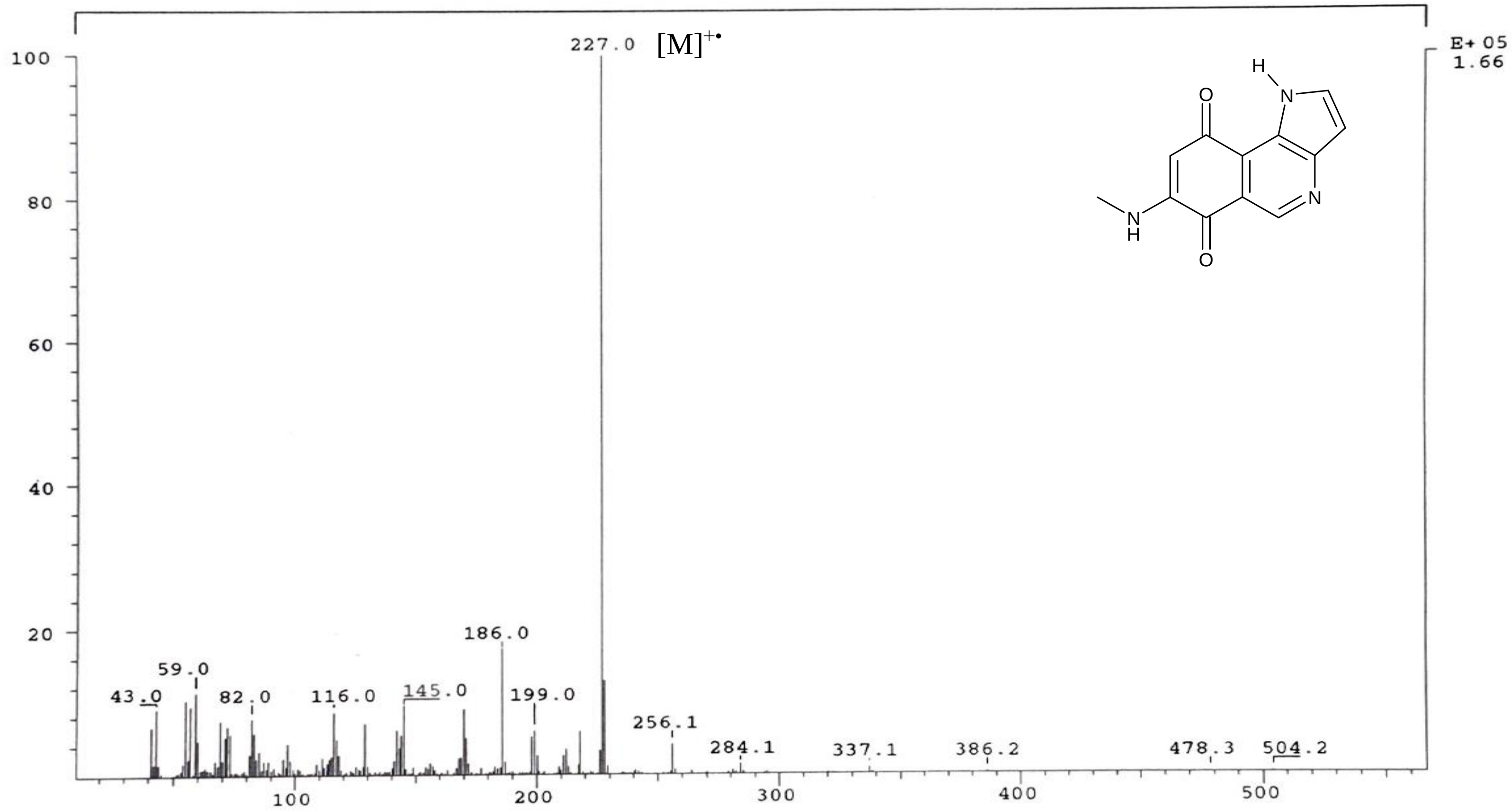

Figure S17: EI-MS spectrum of Mansouramycin F (**2a**).

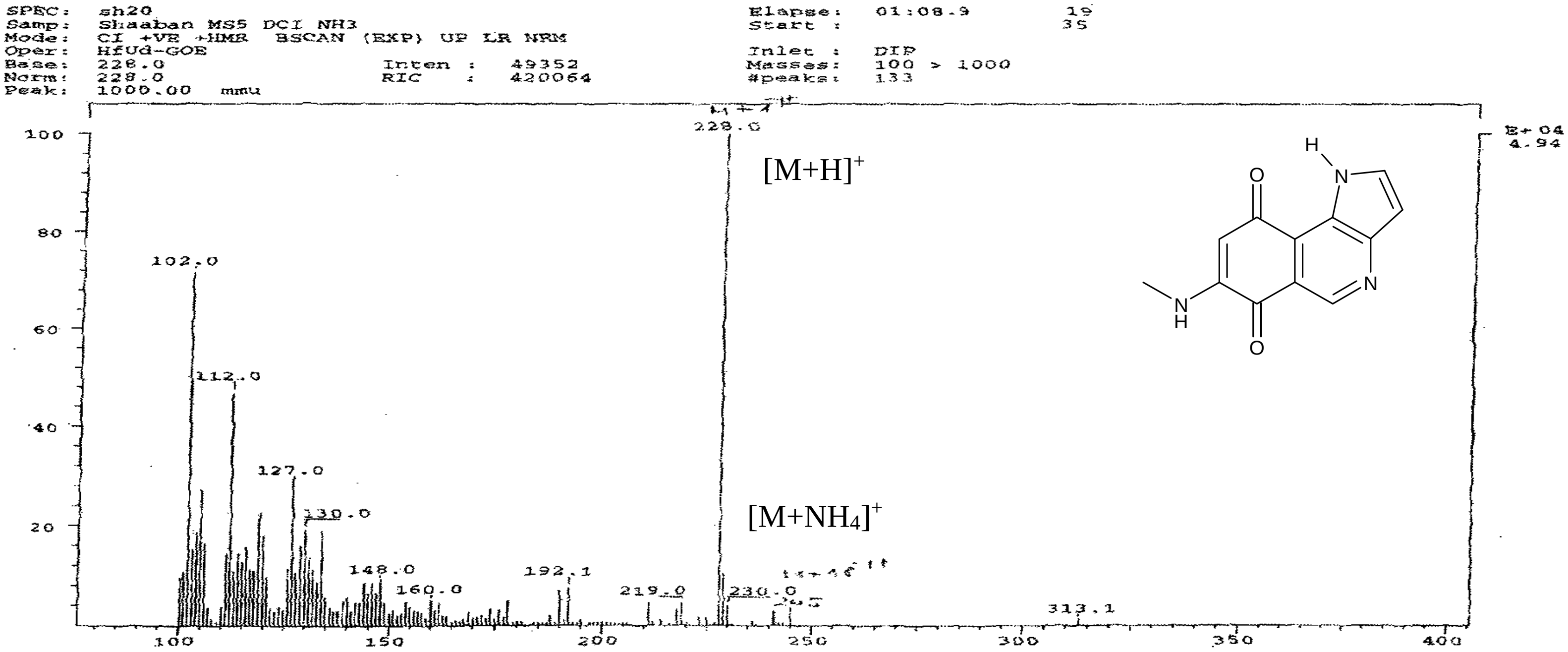

Figure S18: (+)-CI-MS spectrum of Mansouramycin F (**2a**).

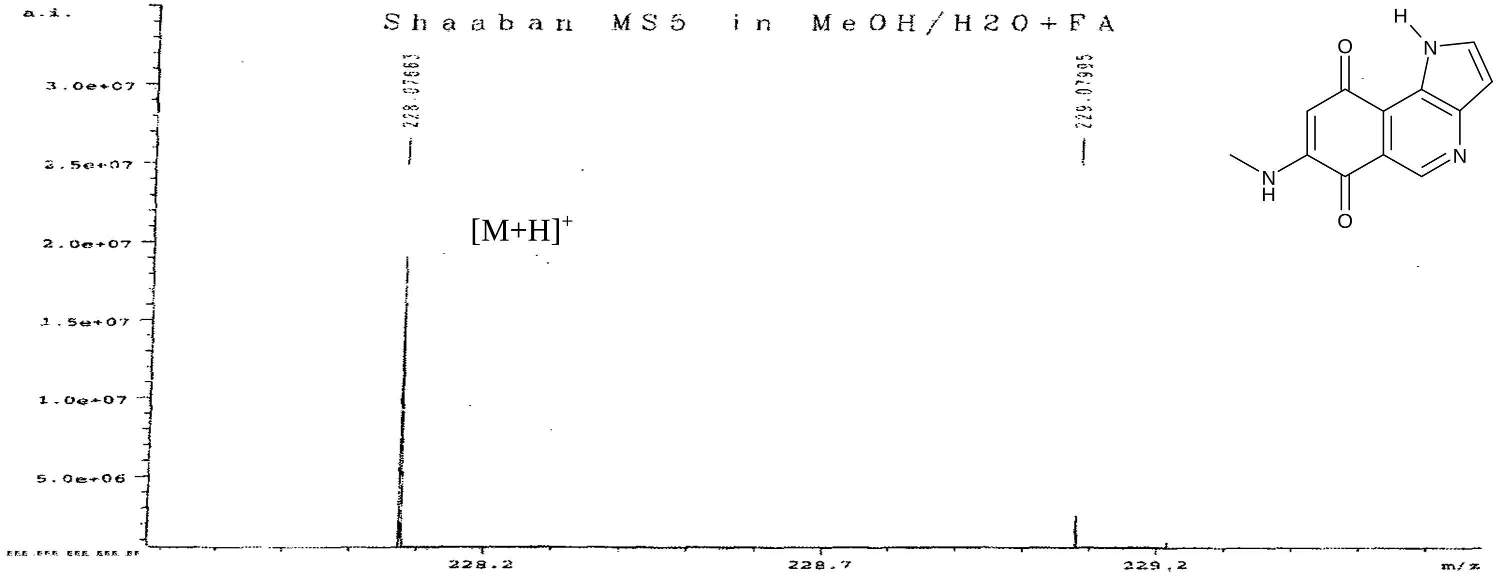

Figure S19: (+)-HRESI-MS spectrum of Mansouramycin F (**2a**).

11.11.04 17:24:32

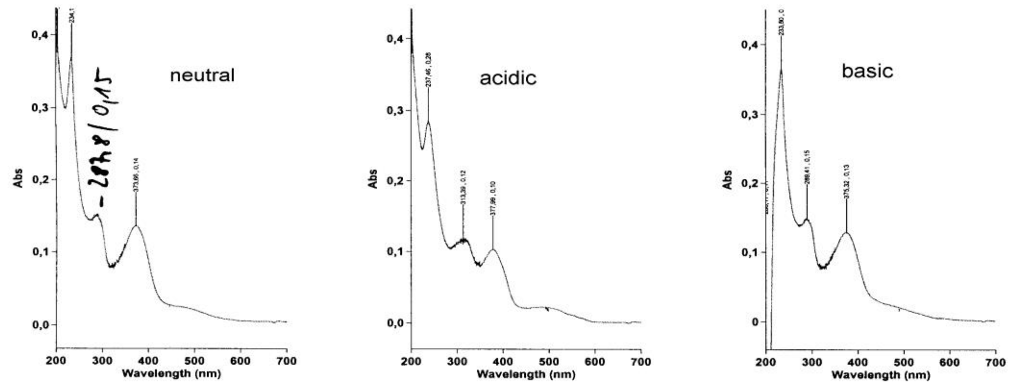

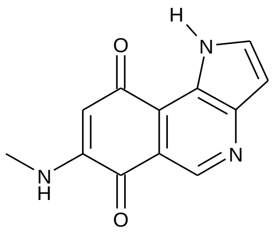

Instrument	Cary 300
Instrument Version	9,00
Start (nm)	700,00
Stop (nm)	200,00
X Mode	Nanometers
Y Mode	Abs
UV-Vis Scan Rate (nm/min)	199,800
UV-Vis Data Interval (nm)	0,333
UV-Vis Ave. Time (sec)	0,100
UV-Vis SBW (nm)	2,0
Beam Mode	Double
Signal-to-noise Mode	Off
UV Source	On
Vis Source	On
Source Changeover (nm)	350,00
Baseline Correction	Off
Cycle Mode	Off
Accessory	Cell changer
Comments	MS 5a<br>c=0.094 mg / 10.0 mL Methanol

Sample Name: neutral
Collection Time

Peak Table
Peak Style Peaks
Peak Threshold 0,0100
Range 700,00nm to 199,83nm

Wavelength (nm)	Abs
373,66	0,1357
234,13	0,3688
203,50	0,3880
202,16	0,4008

Sample Name: acidic
Collection Time

Peak Table
Peak Style Peaks
Peak Threshold 0,0100
Range 700,00nm to 199,83nm

Wavelength (nm)	Abs
377,99	0,1030
313,39	0,1184
237,46	0,2845
202,83	0,4035
200,50	0,4055

Sample Name: basic
Collection Time

Peak Table
Peak Style Peaks
Peak Threshold 0,0100
Range 700,00nm to 199,83nm

Wavelength (nm)	Abs
375,32	0,1286
289,41	0,1494
233,80	0,3638
200,17	0,1058

Figure S20: UV spectra of Mansouramycin F (**2a**) in methanol.

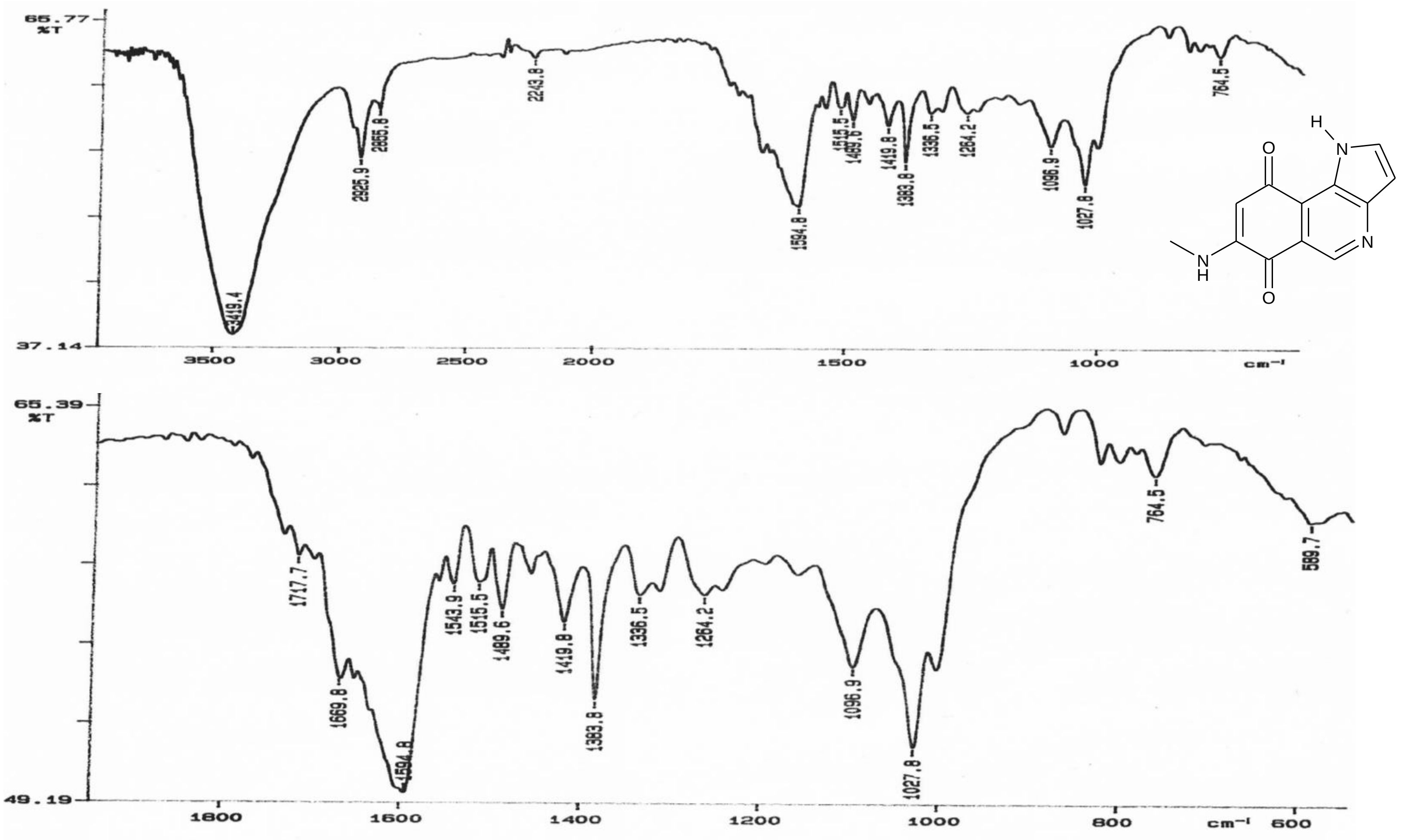

Figure S21: IR (KBr) spectrum of Mansouramycin F (**2a**).

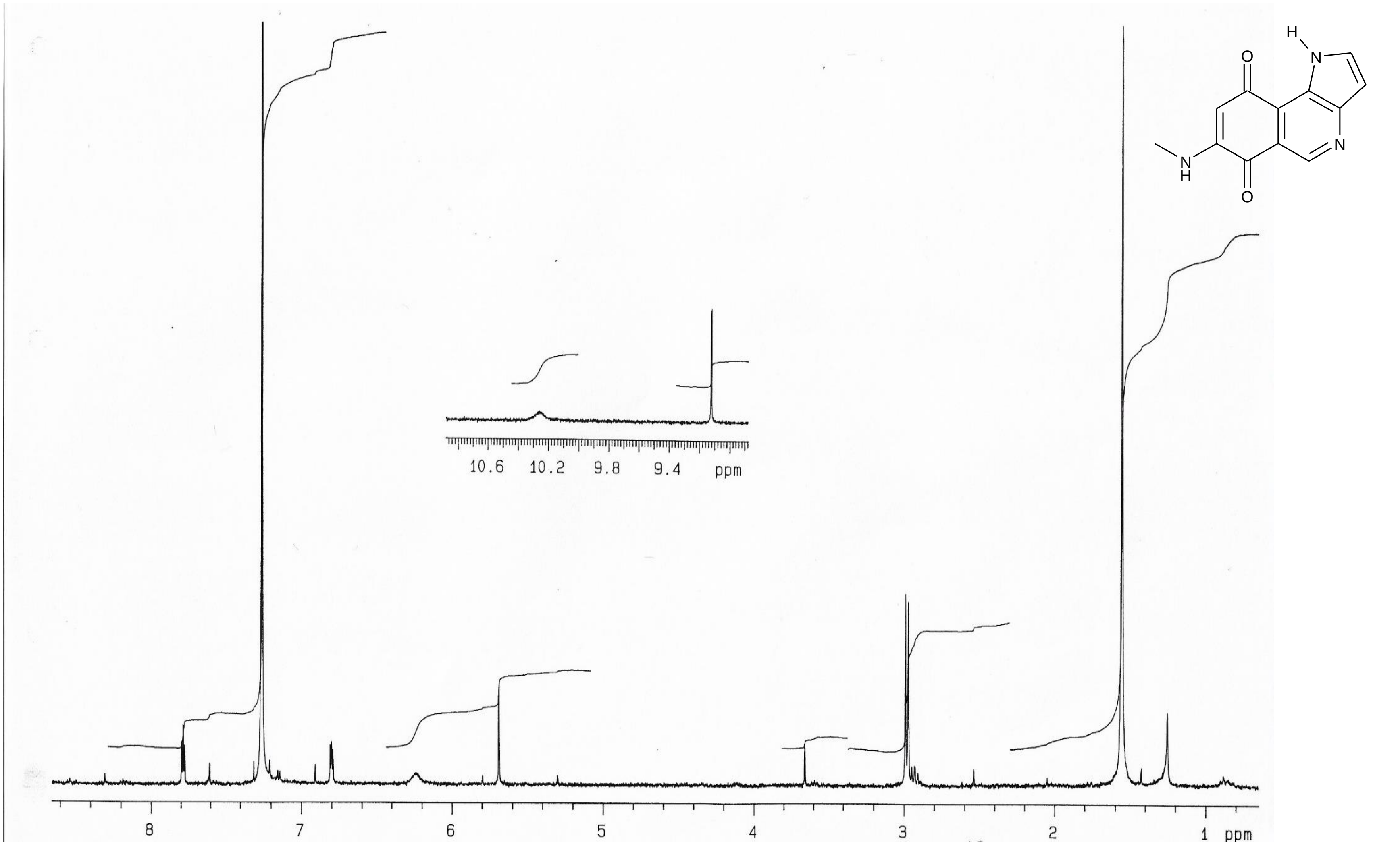

Figure S22: ^{1}H NMR ($CDCl_3$, 300 MHz) spectrum of Mansouramycin F (**2a**).

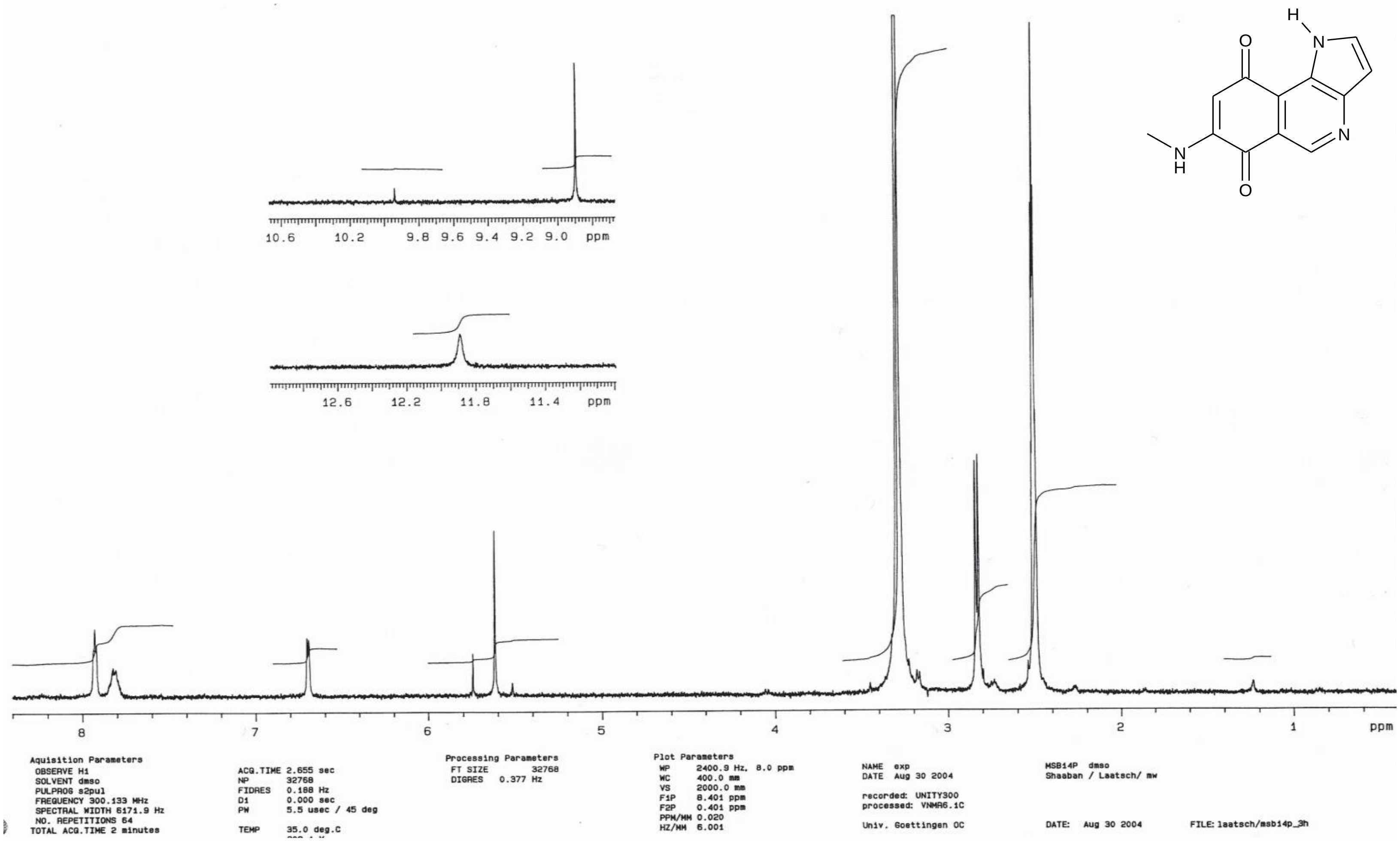

Figure S23: ^{1}H NMR (DMSO-d_6, 300 MHz) spectrum of Mansouramycin F (**2a**).

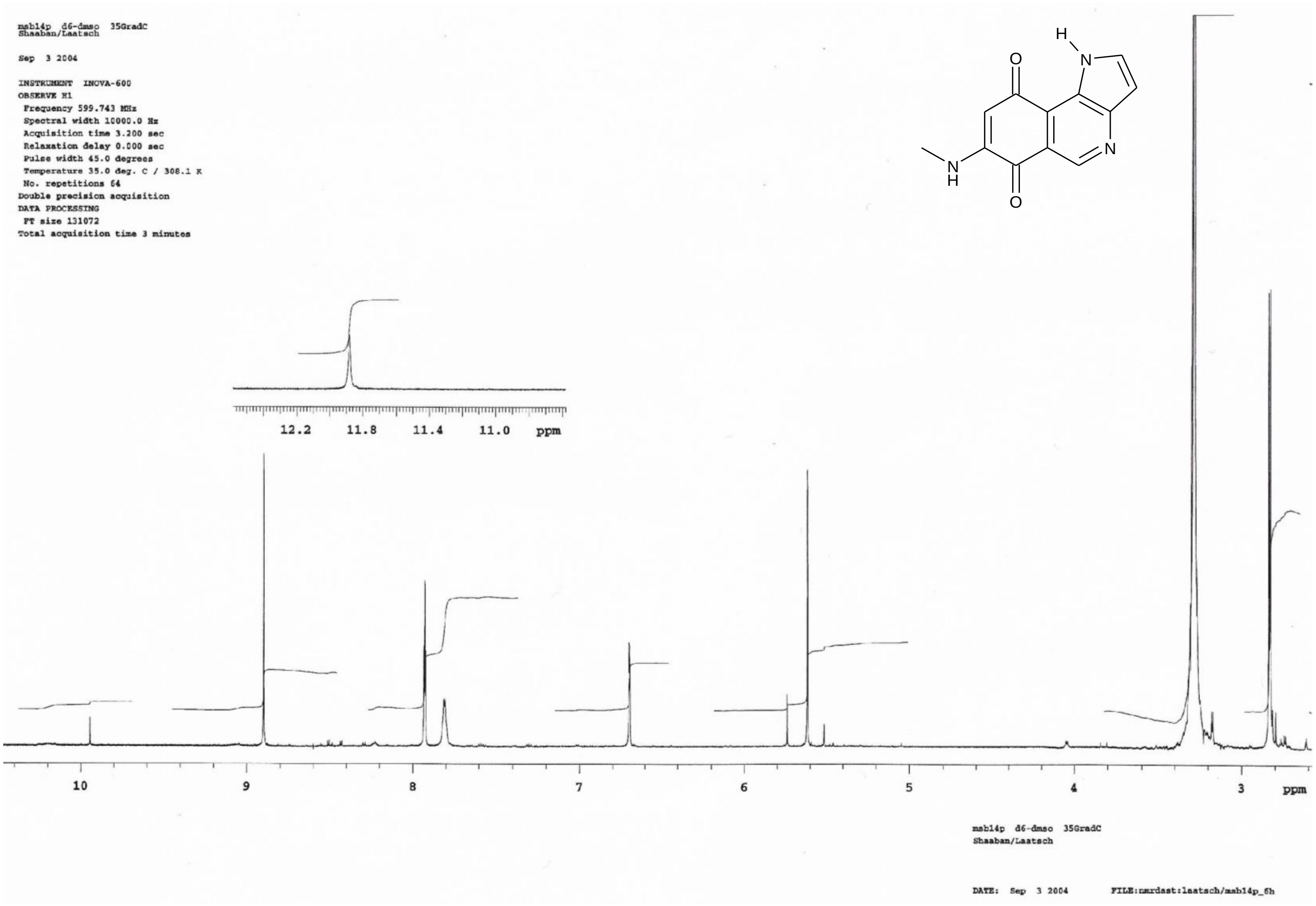

Figure S24: ^{1}H NMR (DMSO-d_6, 600 MHz) spectrum of Mansouramycin F (**2a**).

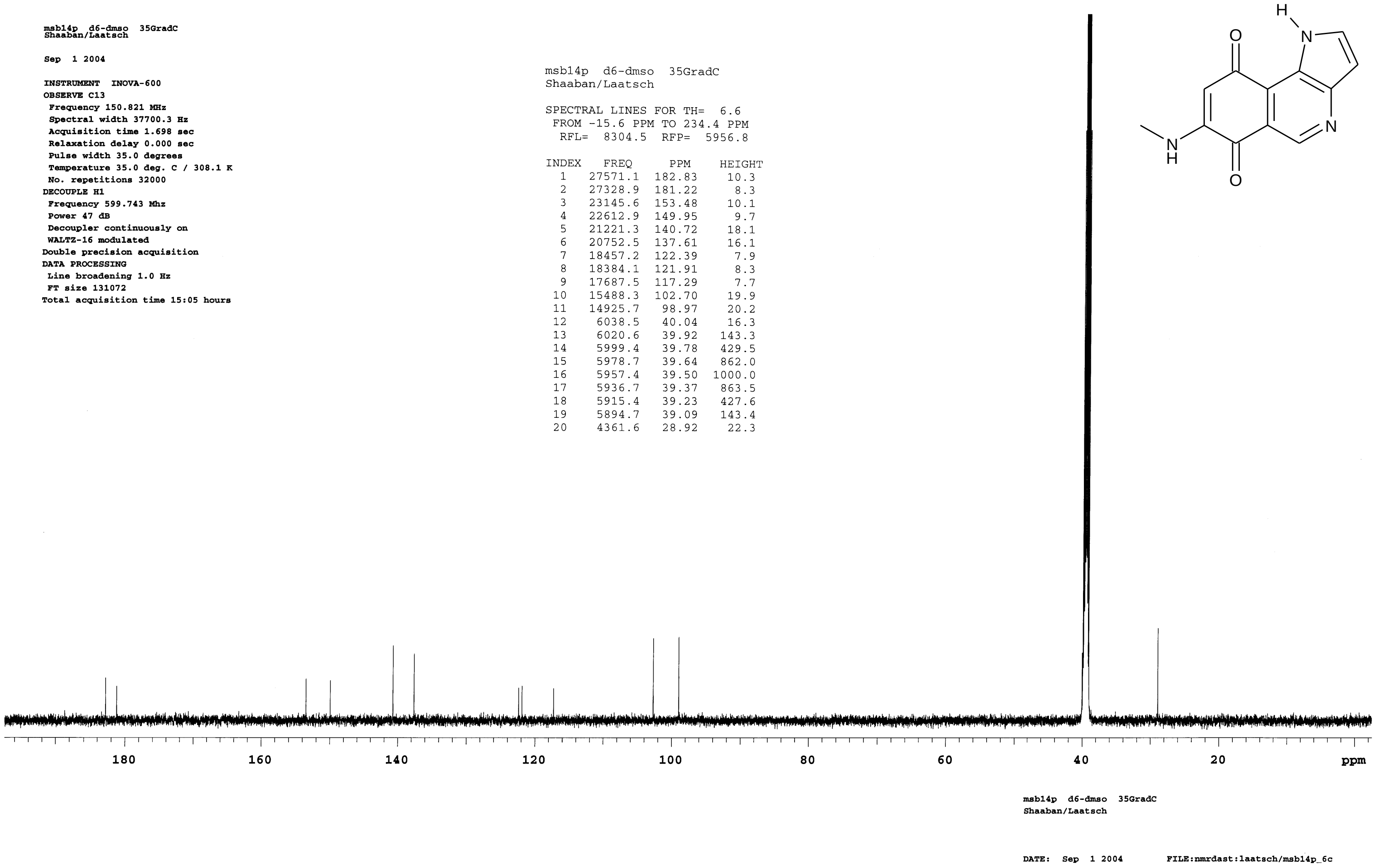

INDEX	FREQ	PPM	HEIGHT
1	27571.1	182.83	10.3
2	27328.9	181.22	8.3
3	23145.6	153.48	10.1
4	22612.9	149.95	9.7
5	21221.3	140.72	18.1
6	20752.5	137.61	16.1
7	18457.2	122.39	7.9
8	18384.1	121.91	8.3
9	17687.5	117.29	7.7
10	15488.3	102.70	19.9
11	14925.7	98.97	20.2
12	6038.5	40.04	16.3
13	6020.6	39.92	143.3
14	5999.4	39.78	429.5
15	5978.7	39.64	862.0
16	5957.4	39.50	1000.0
17	5936.7	39.37	863.5
18	5915.4	39.23	427.6
19	5894.7	39.09	143.4
20	4361.6	28.92	22.3

Figure S25: ^{13}C NMR (DMSO-d_6, 150 MHz) spectrum of Mansouramycin F (**2a**).

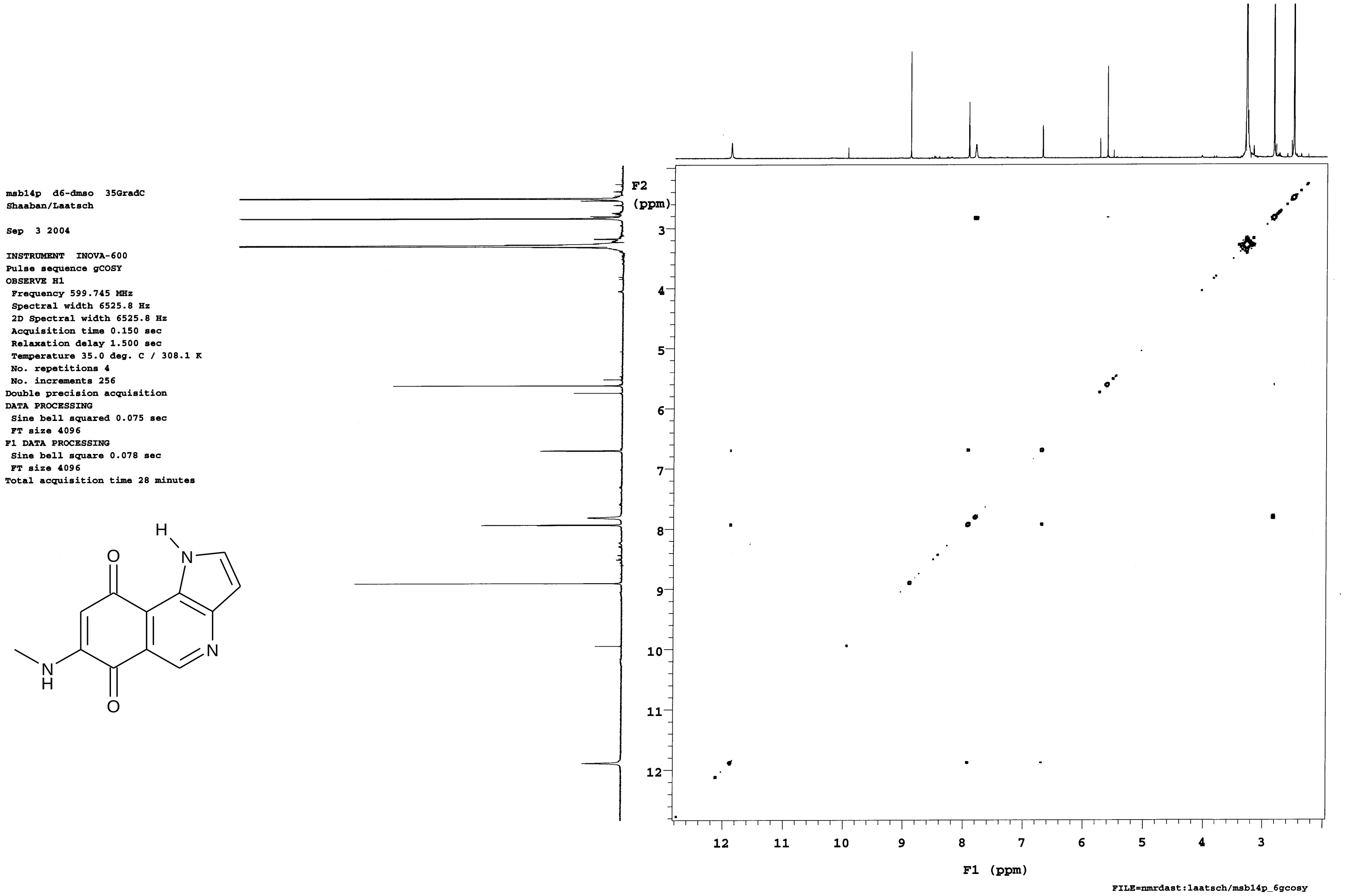

Figure S26: $^{1}H,^{1}H$-COSY (DMSO-d_6, 600 MHz) spectrum of Mansouramycin F (**2a**).

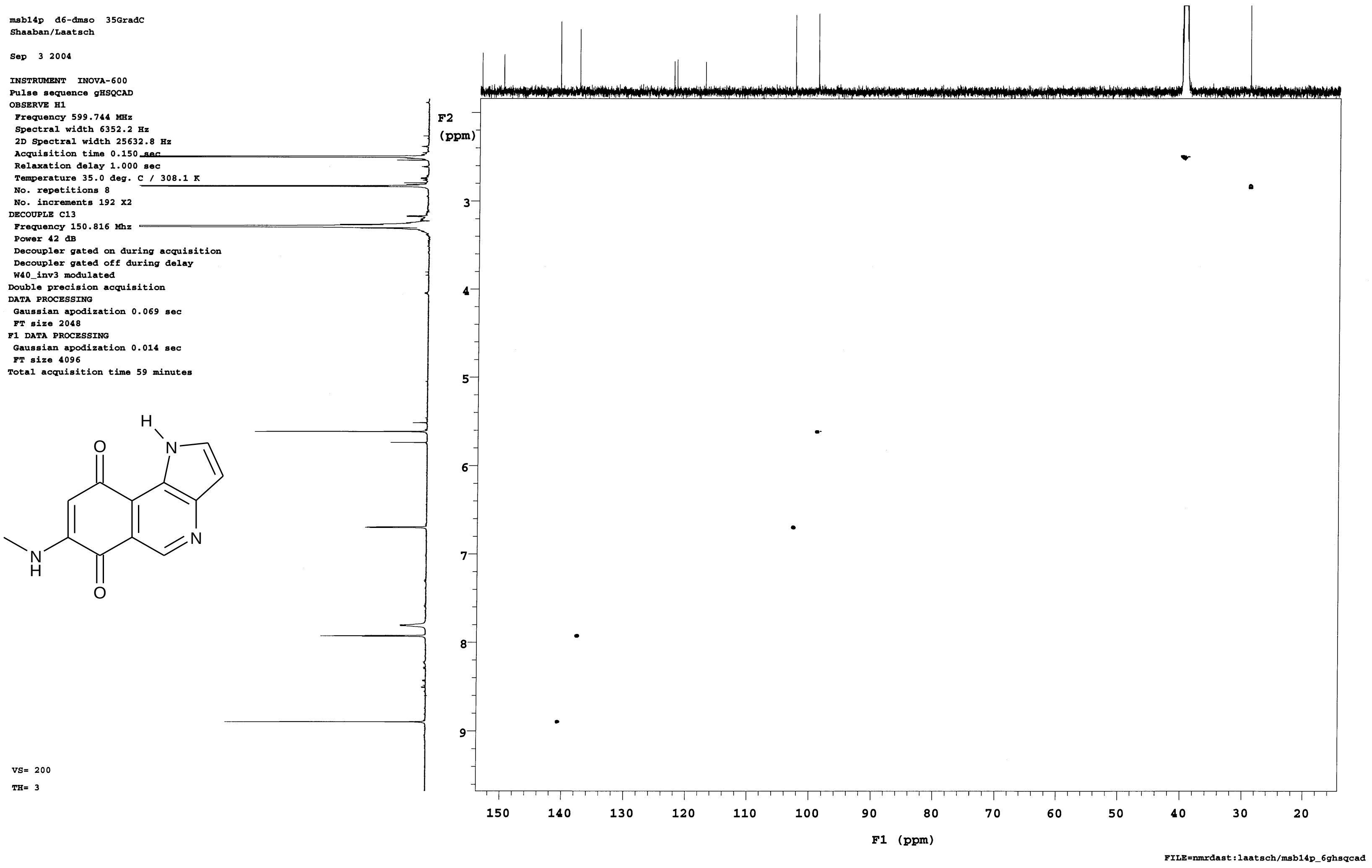

Figure S27: HSQC (DMSO-d_6, 600 MHz) spectrum of Mansouramycin F (**2a**).

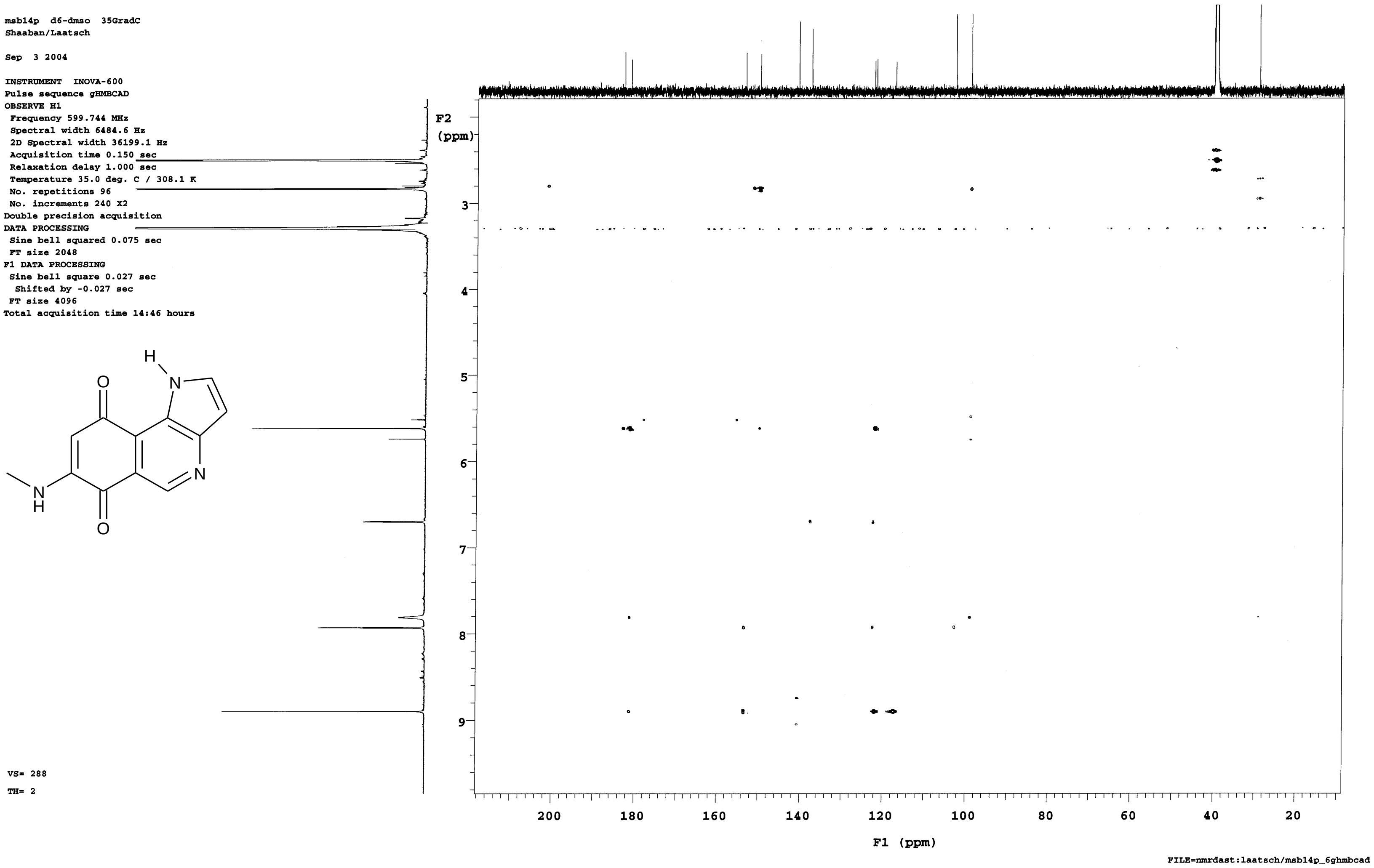

Figure S28: HMBC (DMSO-d_6, 600 MHz) spectrum of Mansouramycin F (**2a**).

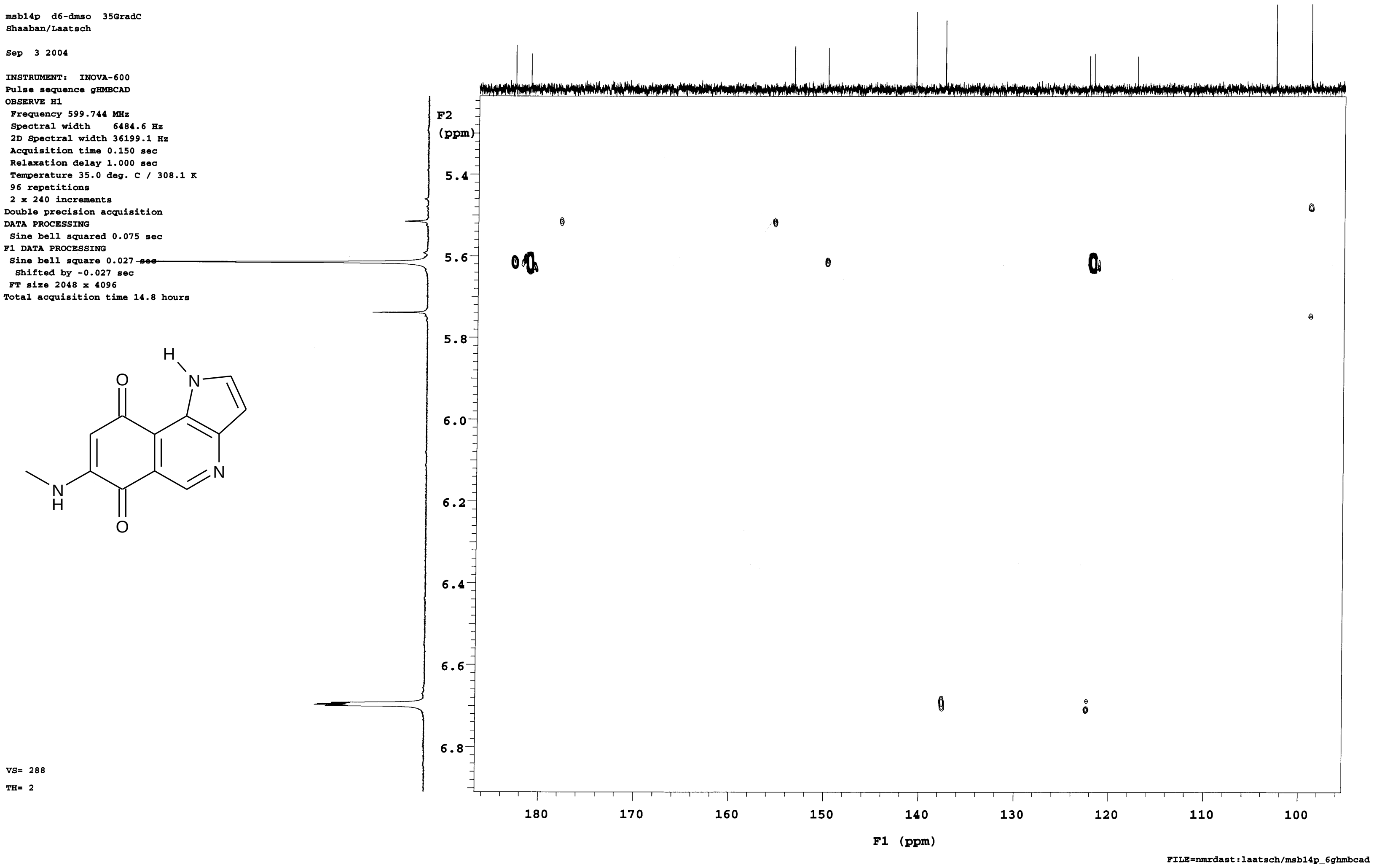

Figure S29: HMBC (DMSO-d_6, 600 MHz) spectrum of Mansouramycin F (**2a**).

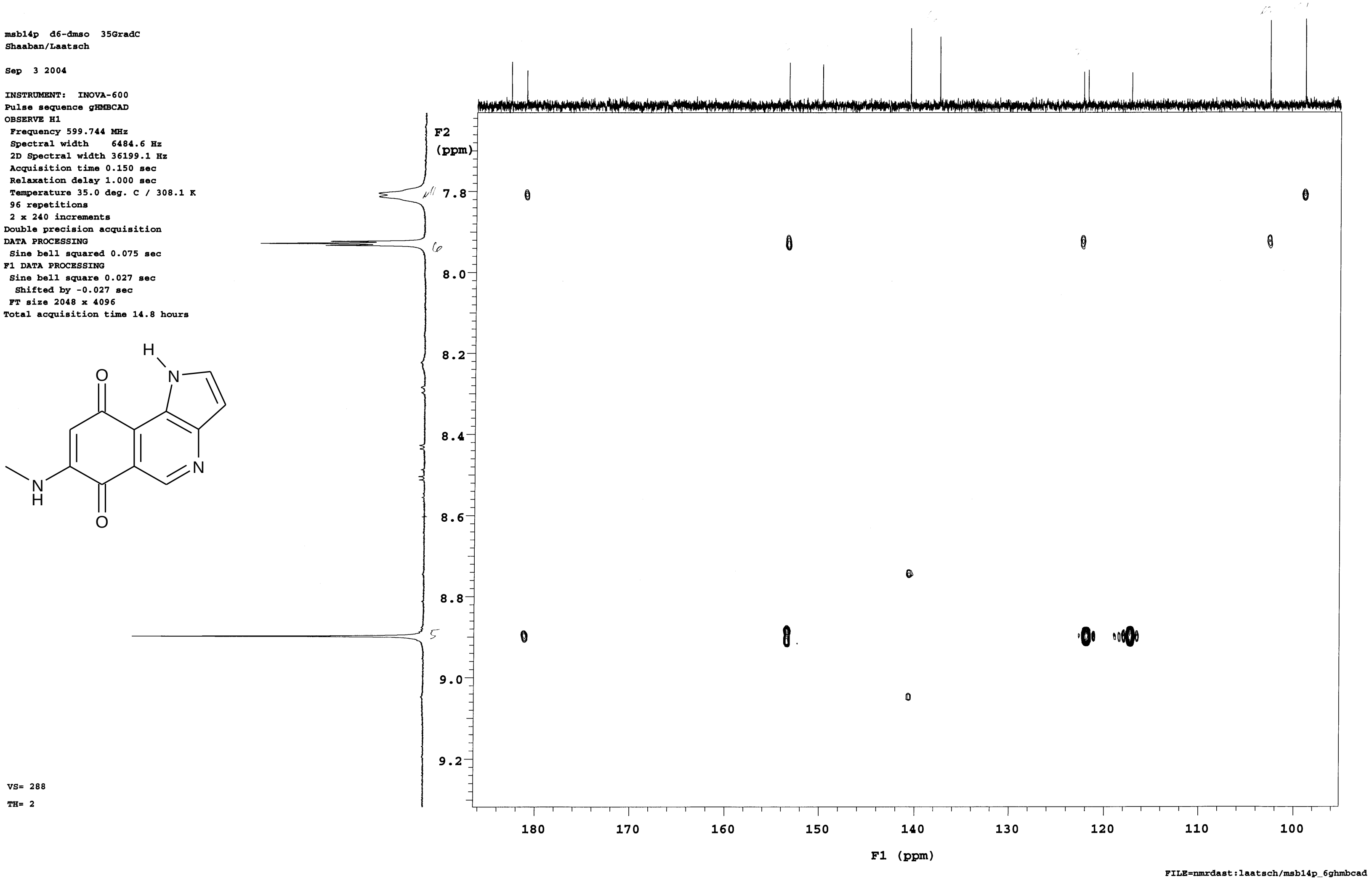

Figure S30: HMBC (DMSO-d_6, 600 MHz) spectrum of Mansouramycin F (**2a**).

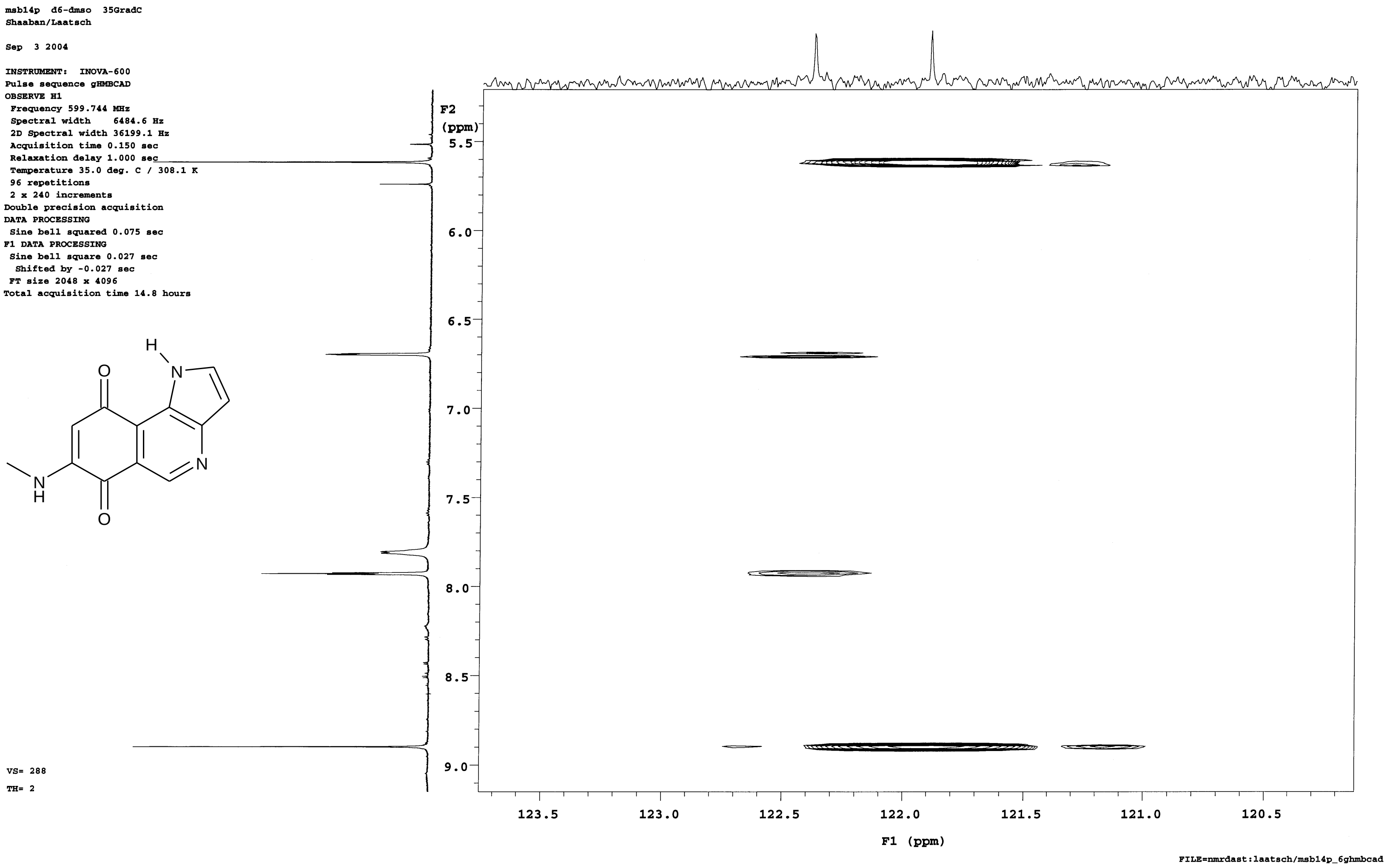

Figure S31: HMBC (DMSO-d_6, 600 MHz) spectrum of Mansouramycin F (**2a**).

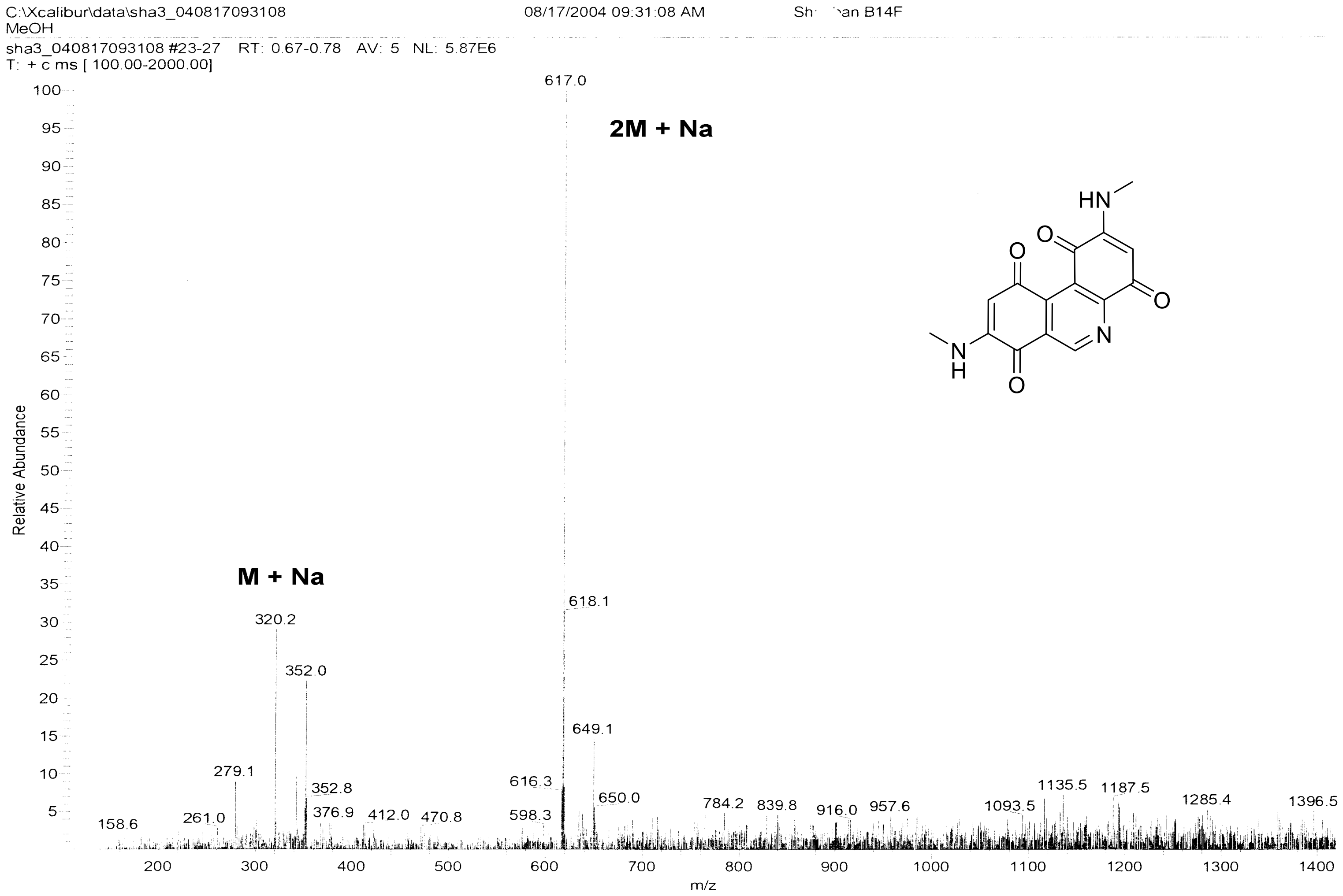

Figure S32: (+)-ESI-MS spectrum of Mansouramycin G (**3a**).

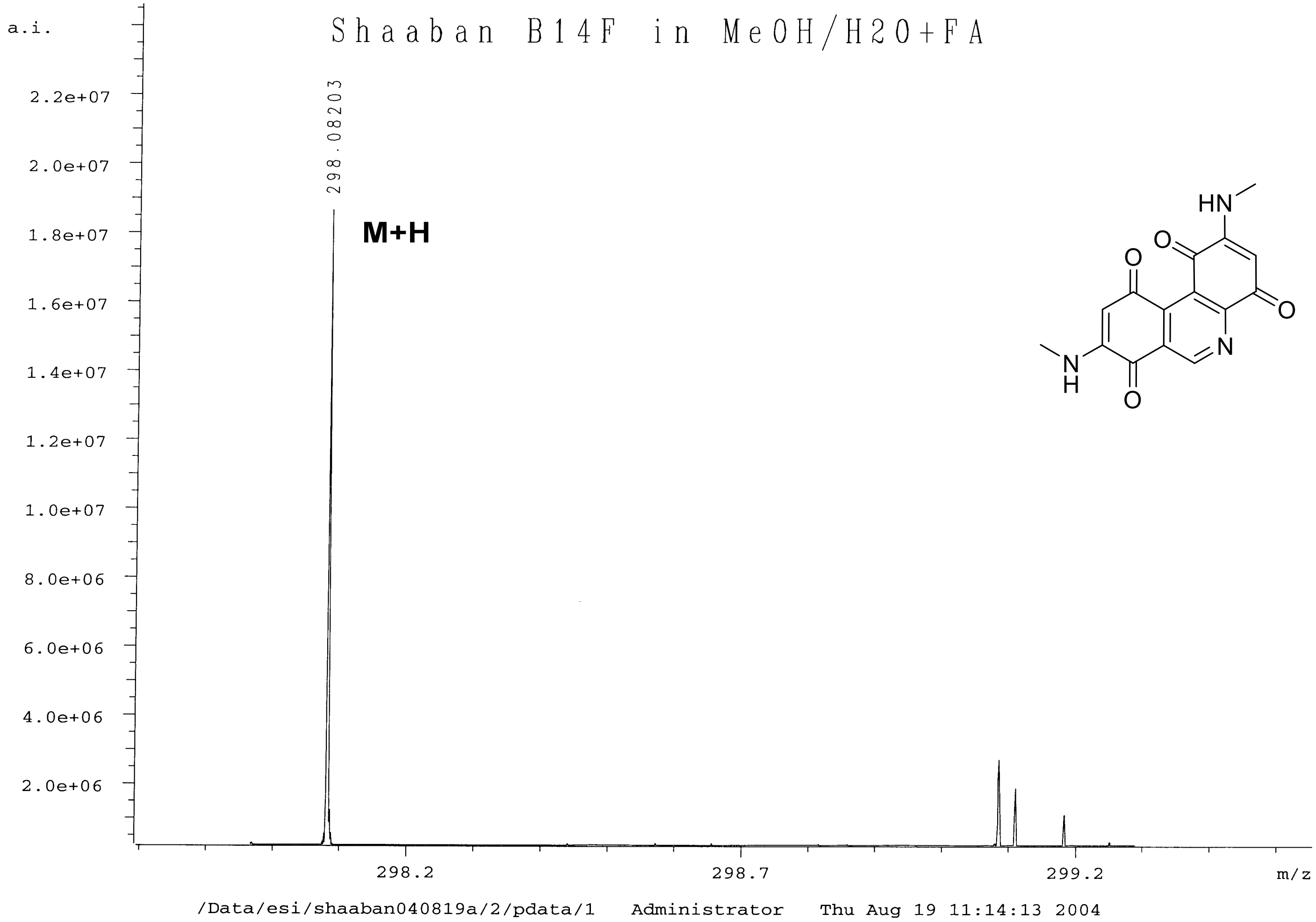

Figure S33: (+)-HRESI-MS spectrum of Mansouramycin G (**3a**).

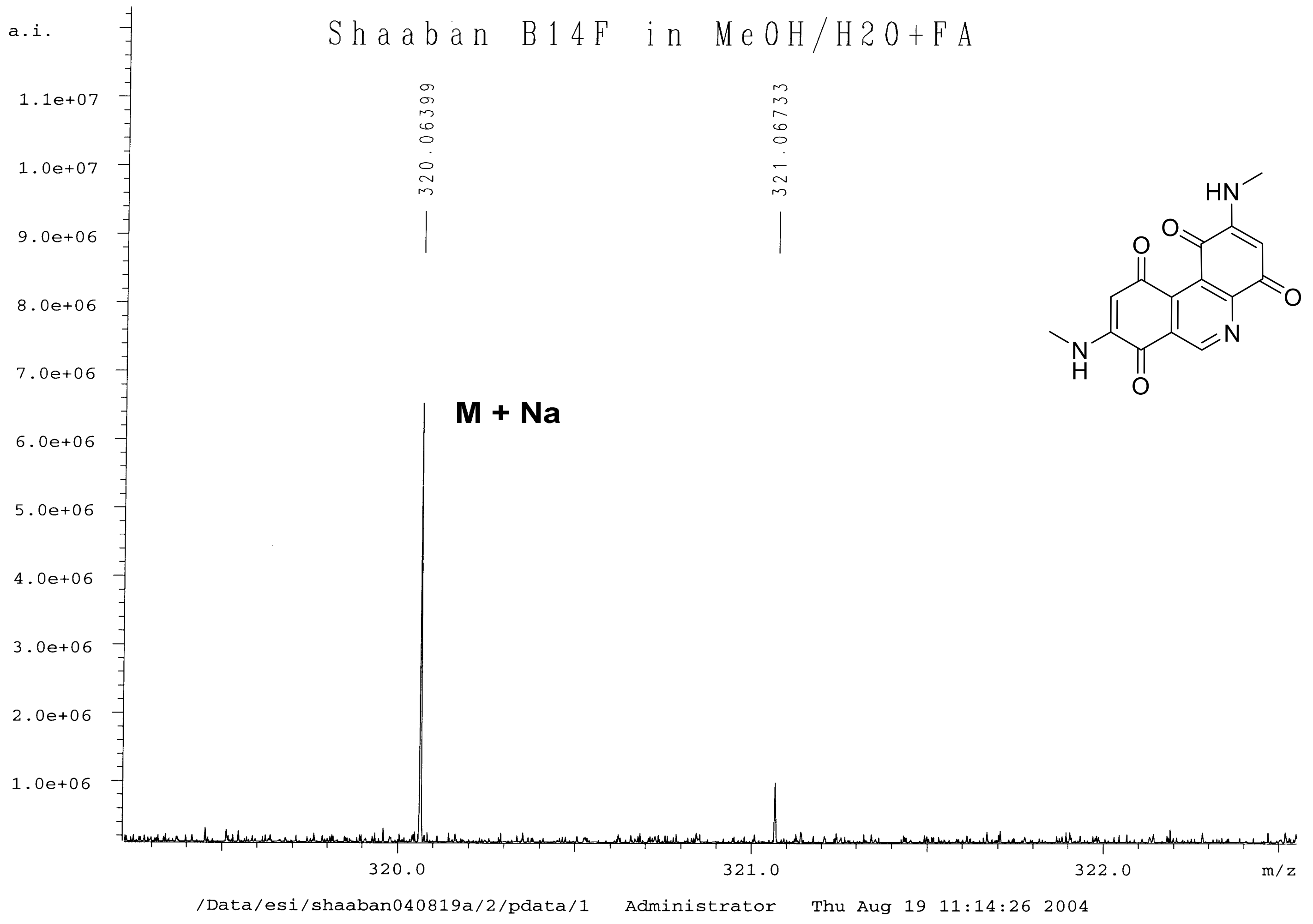

Figure S34: (+)-HRESI-MS spectrum of Mansouramycin G (**3a**).

11.11.04 13:46:05

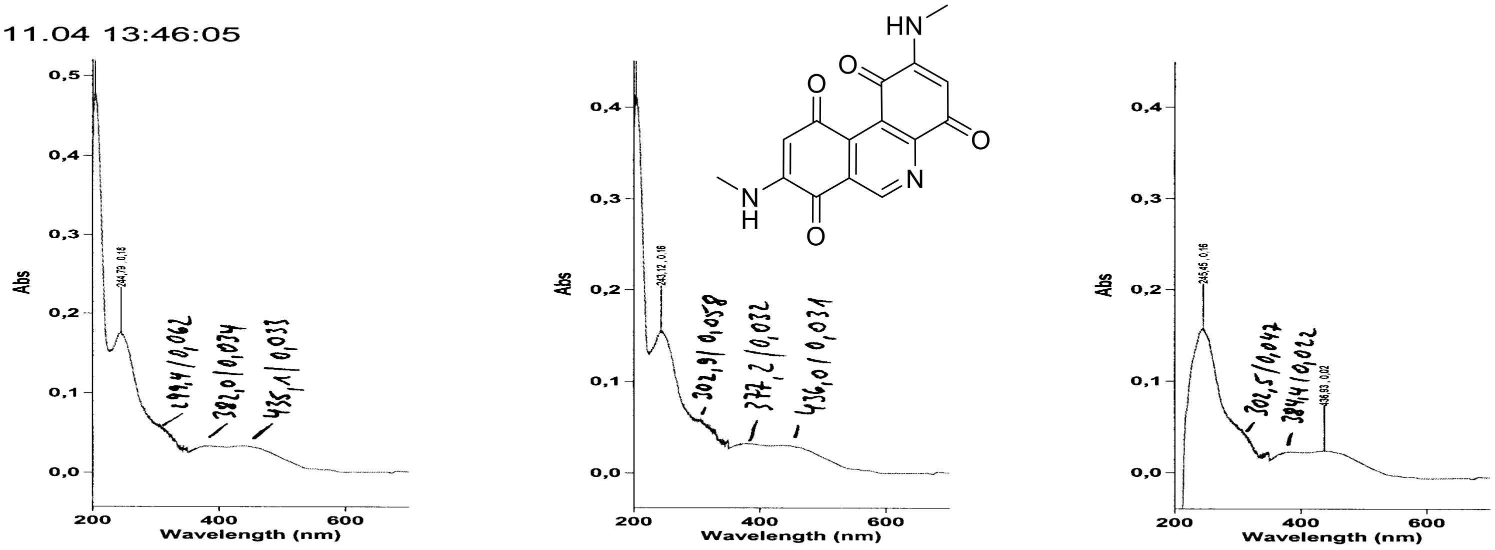

Scan Analysis Report

Report Time : Do 11 Nov 01:44:38 PM 2004
Batch: C:\UV-Daten\Laatsch\B14F.BSW
Software version: 02.00(25)
Operator:

Instrument Parameters

Instrument	Cary 300
Instrument Version	9,00
Start (nm)	700,00
Stop (nm)	200,00
X Mode	Nanometers
Y Mode	Abs
UV-Vis Scan Rate (nm/min)	199,800
UV-Vis Data Interval (nm)	0,333
UV-Vis Ave. Time (sec)	0,100
UV-Vis SBW (nm)	2,0
Beam Mode	Double
Signal-to-noise Mode	Off
UV Source	On
Vis Source	On
Source Changeover (nm)	350,00
Baseline Correction	Off
Cycle Mode	Off
Accessory	Cell changer
Comments	B14F<br>c=0.040 mg/10.0 mL Methanol

Sample Name: neutral

Wavelength (nm)	Abs
244,79	0,1763
203,50	0,4777

Sample Name: sauer

Collection Time

Peak Table
Peak Style
Peak Threshold
Range

Wavelength (nm)	Abs
243,12	0,1554
202,83	0,4138

Sample Name: basisch

Collection Time

Peak Table
Peak Style
Peak Threshold
Range

Wavelength (nm)	Abs
436,93	0,0242
245,45	0,1573

Figure S35: UV spectra of Mansouramycin G (**3a**) in methanol.

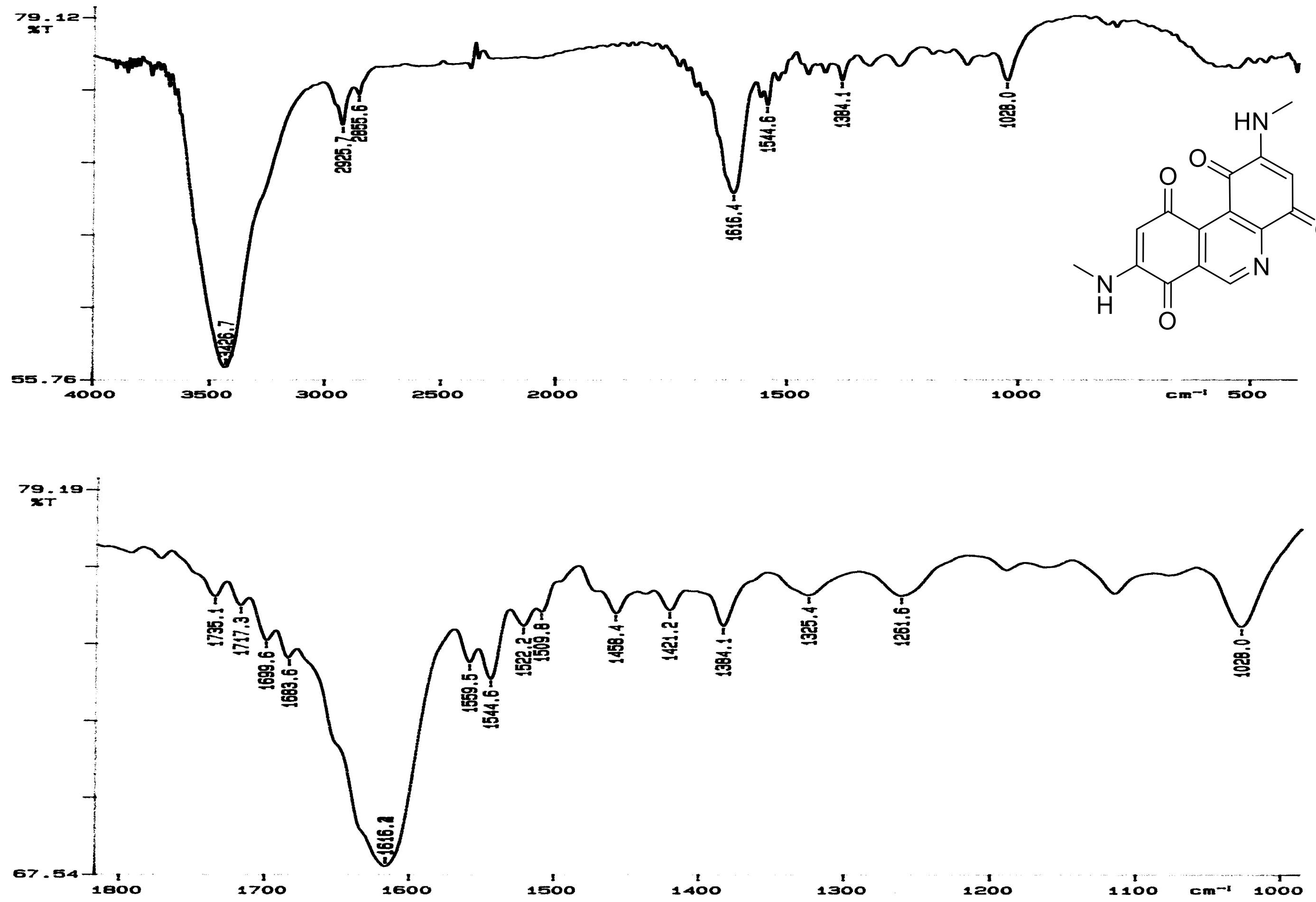

Figure S36: IR (KBr) spectra of Mansouramycin G (**3a**).

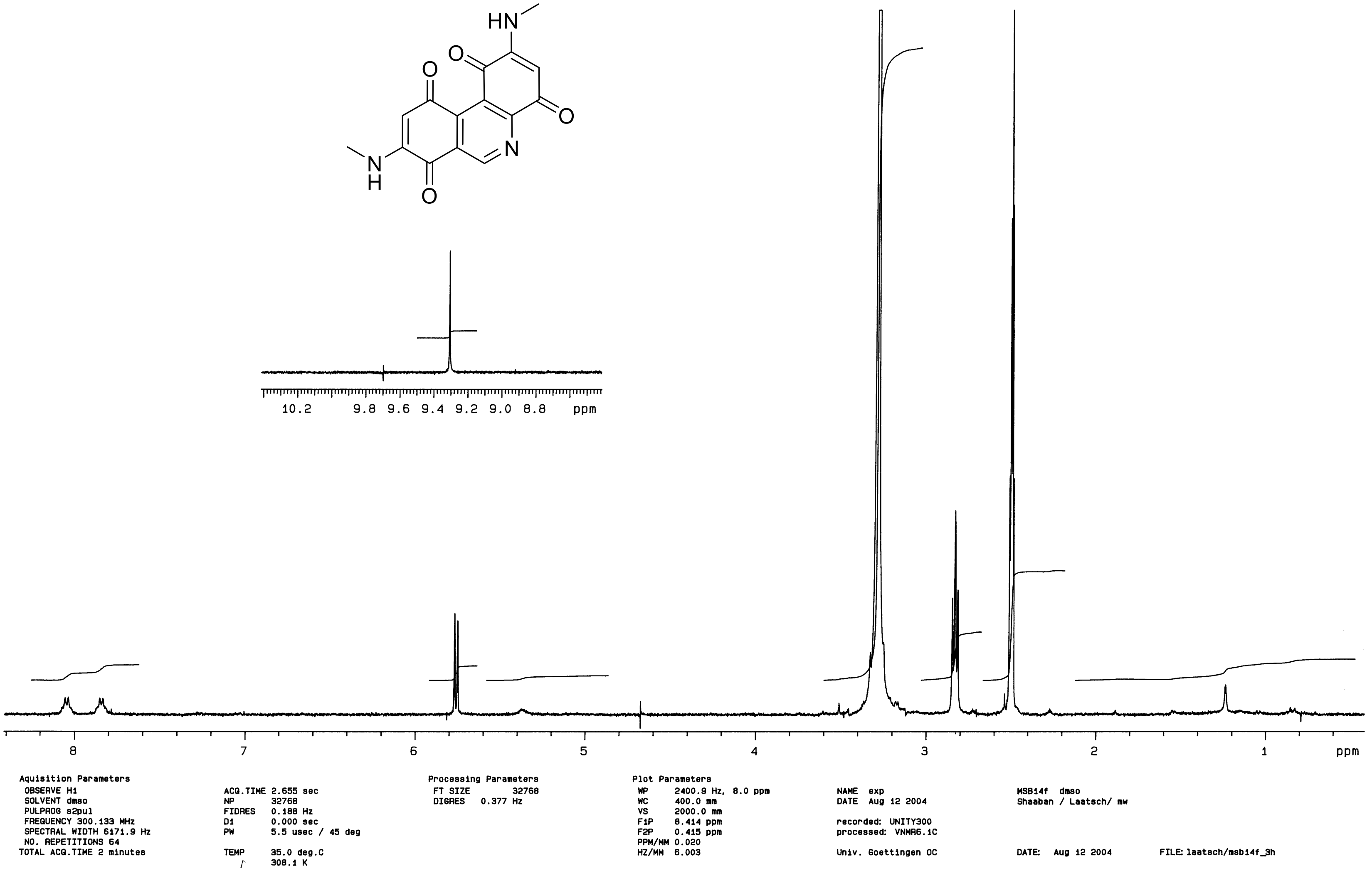

Figure S37: ^{1}H NMR (DMSO-d_6, 300 MHz) spectrum of Mansouramycin G (**3a**).

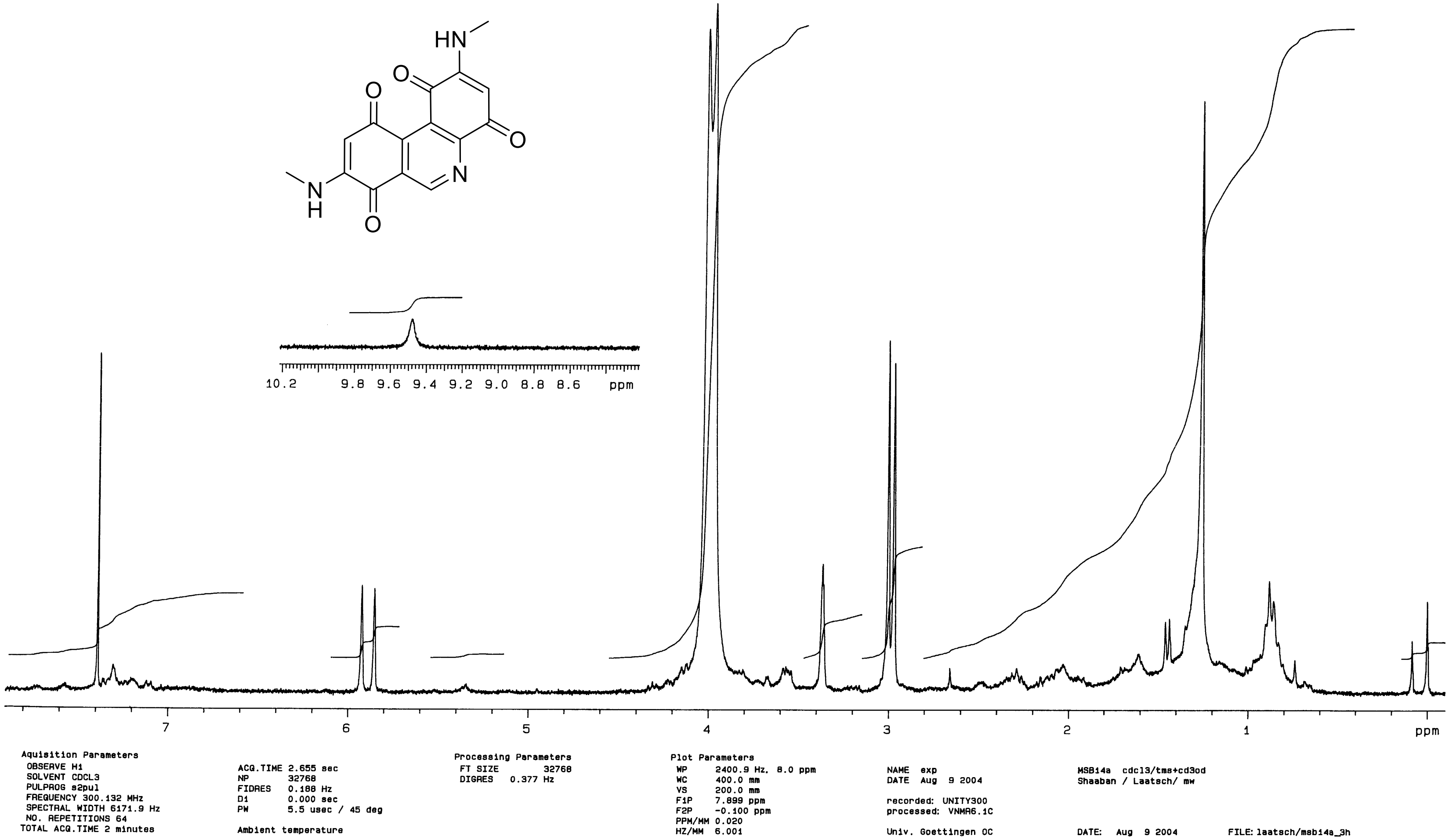

Figure S38: ^{1}H NMR (CD_3OD, 300 MHz) spectrum of Mansouramycin G (**3a**).

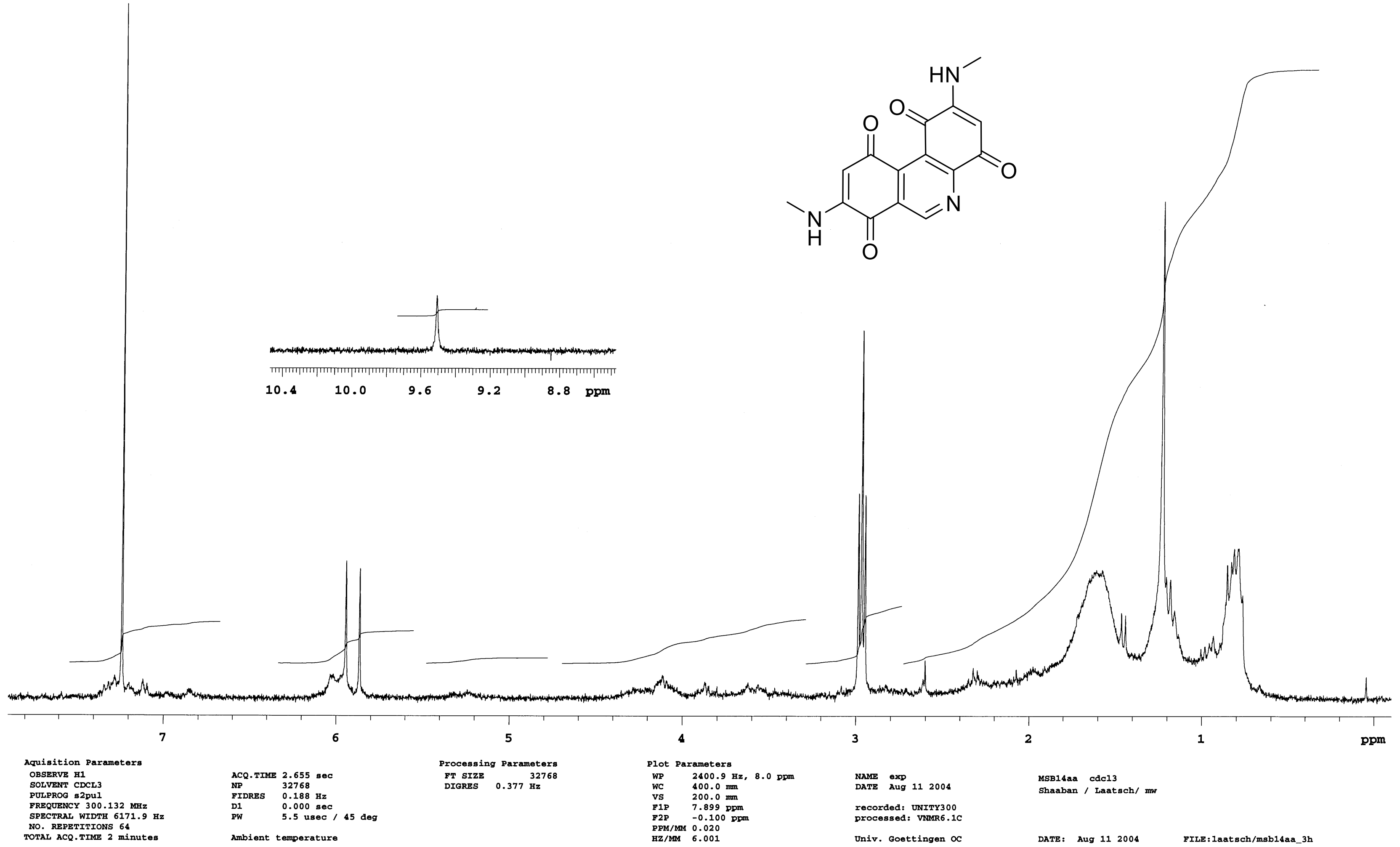

Figure S39: ^{1}H NMR ($CDCl_3$, 300 MHz) spectrum of Mansouramycin G (**3a**).

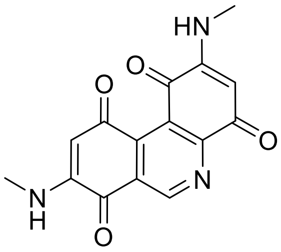

MSB14f dmso-d6
Shaaban / Laatsch

Aug 19 2004

INSTRUMENT INOVA-600
OBSERVE C13
Frequency 150.821 MHz
Spectral width 37700.3 Hz
Acquisition time 1.698 sec
Relaxation delay 0.000 sec
Pulse width 35.0 degrees
Temperature 35.0 deg. C / 308.1 K
No. repetitions 20480
DECOUPLE H1
Frequency 599.743 Mhz
Power 47 dB
Decoupler continuously on
WALTZ-16 modulated
Double precision acquisition
DATA PROCESSING
Line broadening 1.0 Hz
FT size 131072
Total acquisition time 9:39 hours

INDEX	FREQ	PPM	HEIGHT
1	27284.6	180.93	7.6
2	27108.0	179.76	9.3
3	26876.1	178.22	10.5
4	26790.4	177.65	6.4
5	23130.6	153.38	8.9
6	22951.1	152.19	8.8
7	22675.0	150.36	15.8
8	22461.0	148.94	14.4
9	21341.6	141.52	7.5
10	19145.8	126.96	6.1
11	19102.6	126.67	8.4
12	15201.8	100.80	15.9
13	15025.2	99.63	10.6
14	6956.6	46.13	5.4
15	6036.2	40.03	17.8
16	6018.3	39.91	149.4
17	5997.6	39.77	435.5
18	5976.4	39.63	855.4
19	5955.6	39.49	1000.0
20	5934.4	39.35	852.0
21	5913.6	39.21	429.2
22	5892.4	39.07	142.6
23	4376.0	29.02	19.1
24	4363.3	28.93	23.4

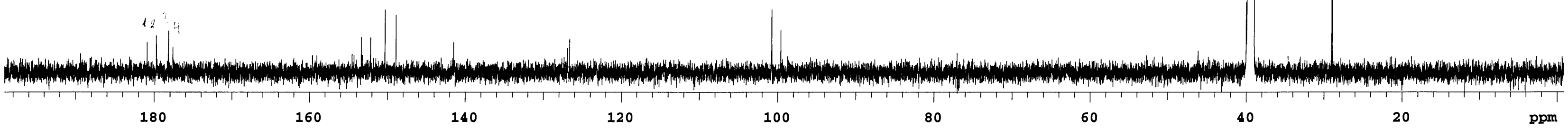

MSB14f dmso-d6
Shaaban / Laatsch

DATE: Aug 19 2004 FILE:nmrdast:laatsch/msb14f_6c

Figure S40: ^{13}C NMR (DMSO-d_6, 150 MHz) spectrum of Mansouramycin G (**3a**).

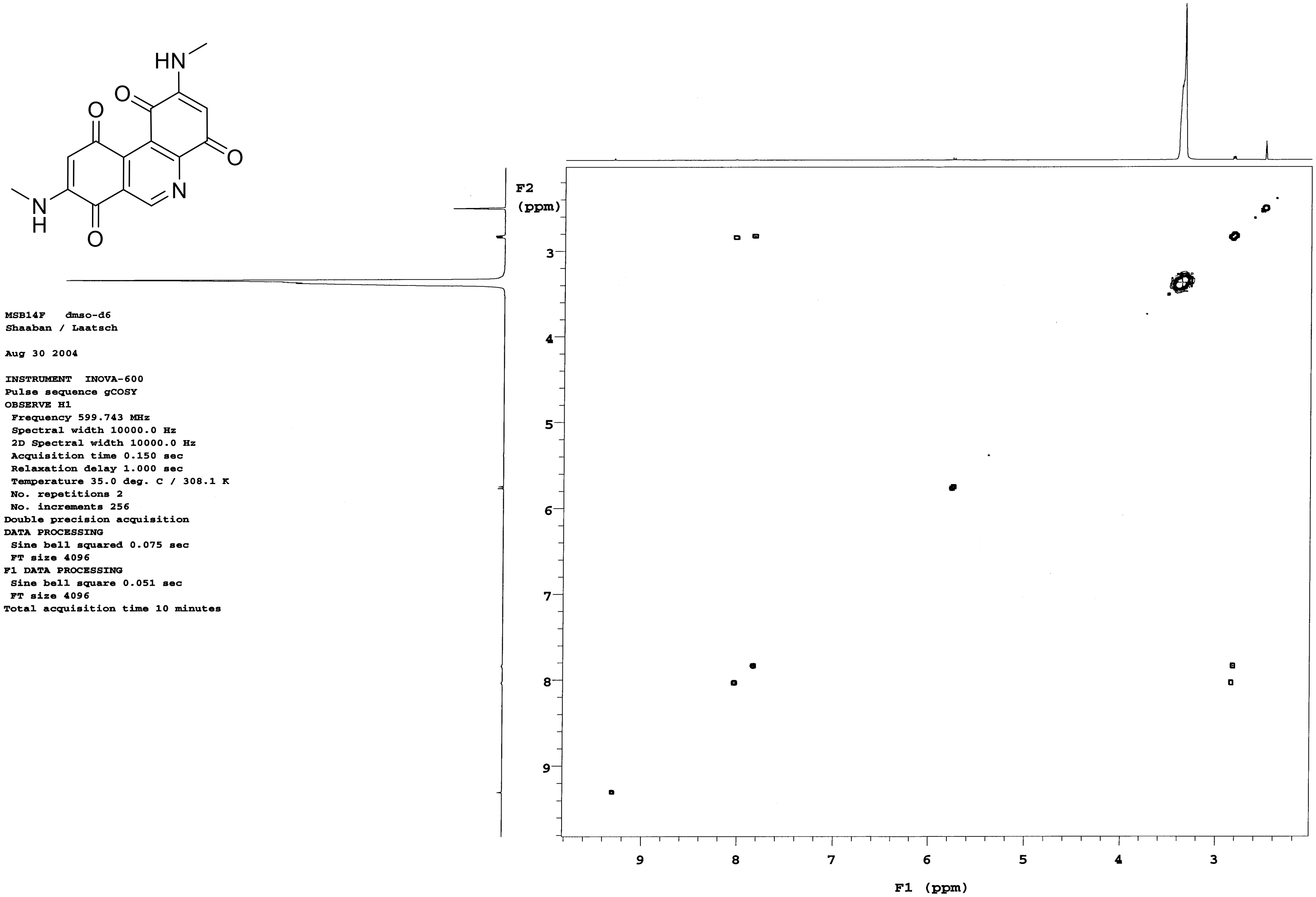

Figure S41: ^{1}H,^{1}H-COSY (DMSO-d_6, 600 MHz) spectrum of Mansouramycin G (**3a**).

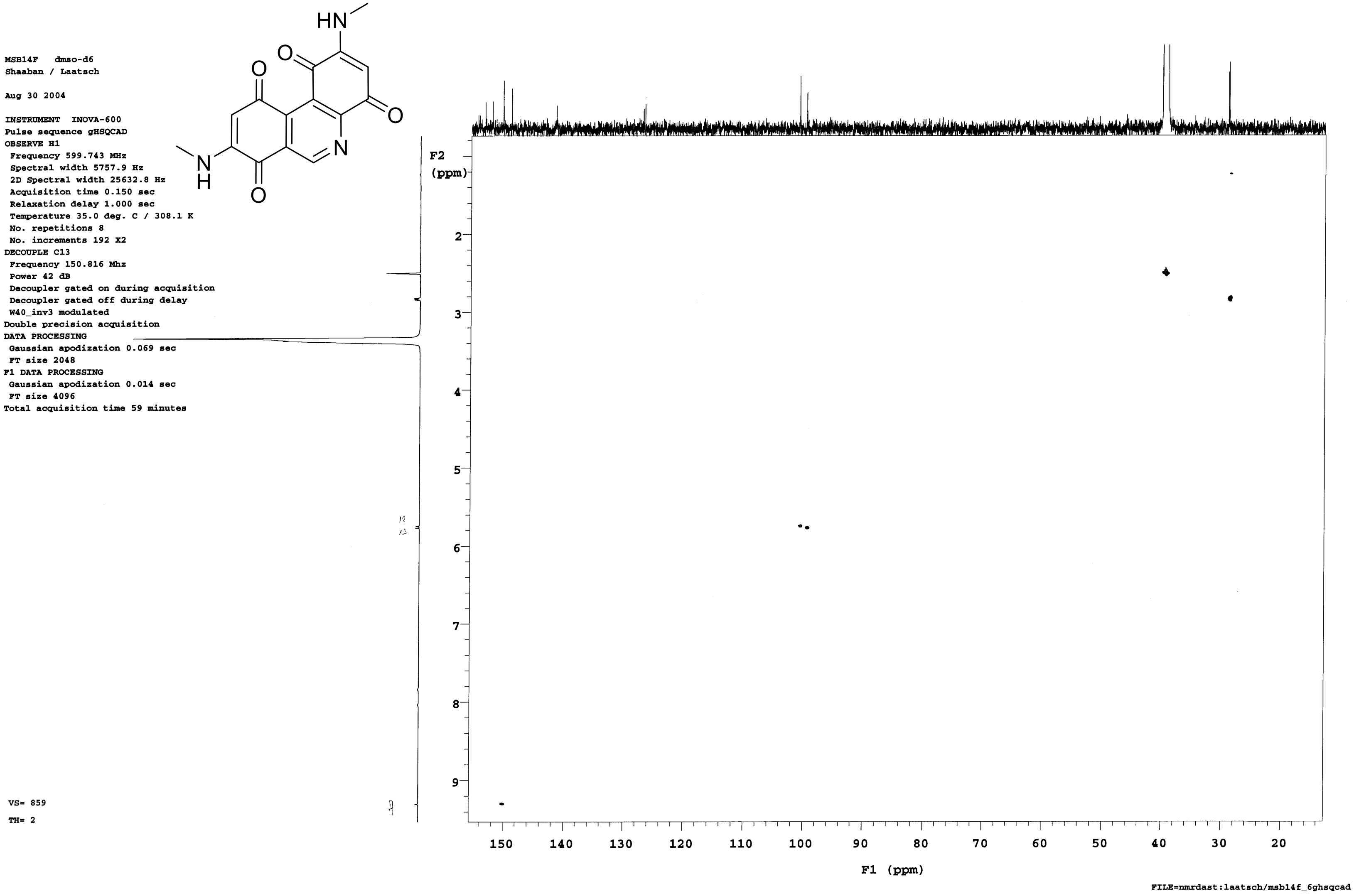

Figure S42: HSQC (DMSO-d_6, 600 MHz) spectrum of Mansouramycin G (**3a**)

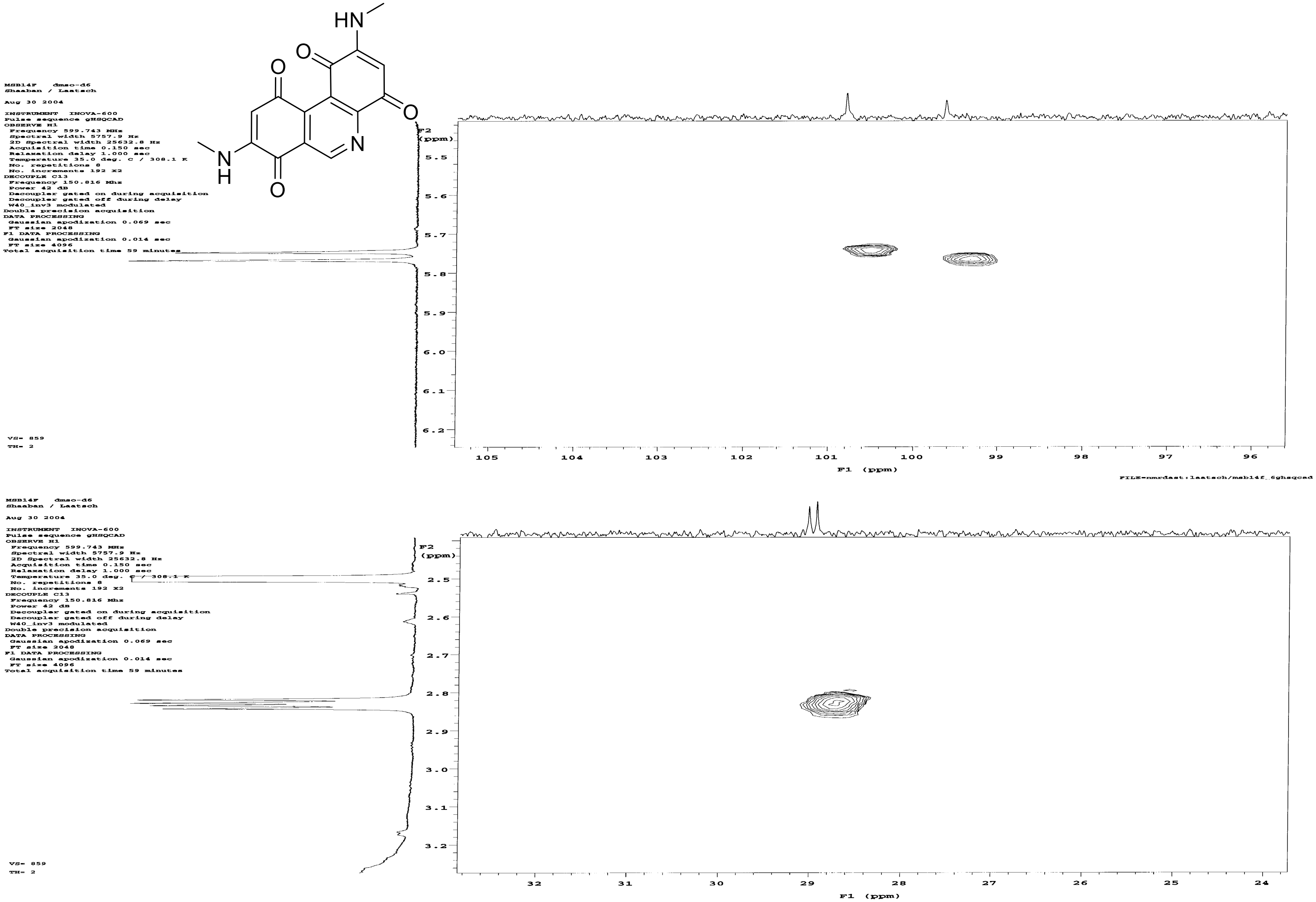

Figure S43: HSQC (DMSO-d_6, 600 MHz) spectrum of Mansouramycin G (**3a**).

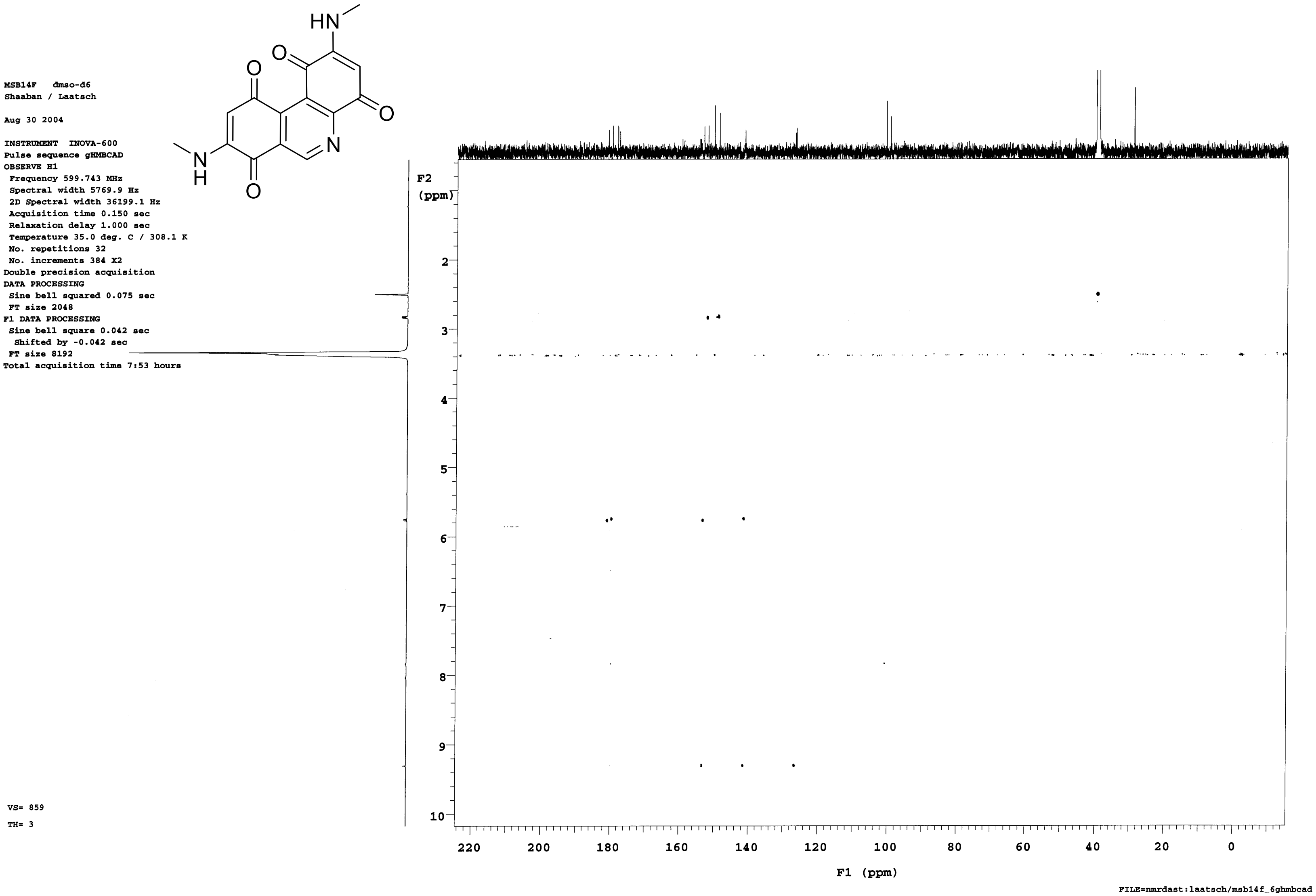

Figure S44: HMBC (DMSO-d_6, 600 MHz) spectrum of Mansouramycin G (**3a**).

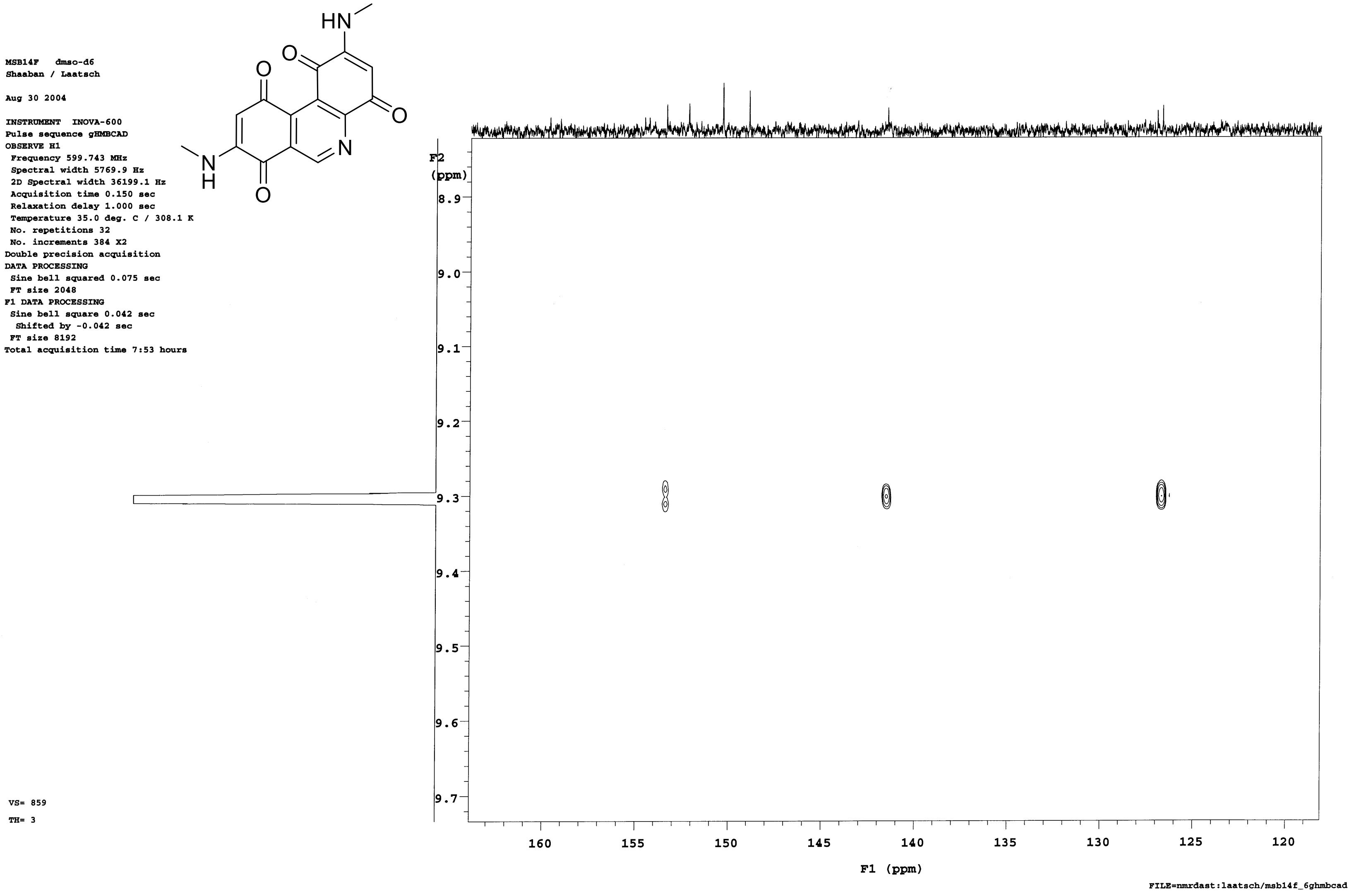

Figure S45: HMBC (DMSO-d_6, 600 MHz) spectrum of Mansouramycin G (**3a**).

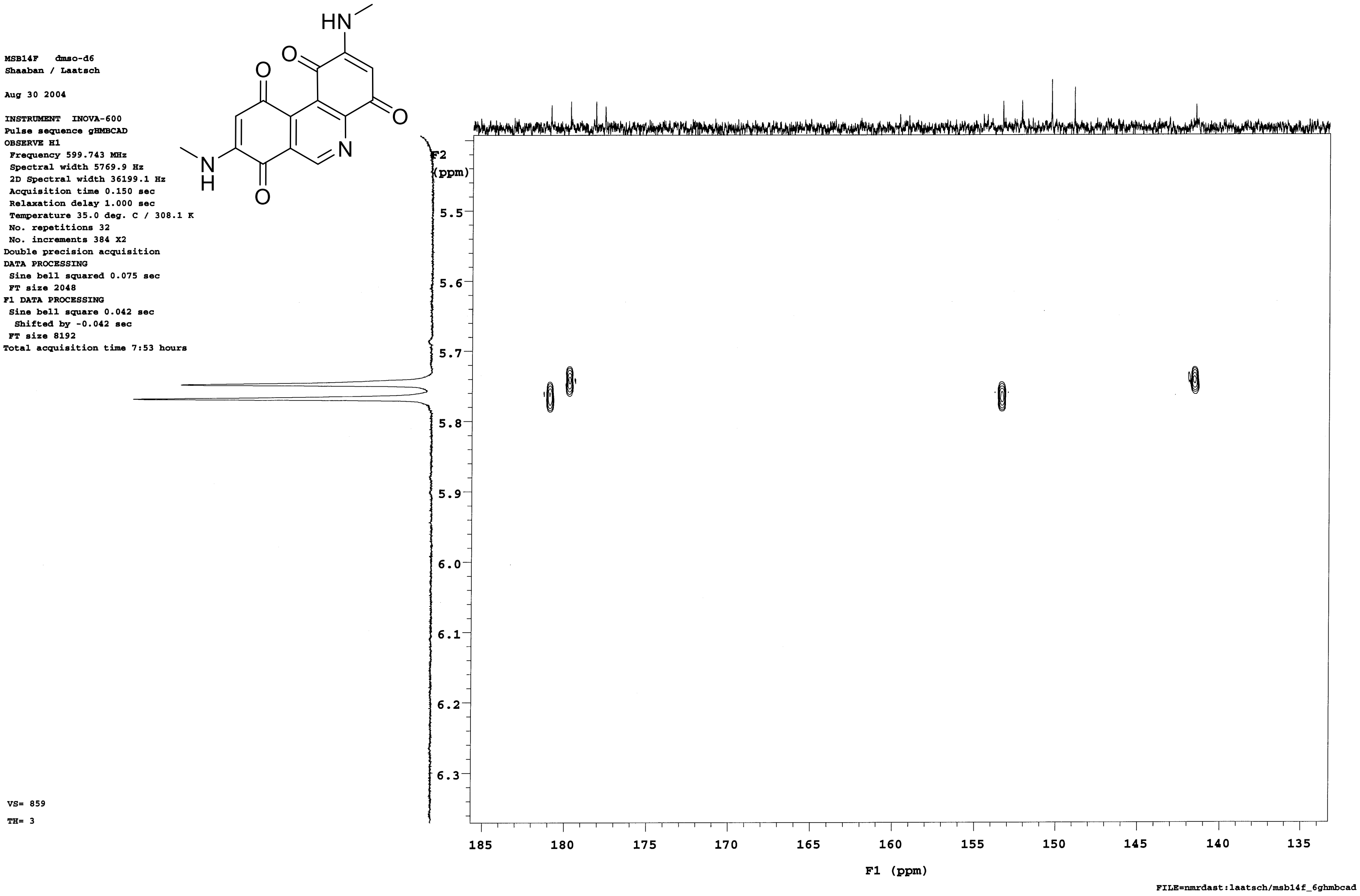

Figure S46: HMBC (DMSO-d_6, 600 MHz) spectrum of Mansouramycin G (**3a**).

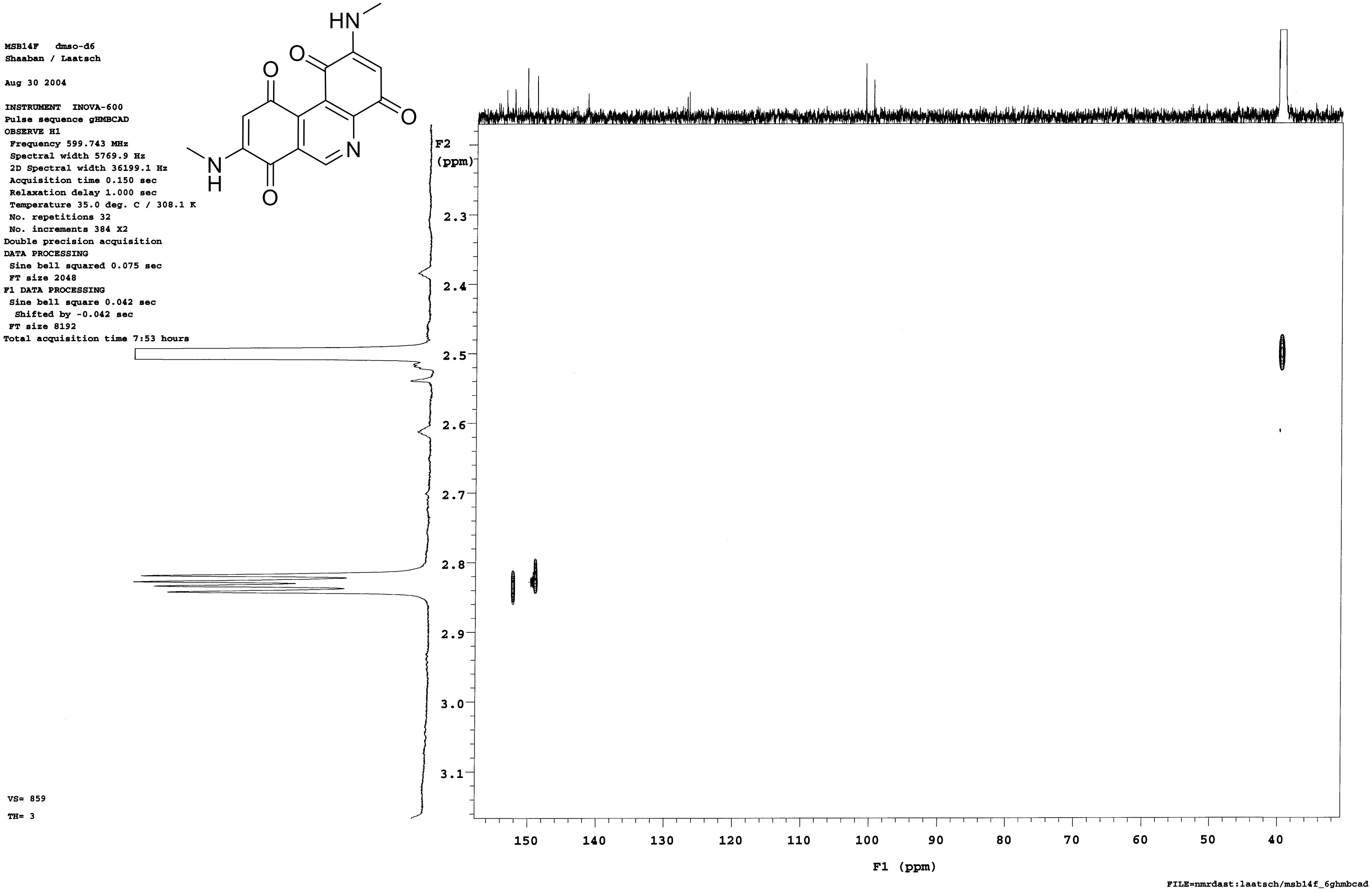

Figure S47: HMBC (DMSO-d_6, 600 MHz) spectrum of Mansouramycin G (**3a**).
